# C(sp^3^)–C(sp^3^) coupling of non-activated alkyl-iodides with electron-deficient alkenes *via* visible-light/silane-mediated alkyl-radical formation†

**DOI:** 10.1039/d2sc03516b

**Published:** 2022-10-28

**Authors:** Sanesh Mistry, Roopender Kumar, Andrew Lister, Matthew J. Gaunt

**Affiliations:** Yusuf Hamied Department of Chemistry, University of Cambridge Lensfield Road Cambridge CB2 1EW UK mjg32@cam.ac.uk; Oncology R&D, AstraZeneca Cambridge CB4 0WG UK

## Abstract

Here, we present a remarkably mild and general initiation protocol for alkyl-radical generation from non-activated alkyl-iodides. An interaction between a silane and an alkyl iodide is excited by irradiation with visible light to trigger carbon–iodide bond homolysis and form the alkyl radical. We show how this method can be developed into an operationally simple and general Giese addition reaction that can tolerate a range of sensitive functionalities not normally explored in established approaches to this strategically important transformation. The new method requires no photocatalyst or other additives and uses only commerical tris(trimethylsilyl)silane and visible light to effectively combine a broad range of alkyl halides with activated alkenes to form C(sp^3^)–C(sp^3^) bonds embedded within complex frameworks.

## Abstract 2

Here, we present a remarkably mild and general initiation protocol for alkyl-radical generation from non-activated alkyl-iodides.

## Graphical abstract

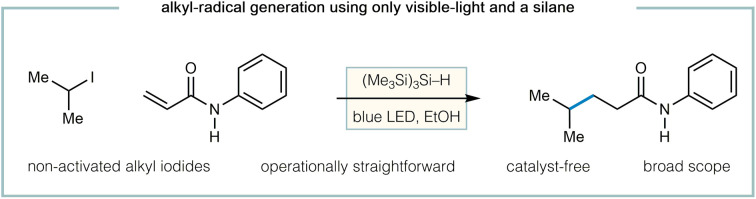

The efficient and straightforward construction of C(sp^3^)–C(sp^3^) bonds is a crucial process in organic synthesis. Over the past 80 years, the polar conjugate addition reaction has become a powerful method to forge a variety of C(sp^3^)–C(sp^3^) bonds.^1^ Alongside two-electron nucleophiles, alkyl-radicals – neutral yet nucleophilic species – have emerged as alternatives to organometallic reagents for additions to electron deficient alkenes.^2^ Since the 1960s, a variety of methods have been reported for the formation of alkyl-radicals; early examples include the decomposition of *in situ* generated organomercurial hydrides, the fragmentation of xanthate or Barton esters, or the UV-mediated homolysis of alkyl halides, amongst many others.^3^ Although these strategies tolerate a broad range of functionalities, the initiation processes can be complicated by the need for aggressive reaction conditions and frequently require toxic reagents such as tributyltin hydride, with notable exceptions.^4,5^

The emergence of photoredox catalysis has obviated many of the potential drawbacks to the generation and use of alkyl-radicals. The exploitation of the multifaceted reactivity of visible light excited transition metal or organic-photocatalysts, whose properties can be tuned through modification of the ligand, metal and/or scaffold, facilitates optimization of the single electron transfer event towards alkyl-radical generation from a wide range of functionalized alkyl groups.^6^ In addition, the reactivity of electron donor–acceptor (EDA) complexes has also provided a straightforward means to form alkyl-radicals from a variety of precursors.^7^ As such, a plethora of methods have been developed for the generation of C(sp^3^)-centred radicals from a variety of commercially available native functionalities, which dramatically expand the scope of alkyl-radical chemistry†. In this context, the single electron reduction of non-activated alkyl halides provides a useful means to generate alkyl radicals.^8^ As an example, Leonori and co-workers recently developed a method wherein halogen atom abstraction pathways were leveraged using radical species forged through photocatalyst-mediated oxidation event leading to a general alkyl-radical generation.^9^ Related to the current study, Jørgensen and co-workers published a visible-light mediated reduction of alkyl halides under very mild conditions. Accordingly, there remains a need for further innovation towards orthogonal, general and benign methods of alkyl-radical generation that tolerate a broad range of functionalities, thereby enabling the construction of a greater variety of C(sp^3^)–C(sp^3^) bonds.^10^

Recently, we reported a general reaction to form tertiary alkylamines *via* the addition of alkyl-radicals (generated from non-activated alkyl-iodides) to *in situ*-generated all-alkyl iminium ions.^11*a*^ This carbonyl alkylative amination (CAA) reaction was promoted by the action of blue LEDs and tris(trimethylsilyl)silane ((Me_3_Si)_3_Si–H). No photoredox catalyst is required. We believe that the alkyl-radical formation step, devoid of traditional initiating reagents, proceeds through the visible-light excitation of a transient ternary EDA complex, which stimulates homolysis of the carbon–iodide bond that would be otherwise stable under such irradiation conditions (Fig. 1B). The presence of an enamine was important to the initiation pathway, as revealed by an absorption band in the UV/vis spectrum of its mixture with an alkyl-iodide and (Me_3_Si)_3_Si–H.^11*a*^ Gouverneur and co-workers have also reported an elegant example of visible-light mediated addition of more functionalized alkyl halides, such as iodofluoromethane, to electron deficient alkenes.^12^ They proposed that light mediated homolytic cleavage of iodofluoromethane was responsible for radical initiation prior to a classical chain process.

**Fig. 1:**
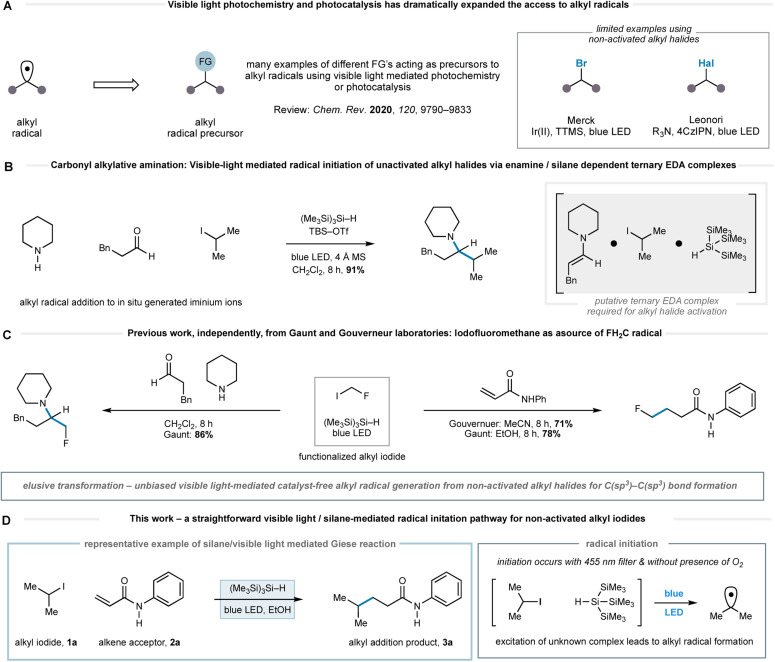
(A) Selected visible-light mediated methods for the generation of alkyl-radicals; (B) previous work – a method for tertiary amine formation exploiting a visible-light activation of a ternary EDA complex to promote alkyl-radical formation. (C) Previous work from Gouverneur & Gaunt labs on radical fluoromethylation. (D) This work – alkyl-radical formation promoted solely by visible light and tris-trimethylsilyl silane demonstrated through a remarkably practical and straightforward Giese reaction.

Gouverneur *et al.* also showed methyl iodide was only efficient as a radical source under these conditions when an organic photocatalyst was present and the reaction of other simple non-activated alkyl iodides was only demonstrated in the presence of iodofluoromethane, which was presumably responsible for the initiation pathway (*vide supra*). Our prior work in this area also identified iodofluoromethane as a visible-light activated source of fluoromethyl radical and its addition to iminium ions and electron deficient alkenes (Fig. 1C).^11*b*^ Taken together, these works reveal that the use of visible light and (Me_3_Si)_3_Si–H to initiate radical formation from non-activated alkyl halides has not been achieved in an unbiased transformation without the requirement of an initiation process *via* of the reaction components or a photocatalyst. Accordingly, we questioned whether a pathway mediated by visible-light and (Me_3_Si)_3_Si–H alone might facilitate alternative modes of radical initiation from non-activated alkyl halides, and therefore enable the general coupling of unbiased alkyl fragments with a wider range of acceptors under practical, straightforward reaction conditions.

Herein, we report the successful realization of this idea through the development of a remarkably straightforward visible-light mediated method for alkyl-radical generation from non-activated alkyl iodides using only non-toxic tris(trimethylsilyl)silane as a reagent (Fig. 1D). While we are not certain of the precise pathway for the radical initiation, it seems likely that excitation of a species resulting from the interaction of tris(trimethylsilyl)silane and the alkyl iodide, leading to carbon–iodide bond homolysis. The utility of this activation mode is demonstrated through a broad and chemoselective Giese addition to electron deficient alkenes and is notable by its tolerance to a range of synthetically valuable functionalities in both alkyl iodide and alkene components. In comparison to other methods for Giese-addition,^2,3,8,9,12^ the conditions are mild and do not require expensive catalysts or cocktails of additives.

Our studies were stimulated from an observation arising from the development of the visible light mediated carbonyl alkylative amination (shown in Fig. 1B). High yields of the tertiary amine product, arising from the union of alkyl-radical, aldehyde and secondary amine were maintained when using a 455 nm long-pass filter, which discounted UV-mediated carbon–iodide bond homolysis as the initiation pathway for alkyl-radical formation.^11*a*^ To explore the formation of an alkyl-radical independently from the enamine component, the reaction conditions were simplified to comprise a representative alkyl halide and (Me_3_Si)_3_Si–H, which allowed us to first assess any impact solvent might have on the radical forming process. As shown in Table 1, the visible-light/(Me_3_Si)_3_Si–H mediated hydrodehalogenation of adamantyl bromide 4 to adamantane 5 could be successfully assessed in a solvent-independent manner (Table 1) by recording the assay yield of the adamantane 5. Ether formation was not observed when using alcohols as solvent (entries 3, 4), confirming that cationic pathways were unlikely to be operative. Extending the reaction time to 16 h led to an excellent yield of 5 (entry 8). As observed with the carbonyl alkylative amination process, using a 455 nm long-pass filter did not affect the outcome (entry 9). Trace amounts of triplet oxygen have been widely proposed to generate silicon-centred radicals from (Me_3_Si)_3_Si–H.^13^ However, 47% of 5 was still obtained after visible-light irradiation of a reaction mixture from which air had been rigorously excluded (entry 10), suggesting an alternative initiation pathway excluding oxygen could also operate.^14^ A reaction at 80 °C in the absence of light showed no conversion to 5. This data shows the nature of the solvent is not relevant for the initiation step and suggests a straightforward radical initiation process that results from visible-light excitation of an intermediate arising from an interaction between the alkyl halide and (Me_3_Si)_3_Si–H.

**Table 1:** Effect of different parameters on radical initiation^a^

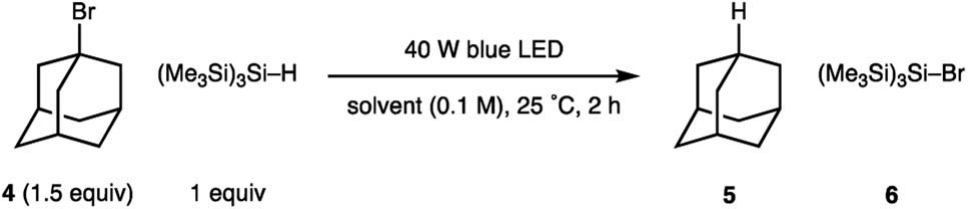			
Entry	Solvent	Deviation in conditions	Yield 5 (%)
1	CH_2_Cl_2_	—	33
2	THF	—	68
3	MeOH	—	85
4	EtOH	—	55
5	C_6_H_12_	—	84
6	PhH	—	41
7	PhMe	—	34
8	EtOH	16 h	86
9	EtOH	16 h, 455 nm filter	82
10	EtOH	16 h, degassed	47
11	EtOH	80 °C, dark	0

a Yields of 5 were calculated by ^1^H NMR using 1,1,2,2-tetrachloroethane as internal standard.

With the operationally simple and mild reaction conditions for the homolysis of non-activated alkyl halides, we next focussed on benchmarking the process against existing transformations: namely the Giese addition reaction of alkyl-radicals to electron deficient alkenes. Therefore, using acrylamide 2a (as a representative alkene acceptor), 3.0 equivalents of iso-propyl iodide 1a (as a representative non-activated alkyl halide) and 1.5 equivalents of (Me_3_Si)_3_Si–H in MeOH at 0.1 M, we were pleased to find visible light irradiation of this reaction mixture led to the formation of alkylamide 3a in 59% assay yield (Table 2, entry 1). A series of optimization experiments showed that the reaction could be improved to 66% assay yield by increasing the amount of (Me_3_Si)_3_Si–H to 2.0 equivalents alongside the concentration to 0.2 M (entry 2). Switching the solvent media to EtOH further increased the assay yield of 3a to 79% (entry 3). Reducing the equivalents of the alkyl-iodide did not significantly affect the assay yield of 3a (entry 4), although further reductions in the stoichiometry of alkyl halide or (Me_3_Si)_3_Si–H proved detrimental (entries 5, 6). As a result, optimal conditions required irradiation (40 W blue LED, Kessil lamp) of acrylamide 2a (1 equiv.), iso-propyl iodide 1a (2 equiv.), (Me_3_Si)_3_Si–H (1.5 equiv.) in a 0.2 M solution of anhydrous ethanol for 16 h (entry 4).

**Table 2:** Optimization of alkyl-radical addition to alkenes

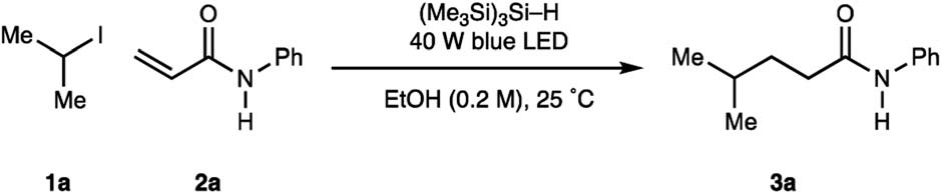					
Entry	Solvent	(Me_3_Si)_3_Si–H	Alkyl-iodide	Conc.	Yield 3a^a^ (%)
1	MeOH	1.5 equiv.	3.0 equiv.	0.1 M	59
2	MeOH	2.0 equiv.	3.0 equiv.	0.2 M	66
3	EtOH	2.0 equiv.	3.0 equiv.	0.2 M	79
4	EtOH	2.0 equiv.	2.0 equiv.	0.2 M	77
5	EtOH	2.0 equiv.	1.5 equiv.	0.2 M	70
6	EtOH	1.5 equiv.	1.5 equiv.	0.2 M	47

a Yields of 3a were calculated by ^1^H NMR using 1,1,2,2-tetrachloroethane as internal standard.

We next turned attention to evaluating the scope of the reaction (Table 3). As part of this study, we endeavoured to explore substrates displaying functionality that would be useful to the end user (for example, in a drug discovery setting) but potentially cause problems with reaction conditions operating through strongly oxidizing or reducing intermediates common in photoredox catalysis. Starting with an investigation of the alkene acceptor component, we found that wide range on *N*-aryl acrylamides were good substrates for the reaction under the catalyst-free conditions (3a–m). It was notable that the mild reaction conditions tolerated the presence of a wide range of functional groups comprising electron withdrawing motifs (3e, m), halogens (3b–c), unprotected phenols (3f, h, i), tertiary amines (3d) and boronic esters (3g), producing the respective products in synthetically useful to very good yields. Tertiary acrylamide derivatives (3j–k), including a substrate displaying a reductively sensitive N–O bond (3k), were also successfully converted to the desired products. Substitution at the β-position was tolerated with augmented electron-withdrawing capabilities of the maleimide motif and generated the corresponding substituted succinimide 3l in 90% yield. Substitution at the α-position did not affect the success of the reaction (3m). We were also pleased to see that a tertiary acrylamide displaying a complex quinazoline-derived substrate worked well in the reaction demonstrating the reactions capacity to operate in the presence of functionality frequently found in pharmaceutical candidates (3n). Beyond acrylamide substrates, acrylates (to 3o), acrylonitrile (to 3p), vinyl-phosphonates (to 3q), vinyl-sulfones (to 3r), and electron-deficient alkynes (to 3s) all underwent Giese addition using the catalyst-free protocol.

**Table 3:** Scope of catalyst-free Giese addition

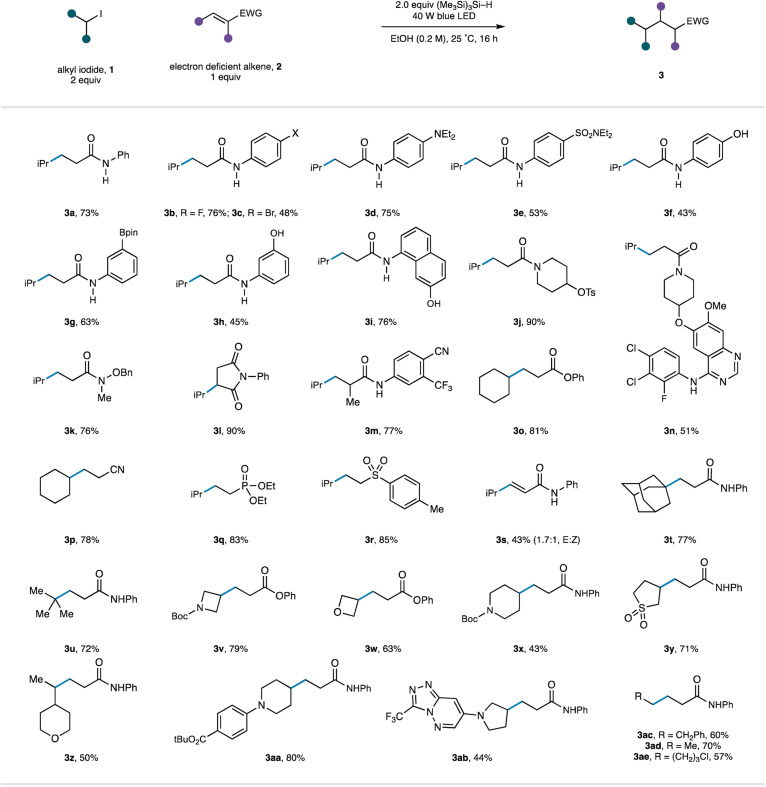

Next, the scope of the reaction in the alkyl halide component was investigated. Carbon-centred radicals formed through the visible-light/TTMS initiation pathway from simple tertiary alkyl-iodides (1-adamantyl to 3t and *tert*-butyl to 3u) underwent Giese addition with acrylamide 2a in excellent yields. A series of alkyl-iodides based on heterocyclic scaffolds were found to undergo smooth radical formation and Giese addition, providing products that can be further elaborated and could be utilized in the construction of pharmaceutically relevant molecules (3v–3ab). Finally, a selection of primary alkyl-iodides (3ac–ae) were found to be compatible with the radical activation mode and generated the linear alkyl products in good yields.

We had recognized a report by the Merck discovery group who showed that a visible-light mediated Ir-catalyzed Giese addition using alkyl bromides also utilized (Me_3_Si)_3_Si–H as a reagent to propagate alkyl-radical formation (Fig. 2A).^8*b*^ A distinct difference of our system is the departure from any requirement for a photocatalyst, which not only reduces the cost of the transformation but could offer an alternative protocol for Giese addition when redox sensitive groups appear in one of the coupling partners.

**Fig. 2:**
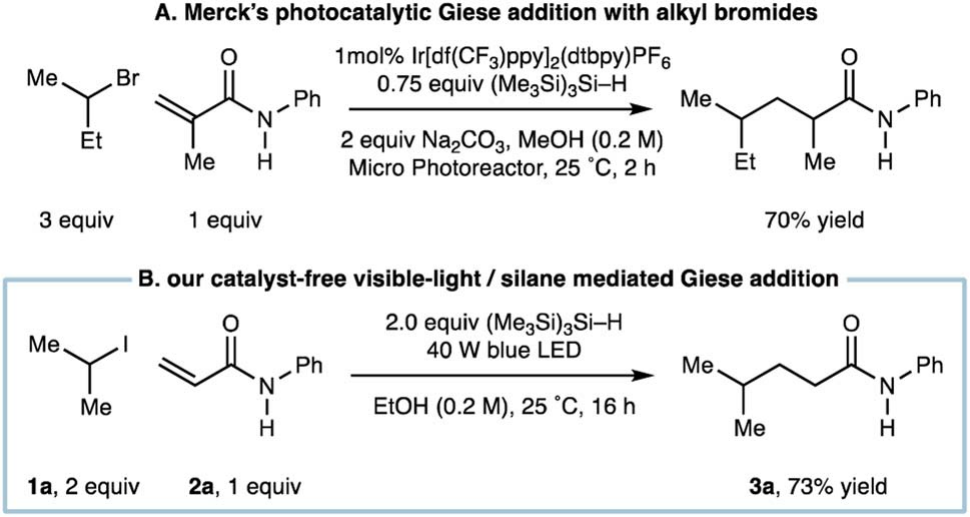
(A) Photocatalytic Giese addition with alkyl bromides mediated by (Me_3_Si)_3_Si–H; (B) catalyst-free visible-light/(Me_3_Si)_3_Si–H mediated Giese addition.

Several simple mechanistic experiments were conducted to supplement those shown in Table 1 and confirm the elementary steps in this Giese addition. A reaction using cyclopropylmethyl iodide 1q and acrylate 2o produced the corresponding cyclopentane 7 in 29% isolated yield (Fig. 3A), confirming the intermediacy of an alkyl radical. This result suggests a pathway involving β-scission of the cyclopropylmethyl radical (to int-I) prior to addition to the alkene (to int-II), which is followed by 5-exo trig cyclization (to int-III) and HAT of the resulting methyl radical with (Me_3_Si)_3_Si–H to form cyclopentane 7. A reaction conducted using d_5_-EtOD showed no incorporation of deuterium in the product, thereby eliminating solvent participation in the radical interception step (Fig. 3B), as expected from the observations outlined in Table 1. No reaction was observed in the absence of (Me_3_Si)_3_Si–H, highlighting the crucial role it plays in the overall transformation.

**Fig. 3:**
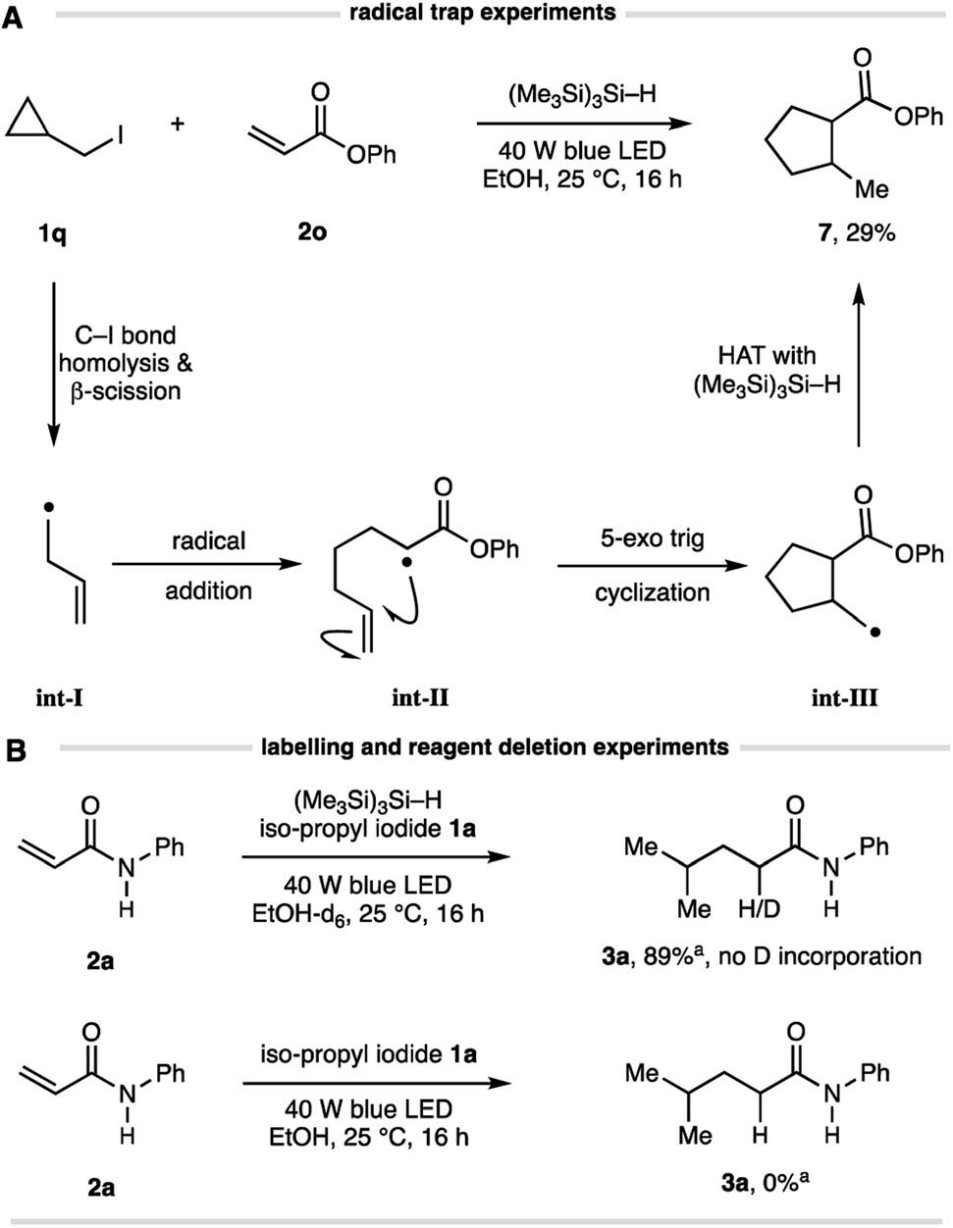
(A) Evidence for alkyl-radical formation *via* radical trap experiment; (B) experiments to demonstrate HAT is not from solvent and the essential role of the silane.

Despite the strength of our experimental observations, conclusive evidence that unravels the visible-light mediated radical initiation pathway between (Me_3_Si)_3_Si–H and the non-activated alkyl-iodide remains elusive. ^1^H NMR, UV-vis or IR spectroscopy titration studies aimed at identifying an interaction between a variety of alkyl-halides and (Me_3_Si)_3_Si–H could not be obtained (see ESI† for details). However, visible light must be exciting a transient intermediate comprising the silane and alkyl-iodide as the predominant pathway here because the process so efficiently homolyzes the carbon–iodide bond^15^ to yield alkyl-radicals. Consequently, we hypothesized that such an interaction may not be observable due to its transient nature and short lifetime or would be present in minute quantities beyond the detection limits of these spectroscopic methods. One possibility for the radical initiation is the excitation of a halogen bonded intermediate between the alkyl iodide and silane – whereby a coulombic attraction between a region of electron deficiency around the polarizable halogen atom (σ-hole)^16,17^ and the electron rich hydridic component of the silane – could result in weakening of the carbon–iodine bond, subsequently absorbing low energy visible light to stimulate homolysis (Fig. 1D). We do, however, acknowledge that an as yet undetermined pathway could be responsible for the radical initiation pathway. Despite the uncertainty over the mode of radical initiation, our understanding of the mechanism responsible for this catalyst-free Giese addition is detailed in Fig. 4. Visible-light and silane-mediated carbon–iodide bond homolysis, generates an alkyl-radical to initiate the process. Addition of the alkyl-radical to the alkene acceptor generates a new electrophilic radical, which undergoes HAT with (Me_3_Si)_3_Si–H to form the product. The resulting (Me_3_Si)_3_Si radical now undergoes halogen atom transfer (XAT) with a new molecule of the alkyl-iodide, thereby propagating the radical chain.

**Fig. 4:**
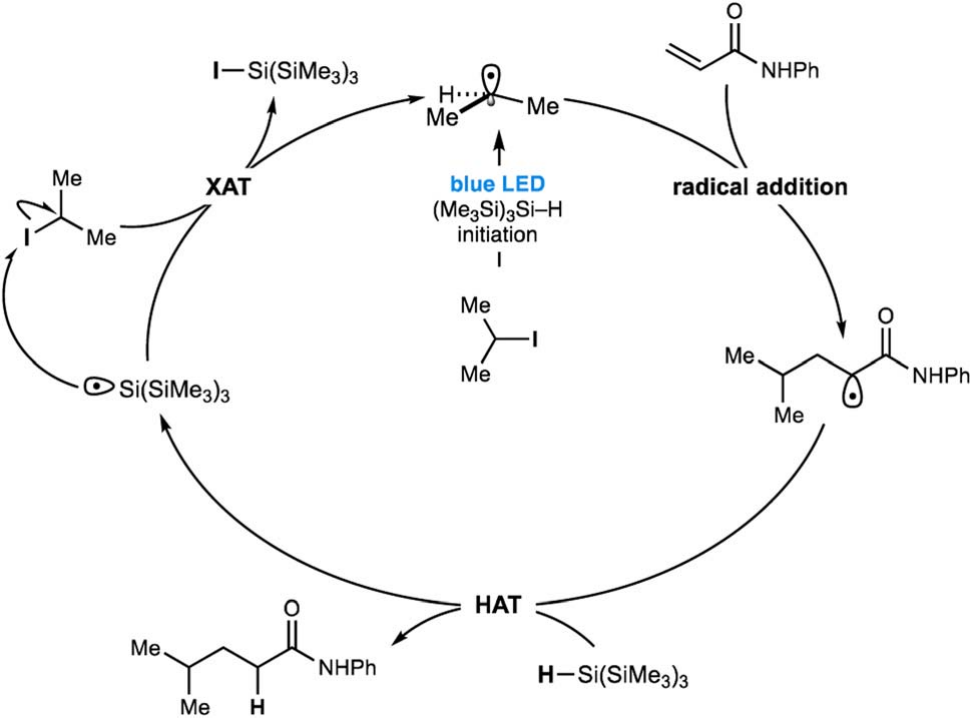
Current proposed mechanism for metal-free Giese addition.

In summary, we have developed an operationally straightforward method for alkyl-radical generation from non-activated alkyl-iodides and applied this protocol through the development of mild Giese addition. Through the action of visible light and (Me_3_Si)_3_Si–H, radical initiation from an alkyl-iodide is achieved under mild reaction conditions. No photocatalysts are required for this process. The range of alkyl-iodides and alkenes is very broad, and the reaction tolerates many sensitive functional groups. Considering current demand for saturated scaffolds in the drug discovery programs, we believe this strategy offers a potentially powerful method through which to combine two readily available classes of building blocks into complex molecules of biological and pharmaceutical interest. While the pathway of radical initation remains unclear, it is clear that the activation mode afforded by the combination of (Me_3_Si)_3_Si–H, alkyl iodide and visible-light provides a mild and general means for forming open shell alkyl intermediates.^11,12^ Subsequently, it is likely that this method will be useful to practitioners of synthetic chemistry in both academic and industrial settings.

## Data availability

Experimental procedures and characterization for all new compounds are available in the ESI.†

## Author contributions

R. K. and M. J. G. conceived the project. S. M. conducted the experiments. S. M., R. K., A. L. and M. J. G. analyzed the experiments. S. M., R. K., A. L. and M. J. G. wrote the manuscript.

## Conflicts of interest

There are no conflicts to declare.

## Supplementary Material

SC-013-D2SC03516B-s001

## References

[cit1] Csákÿ A. G., Herrán G. D. L., Murcia M. C. (2010). Chem. Soc. Rev..

[cit2] Giese B., Meister J. (1977). Angew. Chem., Int. Ed..

[cit3] Kuivila H. G. (1970). Synthesis.

[cit4] Suzuki A., Arase A., Matsumoto H., Itoh M., Brown H. C., Rogić M. M., Rathke M. W. A. (1967). J. Am. Chem. Soc..

[cit5] Zard S. Z. (2019). Helv. Chim. Acta.

[cit6] Narayanam J. M. R., Stephenson C. R. J. (2011). Chem. Soc. Rev..

[cit7] Silvi M., Verrier C., Rey Y. P., Buzzetti L., Melchiorre P. (2017). Nat. Chem..

[cit8] Nguyen J. D., D'Amato E. M., Narayanam J. M. R., Stephenson C. R. J. (2012). Nat. Chem..

[cit9] Constantin T., Zanini M., Regni A., Sheikh N. S., Juliá F., Leonori D. (2020). Science.

[cit10] Yan M., Lo J. C., Edwards J. T., Baran P. S. (2016). J. Am. Chem. Soc..

[cit11] Kumar R., Flodén N. J., Whitehurst W. G., Gaunt M. J. (2020). Nature.

[cit12] Hell S. M., Meyer C. F., Ortalli S., Sap J. B. I., Chen X., Gouverneur V. (2021). Chem. Sci..

[cit13] Chatgilialoglu C., Guerrini A., Seconi G., Guarini A. (1992). J. Org. Chem..

[cit14] Chatgilialoglu C., Ferreri C., Landais Y., Timokhin V. I. (2018). Chem. Rev..

[cit15] Luo Y.-R. , Comprehensive Handbook of Chemical Bond Energies, CRC Press, 1st edn, 2007, 10.1201/9781420007282

[cit16] Clark T., Hennemann M., Murray J. S., Politzer P. (2007). J. Mol. Model..

[cit17] For studies spectroscopic towards mechanism of radical initiation, see ESI,†, pp. 37–50

