# Supplementary material for: C(sp3)–C(sp3) coupling of non-activated alkyl-iodides with electron-deficient alkenes via visible-light/silane-mediated alkyl-radical formation
Source: Chem Sci. 2022 Oct 28;13(44):13241–7. doi: 10.1039/d2sc03516b (PMC9667957; doi:10.1039/d2sc03516b)
Supplement: SC-013-D2SC03516B-s001 [file SC-013-D2SC03516B-s001.pdf]

Electronic Supplementary Material (ESI) for Chemical Science.
This journal is © The Royal Society of Chemistry 2022

SUPPORTING INFORMATION

C(*sp*^{3})–C(*sp*^{3}) coupling of non-activated alkyl-iodides with electron-deficient alkenes via visible-light/silane-mediated alkyl-radical formation

Sanesh Mistry,1 Roopender Kumar,1 Andrew Lister2 and Matthew J. Gaunt1,*

Yusuf Hamied Department of Chemistry, University of Cambridge, Lensfield Road, Cambridge, CB2 1EW, United Kingdom.

*To whom correspondence should be addressed:
mjg32@cam.ac.uk

Contents

General Information 4

Abbreviations: 4

Experimental Procedures: 4

Materials: 4

Instrumentation: 4

General Procedure 6

4-Methyl-*N*-phenylpentanamide (**3a**) 6

N-(4-Flourophenyl)-4-methylpentanamide (**3b**) 7

N-(4-Bromophenyl)-4-methylpentanamide (**3c**) 8

N-(4-(Diethylamino)phenyl)-4-methylpentanamide (**3d**) 9

N-(4-(*N*,*N*-Dimethylsulfamoyl)phenyl)-4-methylpentanamide (**3e**) 10

N-(4-Hydroxyphenyl)-4-methylpentanamide (**3f**) 11

(3-(4-Methylpentanamido)phenyl)boronic acid pinacol ester (**3g**) 12

N-(3-Hydroxyphenyl)-4-methylpentanamide (**3h**) 13

N-(7-Hydroxynaphthalen-1-yl)-4-methylpentanamide (**3i**) 14

1-(4-Methylpentanoyl)piperidin-4-yl 4-methylbenzenesulfonate (**3j**) 15

N-(Benzyloxy)-*N*,4-dimethylpentanamide (**3k**) 16

3-Isopropyl-*N*-phenylsuccinimide (**3l**) 17

N-(4-Cyano-3-(trifluoromethyl)phenyl)-2,4-dimethylpentanamide (**3m**) 18

1-(4-((4-((3,4-Dichloro-2-fluorophenyl)amino)-7-methoxyquinazolin-6-yl)oxy)piperidin-1-yl)-4-methylpentan-1-one (**3n**) 19

Phenyl 3-cyclohexylpropanoate (**3o**) 20

3-Cyclohexylpropionitrile (**3p**) 20

Diethyl isopentylphosphonate (**3q**) 21

1-(Isopentylsulfonyl)-4-methylbenzene (**3r**) 22

4-Methyl-*N*-phenylpent-2-enamide (**3s**) 23

3-(Adamantan-1-yl)-*N*-phenylpropanamide (**3t**) 24

4,4-Dimethyl-*N*-phenylpentanamide (**3u**) 25

Tert-butyl 3-(3-oxo-3-phenoxypropyl)azetidine-1-carboxylate (**3v**) 26

Phenyl 3-(oxetan-3-yl)propanoate (**3w**) 27

Tert-butyl 4-(3-oxo-3-(phenylamino)propyl)piperidine-1-carboxylate (**3x**) 28

3-(1,1-Dioxidotetrahydrothiophen-3-yl)-*N*-phenylpropanamide (**3y**) 29

4-(Oxan-4-yl)-*N*-phenylpentanamide (**3z**) 30

Tert-butyl 4-(4-(3-oxo-3-(phenylamino)propyl)piperidin-1-yl)benzoate (**3aa**) 31

N-Phenyl-3-(1-(3-(trifluoromethyl)-[1,2,4]triazolo[4,3-*b*]pyridazin-6-yl)pyrrolidin-3-yl)propenamide (**3ab**) 32
N,5-Diphenylpentanamide (**3ac**) 33
N-Phenylpentanamide (**3ad**) 34
7-Chloro-*N*-phenylheptanamide (**3ae**) 35
Radical Clock Experiment 36
Phenyl 2-methylcyclopentane-1-carboxylate (**7**): 36
Halogen bond exploration 37
^{1}H NMR spectroscopy 37
UV-Visible titration studies 45
Flow-IR spectroscopy titration studies 49
Spectroscopic titration studies conclusion 50
References 51
^{1}H and ^{13}C Spectra 52
Radical Clock Experiment 84

General Information

Abbreviations:

PE refers to the fraction of petroleum ether which boils at 40-60 °C. TCE is 1,1,2,2-tetrachloroethane. TTMSS refers to tris(trimethylsilyl)silane. sFCC denotes silica flash column chromatography. Molecular sieves is abbreviated as MS.

Experimental Procedures:

Unless otherwise stated, all reactions were carried out in Biotage microwave vials equipped with magnetic stirrer bars and dried in an oven at 80 °C overnight. The vials were sealed with PTFE lined microwave caps. Standard Schlenk techniques were used to create an atmosphere of N_2. Irradiation was achieved with a 40 W Kessil A160WE LED – Tuna Blue lamps set to maximum blue and intensity settings. A desk fan was adopted to minimise heating to the greatest extent, through this was not necessary. Analytical TLC was run on Merck Millipore silica gel 60 F_{254} 0.2 mm plates. Visualisation was carried out using UV light at 254 nm, and then staining with a basic solution of $KMnO_4$. Purifications *via* sFCC were carried out using Material Harvest silica gel 60. Volatiles were removed *in vacuo* using a rotary evaporator at temperatures of 40 °C or below.

Materials:

Non-commercial reagents were generously provided by AstraZeneca. Anhydrous CH_2Cl_2 was distilled over CaH_2, then degassed by freeze-pump-thaw (three cycles) and stored over activated 4 Å MS under an atmosphere of N_2. However, similar results were obtained with anhydrous CH_2Cl_2 purchased from Acros Organics without further purification. All other reagents were purchased from commercially available suppliers and used as received. Anhydrous EtOH was bought from Sigma-Aldrich or Acros Organics, and anhydrous *t*-BuOH was supplied by Sigma-Aldrich. TTMSS, isopropyl iodide and *N*-phenylacrylamide were purchased from Fluorochem. Cyclohexyl iodide was purchased from Alfa Aesar, and phenyl acrylate was supplied by TCI.

Instrumentation:

^{1}H, ^{13}C, ^{11}B and ^{19}F NMR were recorded in $CDCl_3$ or MeOH-d_4 at room temperature on a Bruker DPX 500 spectrometer at 500 MHz, 126 MHz, 160 MHz and 471 MHz respectively, or using a Bruker DPX 400 spectrometer at 400 MHz, 101 MHz, 128 MHz and 376 MHz respectively. ^{31}P NMR were recorded in $CDCl_3$ at room temperature on a Bruker DPX 400 spectrometer at 162 MHz. Chemical

shifts (δ) are reported in parts per million (ppm) relative to residual $CHCl_3$ (δ_H = 7.26 and δ_C = 77.16) or residual MeOH-d_4 (δ_H = 3.31 and δ_C = 49.00). Coupling constants (J) are reported to the nearest 0.1 Hz. Multiplicities are reported as follows: s = singlet; d = doublet; t = triplet; q = quartet; m = multiplet; br. = broad; and associated combinations of therein, e.g. dd = doublet of doublets. Where similar coupling constants have been observed, the apparent (app.) multiplicity is reported. COSY, DEPT135, HSQC and HMBC experiments were used to aid assignment of structures, but are not included herein.

HRMS were recorded by the Mass Spectrometry section in the Department of Chemistry at the University of Cambridge using standard ESI techniques. IR spectra were run on a PerkinElmer FT-IR spectrometer fitted with an ATR sampler, either as neat or as a thin film applied using $CHCl_3$. Absorptions (ν_{max}) are reported in wavenumbers (cm^{-1}), with notable frequencies assigned. Where appropriate, broad (br.) signals are indicated.

General Procedure

To a microwave vial equipped with a stirrer bar and sealed with a septum cap, TTMSS (2.0-3.0 equiv.) was added to a solution of the acceptor (0.2-0.4 mmol, 1.00 equiv.) and alkyl iodide (2.0-3.0 equiv.) in anhydrous EtOH or *t*-BuOH (0.2 M) as specified under an atmosphere of N_2. The septum cap was reinforced with Parafilm, and the reaction was then irradiated utilising a 40 W Kessil A160WE LED (maximum intensity and maximum blue colour settings) for 16 h with maximum stirring. Upon completion, volatiles were removed *in vacuo* and the crude mixture purified by sFCC using the specified eluent. The relevant fractions were combined and concentrated *in vacuo* to afford the desired product.

4-Methyl-*N*-phenylpentanamide (**3a**)

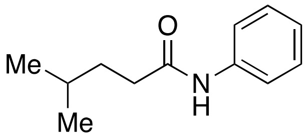

Prepared according to the general procedure using *N*-phenylacrylamide (60.0 mg, 0.4 mmol, 1.0 equiv.), anhydrous EtOH (2.0 mL), isopropyl iodide (80.0 µL, 0.8 mmol, 2.0 equiv.) and TTMSS (250 µL, 0.8 mmol, 2.0 equiv.). The crude product was purified by sFCC (0-100% EtOAc in heptane) to afford the title compound as a white solid (57.0 mg, 0.298 mmol, 73%).

^{1}H NMR (500 MHz, $CDCl_3$) δ 7.51 (d, *J* = 7.9 Hz, 2H), 7.32 (app. t, *J* = 7.9 Hz, 2H), 7.13-7.06 (m, 2H), 2.36 (t, *J* = 7.6 Hz, 2H), 1.67-1.60 (m, 3H), 0.94 (d, *J* = 5.9 Hz, 6H).
^{13}C NMR (126 MHz, $CDCl_3$) δ 171.8, 138.2, 129.1, 124.3, 120.0, 35.9, 34.6, 27.9, 22.5.
Data in agreement with those reported in the literature.[1]

N-(4-Flourophenyl)-4-methylpentanamide (**3b**)

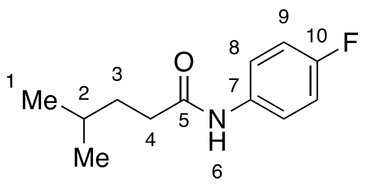

Prepared according to the general procedure using *N*-(4-fluorophenyl)acrylamide (33.7 mg, 0.2 mmol, 1.0 equiv.), anhydrous EtOH (1.0 mL), isopropyl iodide (60.0 µL, 0.6 mmol, 3.0 equiv.) and TTMSS (125 µL, 0.4 mmol, 2.0 equiv.). The crude product was purified by sFCC (0-100% EtOAc in heptane) to afford the title compound as a white solid (31.7 mg, 0.151 mmol, 76%).

^{1}H NMR (500 MHz, $CDCl_3$) δ 7.46 (dd, *J* = 8.8, 4.8 Hz, 2H, H-8), 7.06 (s, 1H, H-6), 7.01 (app. t, *J* = 8.7 Hz, 2H, H-9), 2.35 (t, *J* = 7.8 Hz, 2H, H-4), 1.70-1.58 (m, 3H, H-2, H-3), 0.94 (d, *J* = 5.9 Hz, 6H, H-1).
^{13}C NMR (126 MHz, $CDCl_3$) δ 171.8 (C-5), 159.4 (d, *J* = 243.4 Hz, C-10), 134.1 (d, *J* = 3.0 Hz, C-7), 121.9 (d, *J* = 7.6 Hz, C-8), 115.7 (d, *J* = 22.8 Hz, C-9), 35.8 (C-4), 34.5 (C-3), 27.9 (C-2), 22.4 (C-1).
^{19}F NMR (471 MHz, $CDCl_3$) δ –118.2- –118.3 (m, 1F, F-10).
HRMS calculated for $[C_{12}H_{17}FNO]^+$ ($[M + H]^+$) 210.1294, found 210.1286.
IR (neat) ν_{max}/cm^{-1} 3248 (br., N–H), 3072 (sp^2 C–H), 2957 (sp^3 C–H), 2913 (sp^3 C–H), 2869 (sp^3 C–H), 1651 (C=O), 1557, 1505, 1406, 1214 (C–F), 832 (sp^2 C–H), 795, 762, 523, 507.

N-(4-Bromophenyl)-4-methylpentanamide (**3c**)

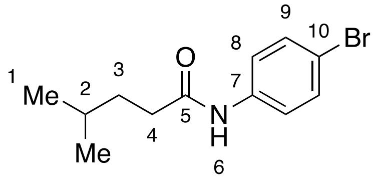

Prepared according to the general procedure using *N*-(4-bromophenyl)acrylamide (45.8 mg, 0.2 mmol, 1.0 equiv.), anhydrous EtOH (1.0 mL), isopropyl iodide (60.0 µL, 0.6 mmol, 3.0 equiv.) and TTMSS (125 µL, 0.4 mmol, 2.0 equiv.). The crude product was purified by sFCC (0-100% EtOAc in heptane) to afford the title compound as a white solid (26.0 mg, 9.62 x 10^{-2} mmol, 48%).

^{1}H NMR (500 MHz, $CDCl_3$) δ 7.43-7.39 (m, 4H, H-8, H-9), 7.06 (br. s, 1H, H-6), 2.35 (t, *J* = 7.6 Hz, 2H, H-4), 1.71-1.58 (m, 3H, H-2, H-3), 0.94 (d, *J* = 6.0 Hz, 6H, H-1).

^{13}C NMR (126 MHz, $CDCl_3$) δ 171.9 (C-5), 137.2 (C-7), 132.0 (C-8 or C-9), 121.5 (C-8 or C-9), 116.8 (C-10), 35.9 (C-4), 34.5 (C-3), 27.9 (C-2), 22.4 (C-1).

HRMS calculated for $[C_{12}H_{17}{}^{79}BrNO]^+$ ($[M + H]^+$) 270.0494, found 270.0502 and 272.0487 for $[C_{12}H_{17}{}^{81}BrNO]^+$ ($[M + H]^+$).

IR (neat) ν_{max}/cm^{-1} 3308 (br., N–H), 2958 (sp^2 C–H), 2926 (sp^3 C–H), 2868 (sp^3 C–H), 1659 (C=O), 1519, 1488, 1392 (sp^3 C–H), 1072, 817 (sp^2 C–H), 694 (C–Br), 503.

N-(4-(Diethylamino)phenyl)-4-methylpentanamide (**3d**)

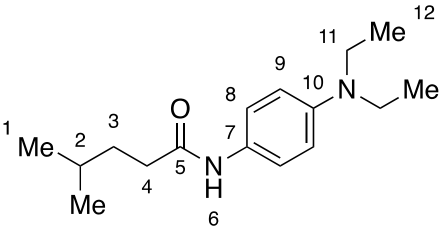

Prepared according to the general procedure using *N*-(4-(diethylamino)phenyl)acrylamide (87.4 mg, 0.4 mmol, 1.0 equiv.), anhydrous EtOH (2.0 mL), isopropyl iodide (80.0 µL, 0.8 mmol, 2.0 equiv.) and TTMSS (250 µL, 0.8 mmol, 2.0 equiv.). The crude product was purified by sFCC (20-40% EtOAc in PE, containing 0.1 v/v% Et_3N) to afford the title compound as an off-white solid (79.1 mg, 0.301 mmol, 75%).

R_f 0.23 (30% EtOAc in PE, containing 0.1 v/v% Et_3N).

^{1}H NMR (400 MHz, $CDCl_3$) δ 7.40 (br. s, 1H, H-6), 7.30 (d, *J* = 8.1 Hz, 2H, H-9), 6.63 (d, *J* = 8.0 Hz, 2H, H-8), 3.31 (q, *J* = 6.7 Hz, 4H, H-11), 2.31 (t, *J* = 8.1 Hz, 2H, H-4), 1.66-1.56 (m, 3H, H-2, H-3), 1.13 (t, *J* = 6.7 Hz, 6H, H-12), 0.94 (d, *J* = 6.0 Hz, 6H, H-1).

^{13}C NMR (101 MHz, $CDCl_3$) δ 171.6 (C-5), 145.0 (C-10), 127.2 (C-7), 122.4 (C-9), 112.7 (C-8), 45.1 (C-11), 36.0 (C-4), 35.1 (C-3), 28.2 (C-2), 22.8 (C-1), 12.7 (C-12).

HRMS calculated for $[C_{16}H_{27}N_2O]^+$ ($[M + H]^+$) 263.2118, found 263.2111.

IR (neat) ν_{max}/cm^{-1} 3286 (br., N−H), 2961 (sp^2 C−H), 2925, 2869 (sp^3 C−H), 1647 (C=O), 1514, 1262 (sp^2 C−N).

N-(4-(*N*,*N*-Dimethylsulfamoyl)phenyl)-4-methylpentanamide (**3e**)

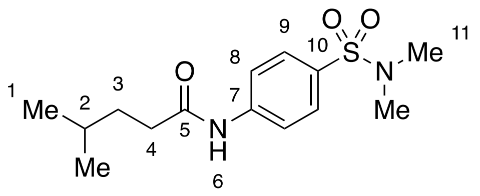

Prepared according to the general procedure using *N*-(4-(*N,N*-dimethylsulfamoyl)phenyl)acrylamide (103 mg, 0.4 mmol, 1.0 equiv.), anhydrous EtOH (2.0 mL), isopropyl iodide (80.0 µL, 0.8 mmol, 2.0 equiv.) and TTMSS (250 µL, 0.8 mmol, 2.0 equiv.). The crude product was purified by sFCC (40-60% EtOAc in PE) to afford the title compound as a white solid (62.6 mg, 0.210 mmol, 53%).

R_f 0.17 (40% EtOAc in PE).

^{1}H NMR (400 MHz, $CDCl_3$) δ 7.74-7.67 (m, 4H, H-8, H-9), 7.40 (br. s, 1H, H-6), 2.68 (s, 6H, H-11), 2.40 (t, *J* = 7.6 Hz, 2H, H-4), 1.67-1.60 (m, 3H, H-2, H-3), 0.94 (d, *J* = 6.1 Hz, 6H, H-1).

^{13}C NMR (101 MHz, $CDCl_3$) δ 172.4 (C-5), 142.5 (C-7), 130.0 (C-10), 129.1 (C-8), 119.5 (C-9), 38.2 (C-11), 36.0 (C-4), 34.3 (C-3), 27.9 (C-2), 22.4 (C-1).

HRMS calculated for $[C_{14}H_{22}N_2NaO_3S]^+$ ($[M + Na]^+$) 321.1243, found 321.1231.

IR (neat) ν_{max}/cm^{-1} 3303 (br., N–H), 3107 (sp^2 C–H), 2954, 2905 (sp^3 C–H), 1667 (C=O), 1589, 1534, 1400, 1335 (S=O), 1309 (sp^2 C–N), 1145 (S=O), 1104, 949, 842 (sp^2 C–H), 724, 705.

N-(4-Hydroxyphenyl)-4-methylpentanamide (**3f**)

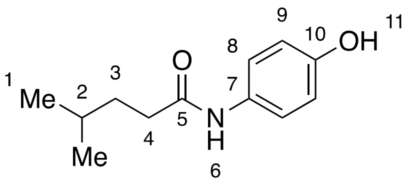

Prepared according to the general procedure using *N*-(4-hydroxyphenyl)acrylamide (33.7 mg, 0.2 mmol, 1.0 equiv.), anhydrous EtOH (1.0 mL), isopropyl iodide (60.0 µL, 0.6 mmol, 3.0 equiv.) and TTMSS (125 µL, 0.4 mmol, 2.0 equiv.). The crude product was purified by sFCC (0-100% EtOAc in heptane) to afford the title compound as a white solid (17.7 mg, 8.54 x 10^{-2} mmol, 43%).

^{1}H NMR (500 MHz, $CDCl_3$) δ 7.34 (d, *J* = 8.8 Hz, 2H, H-9), 7.00 (br. s, 1H, H-6), 6.78 (d, *J* = 8.8 Hz, 2H, H-8), 4.87 (s, 1H, H-11), 2.34 (t, *J* = 7.6 Hz, 2H, H-4), 1.69-1.58 (m, 3H, H-2, H-3), 0.94 (d, *J* = 6.1 Hz, 6H, H-1).

^{13}C NMR (126 MHz, $CDCl_3$) δ 172.2 (C-5), 153.3 (C-10), 130.4 (C-7), 122.8 (C-9), 115.9 (C-8), 35.7 (C-4), 34.7 (C-3), 28.0 (C-2), 22.5 (C-1).

HRMS calculated for $[C_{12}H_{18}NO_2]^+$ ($[M + H]^+$) 208.1338, found 208.1336.

IR (neat) ν_{max}/cm^{-1} 3312 (br., O–H), 2955 (sp^2 C–H), 2923 (sp^3 C–H), 2870 (sp^3 C–H), 1652 (C=O), 1532, 1512, 1378 (O–H), 1229, 1106, 830 (sp^2 C–H), 794, 670, 514.

(3-(4-Methylpentanamido)phenyl)boronic acid pinacol ester (**3g**)

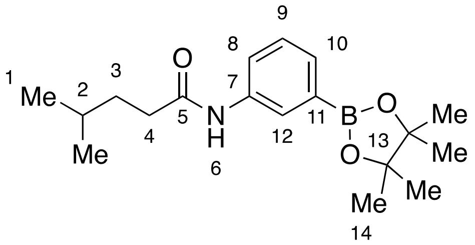

Prepared according to the general procedure using (3-acrylamidophenyl)boronic acid pinacol ester (111 mg, 0.4 mmol, 1.0 equiv.), anhydrous EtOH (2.0 mL), isopropyl iodide (80.0 µL, 0.8 mmol, 2.0 equiv.) and TTMSS (250 µL, 0.8 mmol, 2.0 equiv.). The crude product was purified by sFCC (0-100% EtOAc in heptane) to afford the title compound as a white solid (80.2 mg, 0.253 mmol, 63%).

^{1}H NMR (500 MHz, $CDCl_3$) δ 7.95-7.89 (m, 1H, H-8), 7.62 (d, J = 1.5 Hz, 1H, H-12), 7.53 (d, J = 7.4 Hz, 1H, H-10), 7.34 (app. t, J = 7.4 Hz, 1H, H-9), 7.08 (br. s, 1H, H-6), 2.34 (t, J = 7.8 Hz, 2H, H-4), 1.69-1.58 (m, 3H, H-2, H-3), 1.34 (s, 12H, H-14), 0.94 (d, J = 6.2 Hz, 6H, H-1).
^{13}C NMR (126 MHz, $CDCl_3$) δ 171.9 (C-5), 137.7 (C-7), 130.5 (C-10), 129.7 (br., C-11), 128.5 (C-9), 126.0 (C-12), 123.2 (C-8), 83.9 (C-13), 35.8 (C-4), 34.5 (C-3), 27.8 (C-2), 24.9 (C-14), 22.4 (C-1).
^{11}B NMR (160 MHz, $CDCl_3$) δ 31.1 (B-11).
HRMS calculated for $[C_{18}H_{29}BNO_3]^+$ ($[M + H]^+$) 318.2240, found 318.2247.
IR (neat) ν_{max}/cm^{-1} 3281 (br., N–H), 2956 (sp^2 C–H), 2927 (sp^3 C–H), 2870 (sp^3 C–H), 1656 (C=O), 1543, 1424, 1354, 1141 (C–O), 852 (sp^2 C–H), 705 (sp^2 C–H).

N-(3-Hydroxyphenyl)-4-methylpentanamide (**3h**)

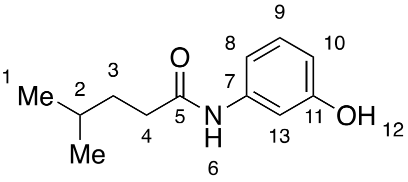

Prepared according to the general procedure using *N*-(3-hydroxyphenyl)acrylamide (66.4 mg, 0.4 mmol, 1.0 equiv.), anhydrous EtOH (2.0 mL), isopropyl iodide (80.0 µL, 0.8 mmol, 2.0 equiv.) and TTMSS (250 µL, 0.8 mmol, 2.0 equiv.). The crude product was purified by sFCC (0-100% EtOAc in heptane) to afford the title compound as a white solid (37.3 mg, 0.180 mmol, 45%).

^{1}H NMR (500 MHz, $CDCl_3$) δ 7.62 (app. t, *J* = 2.3 Hz, 1H, H-13), 7.17-7.10 (m, 2H, H-6, H-9), 6.73-6.67 (m, 2H, H-10, H-12), 6.61 (ddd, *J* = 8.2, 2.5, 0.6 Hz, 1H, H-8), 2.37 (t, *J* = 7.8 Hz, 2H, H-4), 1.70-1.59 (m, 3H, H-2, H-3), 0.94 (d, *J* = 6.1 Hz, 6H, H-1).

^{13}C NMR (126 MHz, $CDCl_3$) *δ* 172.8 (C-5), 157.7 (C-11), 138.8 (C-7), 129.8 (C-9), 112.0 (C-10), 110.6 (C-8), 107.5 (C-13), 36.3 (C-4), 34.6 (C-3), 27.9 (C-2), 22.4 (C-1).

HRMS calculated for $[C_{12}H_{18}NO_2]^+$ ($[M + H]^+$) 208.1338, found 208.1333.

IR (neat) ν_{max}/cm^{-1} 3292 (br., N–H), 3095 (br., O–H), 2957 (sp^2 C–H), 2919 (sp^3 C–H), 2868 (sp^3 C–H), 1649 (C=O), 1603 (N–H), 1547, 1444, 1271 (C–N), 1258, 872 (sp^2 C–H), 770 (sp^2 C–H), 688 (sp^2 C–H).

N-(7-Hydroxynaphthalen-1-yl)-4-methylpentanamide (**3i**)

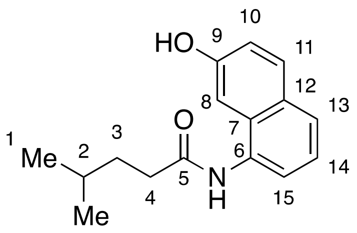

Prepared according to the general procedure using *N*-(7-hydroxynaphthalen-1-yl)acrylamide (44.8 mg, 0.2 mmol, 1.0 equiv.), anhydrous EtOH (1.0 mL), isopropyl iodide (60.0 µL, 0.6 mmol, 3.0 equiv.) and TTMSS (125 µL, 0.4 mmol, 2.0 equiv.). The crude product was purified by sFCC (0-100% EtOAc in heptane) to afford the title compound as a yellow solid (39.2 mg, 0.152 mmol, 76%).

^{1}H NMR (400 MHz, MeOH-d$_4$) δ 7.74 (d, *J* = 8.9 Hz, 1H, H-11), 7.66 (d, *J* = 8.1 Hz, 1H, H-13), 7.39 (d, *J* = 7.7 Hz, 1H, H-15), 7.24 (app. t, *J* = 7.8 Hz, 1H, H-14), 7.21 (d, *J* = 2.3 Hz, 1H, H-8), 7.10 (dd, *J* = 8.9, 2.4 Hz, 1H, H-10), 2.53 (t, *J* = 7.6 Hz, 2H, H-4), 1.76-1.65 (m, 3H, H-2, H-3), 1.01 (d, *J* = 6.1 Hz, 6H, H-1).

^{13}C NMR (101 MHz, MeOH-d$_4$) δ 176.3 (C-5), 157.1 (C-9), 132.6 (C-6), 132.2 (C-7), 131.0 (C-11), 130.7 (C-12), 127.7 (C-13), 125.0 (C-15), 123.3 (C-14), 119.5 (C-10), 105.2 (C-8), 36.1 (C-3), 35.6 (C-4), 29.2 (C-2), 23.0 (C-1).

HRMS calculated for $[C_{16}H_{18}NO_2]^-$ ($[M - H]^-$) 256.1338, found 256.1331.

IR (neat) ν_{max}/cm^{-1} 3260 (br., O−H), 2955 (sp^2 C−H), 2924 (sp^3 C−H), 1631 (C=O), 1601, 1507, 1446 (sp^3 C−H), 1259 (C−N), 1230, 1214, 746, 717 (sp^2 C−H).

1-(4-Methylpentanoyl)piperidin-4-yl 4-methylbenzenesulfonate (**3j**)

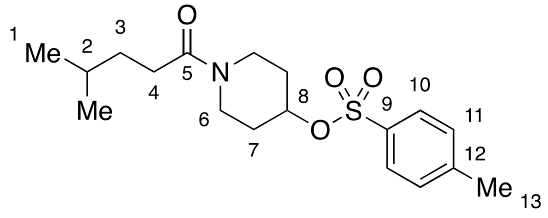

Prepared according to the general procedure using 1-acryloylpiperidin-4-yl 4-methylbenzenesulfonate (62.2 mg, 0.2 mmol, 1.0 equiv.), anhydrous EtOH (1.0 mL), isopropyl iodide (60.0 µL, 0.6 mmol, 3.0 equiv.) and TTMSS (185 µL, 0.6 mmol, 3.0 equiv.). The crude product was purified by sFCC (30-50% EtOAc in PE) to afford the title compound as a pale yellow oil (63.7 mg, 0.180 mmol, 90%).

$\mathbf{R}_{\boldsymbol{f}}$ 0.20 (40% EtOAc in PE).

^{1}H NMR (400 MHz, $CDCl_3$) δ 7.79 (d, J = 8.4 Hz, 2H, H-10), 7.34 (d, J = 8.1 Hz, 2H, H-11), 4.75-4.68 (m, 1H, H-8), 3.72-3.55 (m, 2H, H-6, H-6’), 3.54-3.45 (m, 1H, H-6), 3.38-3.29 (m, 1H, H-6’), 2.44 (s, 3H, H-13), 2.31-2.24 (m, 2H, H-4), 1.91-1.43 (m, 7H, H-2, H-3, H-7), 0.88 (d, J = 6.4 Hz, 6H, H-1).

^{13}C NMR (101 MHz, $CDCl_3$) δ 172.0 (C-5), 145.1 (C-12), 134.5 (C-9), 130.2 (C-11), 127.8 (C-10), 77.5 (C-8), 42.2 (C-6’), 38.1 (C-6), 34.3 (C-3), 32.2 (C-7), 31.4 (C-4), 31.1 (C-7’), 28.0 (C-2), 22.5 (C-1), 21.8 (C-13).

HRMS calculated for $[C_{18}H_{27}NNaO_4S]^+$ ($[M + Na]^+$) 376.1553, found 376.1556.

IR (film) ν_{max}/cm^{-1} 2955 (sp^2 C–H), 2869 (sp^3 C–H), 1642 (C=O), 1438, 1355 (S=O), 1188 (S=O), 1175 (C–O), 1010, 944, 908, 851 (sp^2 C–H), 815, 670.

N-(Benzyloxy)-*N*,4-dimethylpentanamide (**3k**)

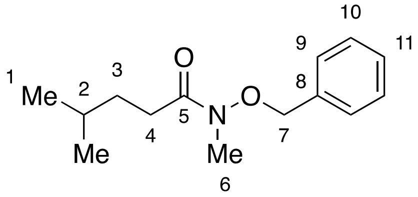

Prepared according to the general procedure using *N*-(benzyloxy)-*N*-methylacrylamide (38.9 mg, 0.2 mmol, 1.0 equiv.), anhydrous *t*-BuOH (1.0 mL), isopropyl iodide (60.0 µL, 0.6 mmol, 3.0 equiv.) and TTMSS (125 µL, 0.4 mmol, 2.0 equiv.). The crude product was purified by sFCC (10-30% EtOAc in PE) to afford the title compound as a yellow oil (35.8 mg, 0.152 mmol, 76%).

R_f 0.22 (20% EtOAc in PE).

^{1}H NMR (400 MHz, $CDCl_3$) δ 7.43-7.34 (m, 5H, H-9, H-10, H-11), 4.83 (s, 2H, H-7), 3.20 (s, 3H, H-6), 2.36 (t, *J* = 7.8 Hz, 2H, H-4), 1.60-1.43 (m, 3H, H-2, H-3), 0.87 (d, *J* = 6.4 Hz, 6H, H-1).

^{13}C NMR (101 MHz, $CDCl_3$) δ 175.9 (C-5), 134.7 (C-8), 129.4 (C-9), 129.1 (C-10 or C-11), 128.8 (C-10 or C-11), 76.4 (C-7), 33.7 (C-6), 33.5 (C-3), 30.4 (C-4), 28.0 (C-2), 22.5 (C-1).

HRMS calculated for $[C_{14}H_{22}NO_2]^+$ ($[M + H]^+$) 236.1651, found 236.1640.

IR (film) ν_{max}/cm^{-1} 2955 (sp^2 C–H), 2872 (sp^3 C–H), 1662 (C=O), 1455, 1414, 1384 (N–O), 1173 (C–O), 1080, 988, 909, 749, 699 (sp^2 C–H).

3-Isopropyl-*N*-phenylsuccinimide (**3l**)

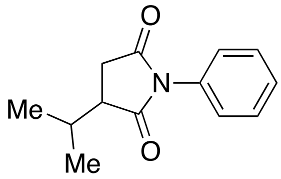

Prepared according to the general procedure using *N*-phenylmaleimide (70.1 mg, 0.4 mmol, 1.0 equiv.), anhydrous EtOH (2.0 mL), isopropyl iodide (80.0 µL, 0.8 mmol, 2.0 equiv.) and TTMSS (250 µL, 0.8 mmol, 2.0 equiv.). The crude product was purified by sFCC (10-30% EtOAc in PE) to afford the title compound as a white solid (78.3 mg, 0.360 mmol, 90%).

^{1}H NMR (500 MHz, $CDCl_3$) δ 7.49-7.44 (m, 2H), 7.39 (tt, *J* = 7.4, 1.3 Hz, 1H), 7.28-7.25 (m, 2H), 3.00-2.94 (m, 1H), 2.86 (dd, *J* = 18.4, 9.4 Hz, 1H), 2.63 (dd, *J* = 18.4, 4.4 Hz, 1H), 2.47-2.39 (m, 1H), 1.07 (d, *J* = 6.9 Hz, 3H), 0.99 (d, *J* = 6.8 Hz, 3H).

^{13}C NMR (126 MHz, $CDCl_3$) δ 178.5, 176.0, 132.1, 129.3, 128.7, 126.6, 45.9, 30.7, 29.3, 20.1, 17.4.

Data in agreement with those reported in the literature.[2]

N-(4-Cyano-3-(trifluoromethyl)phenyl)-2,4-dimethylpentanamide (**3m**)

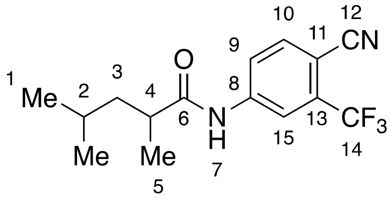

Prepared according to the general procedure using *N*-(4-cyano-3-(trifluoromethyl)phenyl)methacrylamide (49.2 mg, 0.2 mmol, 1.0 equiv.), anhydrous EtOH (1.0 mL), isopropyl iodide (60.0 µL, 0.6 mmol, 3.0 equiv.) and TTMSS (125 µL, 0.4 mmol, 2.0 equiv.). The crude product was purified by sFCC (10-30% EtOAc in PE) to afford the title compound as a pale yellow oil (44.0 mg, 0.155 mmol, 77%).

R*f* 0.18 (20% EtOAc in PE).

^{1}H NMR (500 MHz, $CDCl_3$) *δ* 8.01 (d, *J* = 2.2 Hz, 1H, H-15), 7.93 (dd, *J* = 8.6, 2.2 Hz, 1H, H-9), 7.78 (d, *J* = 8.5 Hz, 1H, H-10), 7.42 (br. s, 1H, H-7), 2.51-2.43 (m, 1H, H-4), 1.73-1.60 (m, 2H, H-2, H-3), 1.38-1.31 (m, 1H, H-3'), 1.25 (d, *J* = 6.9 Hz, 3H, H-5), 0.95 (d, *J* = 6.5 Hz, 3H, H-1), 0.92 (d, *J* = 6.4 Hz, 3H, H-1').

^{13}C NMR (126 MHz, $CDCl_3$) *δ* 176.3 (C-6), 142.8 (C-8), 135.9 (C-10), 134.1 (q, *J* = 32.8 Hz, C-13), 122.3 (q, *J* = 274.2 Hz, C-14), 121.9 (C-9), 117.4 (q, *J* = 5.0 Hz, C-15), 115.9 (C-12), 103.8 (q, *J* = 1.8 Hz, C-11), 43.3 (C-3), 40.7 (C-4), 26.0 (C-2), 22.7 (C-1), 22.6 (C-1'), 18.1 (C-5).

^{19}F NMR (471 MHz, $CDCl_3$) *δ* –62.2 (s, 3F, F-14).

HRMS calculated for $[C_{15}H_{18}F_3N_2O]^+$ ($[M + H]^+$) 299.1371, found 299.1368.

IR (film) ν_{max}/cm^{-1} 3273 (br., N–H), 2957 (sp^2 C–H), 2916 (sp^2 C–H), 2872 (sp^3 C–H), 2232 (sp C–N), 1667 (C=O), 1585 (N–H), 1520, 1426, 1325 (sp^2 C–N), 1242, 1172, 1134 (C–F), 1051, 848 (sp^2 C–H), 675, 558.

1-(4-((4-((3,4-Dichloro-2-fluorophenyl)amino)-7-methoxyquinazolin-6-yl)oxy)piperidin-1-yl)-4-methylpentan-1-one (**3n**)

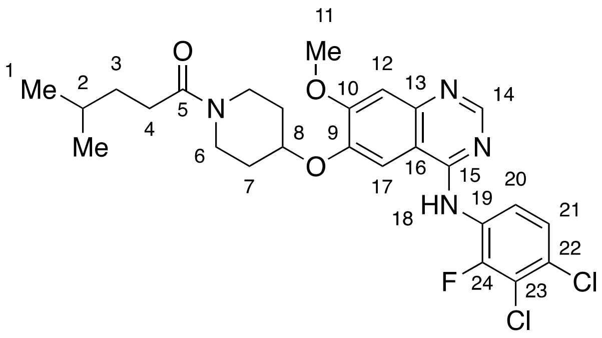

Prepared according to the general procedure using poziotinib (98.4 mg, 0.2 mmol, 1.0 equiv.), anhydrous EtOH (1.0 mL), isopropyl iodide (60.0 µL, 0.6 mmol, 3.0 equiv.) and TTMSS (125 µL, 0.4 mmol, 2.0 equiv.). The crude product was purified by sFCC (1-3% MeOH in CH_2Cl_2) to afford the title compound as an off-white solid (54.3 mg, 0.101 mmol, 51%).

R*f* 0.38 (6% MeOH in CH_2Cl_2).

^{1}H NMR (400 MHz, $CDCl_3$) *δ* 8.63 (s, 1H, H-14), 8.21-8.09 (m, 1H, H-21), 8.02 (br. s, 1H, H-18), 7.44 (s, 1H, H-17), 7.32-7.27 (m, 2H, H-12, H-20), 4.69-4.58 (m, 1H, H-8), 3.96 (s, 3H, H-11), 3.92-3.83 (m, 1H, H-6), 3.81-3.71 (m, 1H, H-6’), 3.54-3.43 (m, 1H, H-6), 3.41-3.31 (m, 1H, H-6’), 2.36-2.28 (m, 2H, H-4), 2.04-1.74 (m, 4H, H-7), 1.60-1.40 (m, 3H, H-2, H-3), 0.87 (d, *J* = 6.5 Hz, 6H, H-1).

^{13}C NMR (101 MHz, $CDCl_3$) *δ* 172.5 (C-5), 156.8 (C-10), 156.3 (C-15), 153.9 (C-14), 151.2 (d, *J* = 248.4 Hz, C-24), 148.3 (C-13), 147.2 (C-9), 128.7 (C-22), 127.0 (d, *J* = 10.8 Hz, C-23), 125.2 (d, *J* = 4.1 Hz, C-20), 123.1 (C-21), 120.7 (d, *J* = 18.7 Hz, C-19), 109.6 (C-16), 108.6 (C-12), 106.3 (C-17), 74.5 (C-8), 56.5 (C-11), 42.8 (C-6’), 38.8 (C-6), 34.5 (C-3), 31.6 (C-4), 31.4 (C-7), 30.2 (C-7’), 28.2 (C-2), 22.6 (C-1).

^{19}F NMR (376 MHz, $CDCl_3$) *δ* –125.5-126.1 (m, 1F, F-24).

HRMS calculated for $[C_{26}H_{30}{}^{35}Cl_2FN_4O_3]^+$ ($[M + H]^+$) 535.1674, found 535.1654 and 537.1622 for $[C_{26}H_{30}{}^{35}Cl^{37}ClFN_4O_3]^+$ ($[M + H]^+$) and 539.1591 for $[C_{26}H_{30}{}^{37}Cl_2FN_4O_3]^+$ ($[M + H]^+$).

IR (neat) ν_{max}/cm^{-1} 3241 (br., N–H), 2954 (sp^2 C–H), 2924, 2867 (sp^3 C–H), 1618 (C=O), 1589, 1496, 1469 (sp^3 C–H), 1417, 1385 (sp^3 C–H), 1231 (C–O), 1207 (C–F), 1010 (sp^3 C–N), 881, 851 (C–Cl).

Phenyl 3-cyclohexylpropanoate (**3o**)

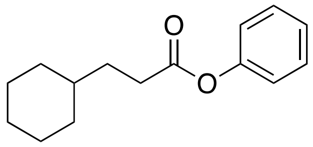

Prepared according to the general procedure using phenyl acrylate (56.0 µL, 0.4 mmol, 1.0 equiv.), anhydrous *t*-BuOH (2.0 mL), cyclohexyl iodide (104 µL, 0.8 mmol, 2.0 equiv.) and TTMSS (250 µL, 0.8 mmol, 2.0 equiv.). The crude product was purified by sFCC (10-11% CH_2Cl_2 in PE) to afford the title compound as a yellow oil (75.0 mg, 0.323 mmol, 81%).

^{1}H NMR (400 MHz, $CDCl_3$) δ 7.37 (app. t, *J* = 7.8 Hz, 2H), 7.22 (tt, *J* = 7.5, 1.1 Hz, 1H), 7.07 (dd, *J* = 7.6, 1.2 Hz, 2H), 2.57 (t, *J* = 8.0 Hz, 2H), 1.81-1.62 (m, 7H), 1.39-1.10 (m, 4H), 0.95 (m, 2H).
^{13}C NMR (101 MHz, $CDCl_3$) δ 172.7, 150.9, 129.5, 125.8, 121.8, 37.4, 33.1, 32.4, 32.2, 26.7, 26.4.
Data in agreement with those reported in the literature.[3]

3-Cyclohexylpropionitrile (**3p**)

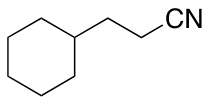

Prepared according to the general procedure using acrylonitrile (26.0 µL, 0.4 mmol, 1.0 equiv.), anhydrous EtOH (2.0 mL), cyclohexyl iodide (105 µL, 0.8 mmol, 2.0 equiv.) and TTMSS (250 µL, 0.8 mmol, 2.0 equiv.). The crude product was purified by sFCC (0-2% Et_2O in PE) to afford the title compound as a yellow oil (78%). Due to its high volatility, the isolated yield was determined by ^{1}H NMR using TCE (42 µL, 0.4 mmol, 1.0 equiv.) as an internal standard.

^{1}H NMR (400 MHz, $CDCl_3$) δ 2.34 (t, *J* = 7.4 Hz, 2H), 1.76-1.62 (m, 5H), 1.54 (app. q, *J* = 7.3 Hz, 2H), 1.45-1.32 (m, 1H), 1.31-1.07 (m, 3H), 0.97-0.82 (m, 2H).
^{13}C NMR (101 MHz, $CDCl_3$) δ 120.4, 36.9, 32.7, 32.6, 26.4, 26.1, 14.8.
Data in agreement with those reported in the literature.[4]

Diethyl isopentylphosphonate (**3q**)

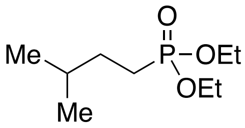

Prepared according to the general procedure using diethyl vinylphosphonate (62.0 µL, 0.4 mmol, 1.0 equiv.), anhydrous EtOH (2.0 mL), isopropyl iodide (120 µL, 1.2 mmol, 3.0 equiv.) and TTMSS (370 µL, 1.2 mmol, 3.0 equiv.). The crude product was purified by sFCC (20-30% acetone in PE) to afford the title compound as a yellow oil (69.4 mg, 0.333 mmol, 83%).

^{1}H NMR (400 MHz, $CDCl_3$) *δ* 4.16-4.00 (m, 4H), 1.76-1.43 (m, 5H), 1.31 (t, *J* = 7.1 Hz, 6H), 0.89 (d, *J* = 6.6 Hz, 6H).
^{13}C NMR (101 MHz, $CDCl_3$) *δ* 61.5 (d, *J* = 6.5 Hz), 31.1 (d, *J* = 5.2 Hz), 28.9 (d, *J* = 17.5 Hz), 23.7 (d, *J* = 140.8 Hz), 22.1, 16.6 (d, *J* = 6.0 Hz).
^{31}P NMR (162 MHz, $CDCl_3$) *δ* 33.4-32.9 (m, 1P).
Data in agreement with those reported in the literature.[5]

1-(Isopentylsulfonyl)-4-methylbenzene (**3r**)

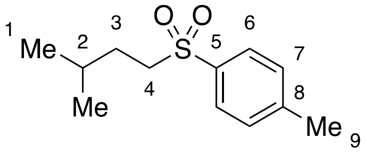

Prepared according to the general procedure using 1-methyl-4-(vinylsulfonyl)benzene (73.9 mg, 0.4 mmol, 1.0 equiv.), anhydrous EtOH (2.0 mL), isopropyl iodide (120 µL, 1.2 mmol, 3.0 equiv.) and TTMSS (370 µL, 1.2 mmol, 3.0 equiv.). The crude product was purified by sFCC (6-8% EtOAc in PE) to afford the title compound as a yellow oil (76.9 mg, 0.340 mmol, 85%).

R_f 0.21 (10% EtOAc in PE).
^{1}H NMR (400 MHz, $CDCl_3$) δ 7.77 (d, J = 8.3 Hz, 2H, H-6), 7.34 (d, J = 8.5 Hz, 2H, H-7), 3.09-3.01 (m, 2H, H-4), 2.44 (s, 3H, H-9), 1.67-1.52 (m, 3H, H-2, H-3), 0.85 (d, J = 6.2 Hz, 6H, H-1).
^{13}C NMR (101 MHz, $CDCl_3$) δ 144.6 (C-8), 136.4 (C-5), 130.0 (C-7), 128.1 (C-6), 54.9 (C-4) 31.2 (C-3), 27.3 (C-2), 22.1 (C-1), 21.7 (C-9).
HRMS calculated for $[C_{12}H_{18}NaO_2S]^+$ ($[M + Na]^+$) 249.0920, found 249.0920.
IR (film) ν_{max}/cm^{-1} 2958 (sp^2 C–H), 2872 (sp^3 C–H), 1598, 1467 (sp^3 C–H), 1316 (S=O), 1300, 1282, 1142 (S=O), 1087, 816, 769, 752, 662.

4-Methyl-*N*-phenylpent-2-enamide (**3s**)

Prepared according to the general procedure using *N*-phenylpropynamide (58.8 mg, 0.4 mmol, 1.0 equiv.), anhydrous EtOH (2.0 mL), isopropyl iodide (80.0 µL, 0.8 mmol, 2.0 equiv.) and TTMSS (250 µL, 0.8 mmol, 2.0 equiv). The crude product was purified by sFCC (10-13% EtOAc in PE) to afford the (*E*)-isomer as a white solid (20.1 mg, 0.107 mmol, 27%) and the (*Z*)-isomer as a white solid (12.1 mg, 6.39×10^{-2} mmol, 16%).

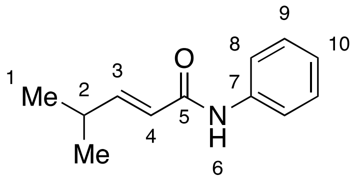

(E)-Isomer **R*f*** 0.37 (20% EtOAc in PE).

^{1}H NMR (400 MHz, $CDCl_3$) δ 7.57 (d, *J* = 7.0 Hz, 2H, H-8), 7.31 (app. t, *J* = 7.3 Hz, 2H, H-9), 7.14-7.06 (m, 2H, H-6, H-10), 6.96 (dd, *J* = 15.2, 6.6 Hz, 1H, H-3), 5.89 (dd, *J* = 15.4, 1.3 Hz, 1H, H-4), 2.55-2.41 (m, 1H, H-2), 1.08 (d, *J* = 6.6 Hz, 6H, H-1).

^{13}C NMR (101 MHz, $CDCl_3$) δ 164.7 (C-5), 153.0 (C-3), 138.2 (C-7), 129.3 (C-9), 124.4 (C-10), 121.4 (C-4), 120.1 (C-8), 31.0 (C-2), 21.6 (C-1).

HRMS calculated for $[C_{12}H_{16}NO]^+$ ($[M + H]^+$) 190.1232, found 190.1229.

IR (neat) ν_{max}/cm^{-1} 3200 (br., N–H), 3082 (sp^2 C–H), 2962, 2921, 2851 (sp^3 C–H), 1660 (C=O), 1631 (C=C), 1598, 1541, 1489, 1440 (sp^3 C–H), 1343 (C–N), 1301, 1245, 1105, 1001, 976, 813, 787, 759 (sp^2 C–H), 691 (C=C).

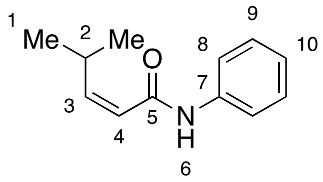

(Z)-Isomer **R*f*** 0.46 (20% EtOAc in PE).

^{1}H NMR (400 MHz, $CDCl_3$) δ 7.55 (d, *J* = 7.6 Hz, 2H, H-8), 7.32 (app. t, *J* = 7.6 Hz, 2H, H-9), 7.15-7.05 (m, 2H, H-6, H-10), 5.93 (dd, *J* = 11.2, 10.0 Hz, 1H, H-3), 5.71 (d, *J* = 11.1 Hz, 1H, H-4), 3.81-3.65 (m, 1H, H-2), 1.05 (d, *J* = 6.7 Hz, 6H, H-1).

^{13}C NMR (101 MHz, $CDCl_3$) δ 164.7 (C-5), 154.8 (C-3), 138.1 (C-7), 129.2 (C-9), 124.5 (C-10), 120.2 (C-4), 119.9 (C-8), 27.6 (C-2), 22.6 (C-1).

HRMS calculated for $[C_{12}H_{16}NO]^+$ ($[M + H]^+$) 190.1232, found 190.1228.

IR (neat) ν_{max}/cm^{-1} 3262 (br., N−H), 3137 (sp^2 C−H), 3085, 2962, 2921, 2850 (sp^3 C−H), 1658 (C=O), 1633 (C=C), 1595, 1541, 1488, 1440 (sp^3 C−H), 1311 (C−N), 1243, 1197, 1105, 1000, 919, 844, 742 (C=C), 692 (sp^2 C−H).

3-(Adamantan-1-yl)-*N*-phenylpropanamide (**3t**)

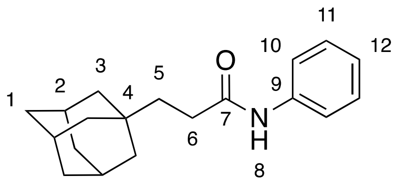

Prepared according to the general procedure using *N*-phenylacrylamide (60.0 mg, 0.4 mmol, 1.0 equiv.), anhydrous EtOH (2.0 mL), 1-iodoadamantane (210 mg, 0.8 mmol, 2.0 equiv.) and TTMSS (250 µL, 0.8 mmol, 2.0 equiv.). The crude product was purified by sFCC (10-30% EtOAc in PE) to afford the title compound as an off-white solid (87.1 mg, 0.307 mmol, 77%).

R_f 0.40 (20% EtOAc in PE).

^{1}H NMR (400 MHz, $CDCl_3$) δ 7.51 (d, J = 7.8 Hz, 2H, H-10), 7.31 (app. t, J = 7.8 Hz, 2H, H-11), 7.14 (br. s, 1H, H-8), 7.09 (t, J = 7.5 Hz, 1H, H-12), 2.36-2.26 (m, 2H, H-6), 2.01-1.93 (m, 3H, H-2), 1.76-1.59 (m, 14H, H-1, H-3, H-5).

^{13}C NMR (101 MHz, $CDCl_3$) δ 172.5 (C-7), 138.4 (C-9), 129.3 (C-11), 124.4 (C-12), 120.1 (C-10), 42.3 (C-3), 39.8 (C-5), 37.2 (C-1), 32.2 (C-4), 31.6 (C-6), 28.7 (C-2).

HRMS calculated for $[C_{19}H_{26}NO]^+$ ($[M + H]^+$) 284.200891, found 284.20162.

IR (neat) ν_{max}/cm^{-1} 3237 (br., N−H), 3038 (sp^2 C−H), 2897, 2842 (sp^3 C−H), 1655 (C=O), 1595 (N−H), 1551, 1497, 1445 (sp^3 C−H), 1327, 1255, 745 (sp^2 C−H), 692, 546, 505.

4,4-Dimethyl-*N*-phenylpentanamide (**3u**)

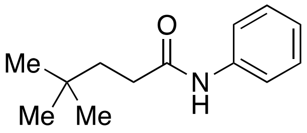

Prepared according to the general procedure using *N*-phenylacrylamide (60.0 mg, 0.4 mmol, 1.0 equiv.), anhydrous EtOH (2.0 mL), *tert*-butyl iodide (96.0 µL, 0.8 mmol, 2.0 equiv.) and TTMSS (250 µL, 0.8 mmol, 2.0 equiv.). The crude product was purified by sFCC (10-30% EtOAc in PE) to afford the title compound as a white solid (59.1 mg, 0.286 mmol, 72%).

^{1}H NMR (400 MHz, $CDCl_3$) δ 7.51 (d, J = 7.9 Hz, 2H), 7.32 (app. t, J = 7.8 Hz, 2H), 7.18-7.04 (m, 2H), 2.38-2.29 (m, 2H), 1.70-1.61 (m, 2H), 0.94 (s, 9H).
^{13}C NMR (101 MHz, $CDCl_3$) δ 172.3, 138.4, 129.3, 124.4, 120.1, 39.4, 33.6, 30.3, 29.3.
Data in agreement with those reported in the literature.[6]

Tert-butyl 3-(3-oxo-3-phenoxypropyl)azetidine-1-carboxylate (**3v**)

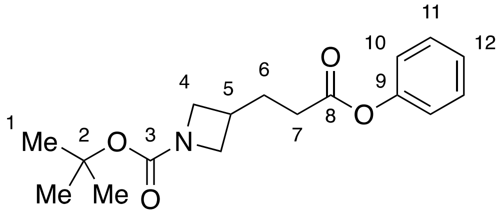

Prepared according to the general procedure using phenyl acrylate (56.0 µL, 0.4 mmol, 1.0 equiv.), anhydrous *t*-BuOH (2.0 mL), 1-Boc-3-iodoazetidine (227 mg, 0.8 mmol, 2.0 equiv.) and TTMSS (250 µL, 0.8 mmol, 2.0 equiv.). The crude product was purified by sFCC (30-50% Et_2O in PE) to afford the title compound as a pale yellow oil (96.3 mg, 0.315 mmol, 79%).

R*f* 0.17 (40% Et_2O in PE).
^{1}H NMR (400 MHz, $CDCl_3$) δ 7.38 (dd, *J* = 8.2, 7.6 Hz, 2H, H-11), 7.24 (tt, *J* = 7.5, 1.1 Hz, 1H, H-12), 7.07 (dd, *J* = 8.5, 1.0 Hz, 2H, H-10), 4.05 (app. t, *J* = 8.4 Hz, 2H, H-4), 3.60 (dd, *J* = 8.6, 5.5 Hz, 2H, H-4'), 2.67-2.56 (m, 1H, H-5), 2.53 (t, *J* = 7.5 Hz, 2H, H-7), 2.04 (app. q, *J* = 7.5 Hz, 2H, H-6), 1.43 (s, 9H, H-1).
^{13}C NMR (101 MHz, $CDCl_3$) δ 171.6 (C-8), 156.6 (C-3), 150.9 (C-9), 129.8 (C-11), 126.0 (C-12), 121.7 (C-10), 79.7 (C-2), 54.4 (C-4), 32.0 (C-7), 29.5 (C-6), 28.5 (C-1), 28.4 (C-5).
HRMS calculated for $[C_{17}H_{24}NO_4]^+$ ($[M + H]^+$) 306.1705, found 306.1708.
IR (film) ν_{max}/cm^{-1} 2970 (sp^3 C–H), 1759 (ester C=O), 1699 (carbamate C=O), 1404, 1366 (sp^3 C–H), 1196, 1163 (sp^3 C–O), 1134 (sp^3 C–N).

Phenyl 3-(oxetan-3-yl)propanoate (**3w**)

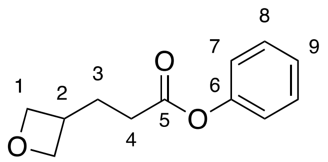

Prepared according to the general procedure using phenyl acrylate (56.0 µL, 0.4 mmol, 1.0 equiv.), anhydrous *t*-BuOH (2.0 mL), 3-iodooxetane (72.0 µL, 0.8 mmol, 2.0 equiv.) and TTMSS (250 µL, 0.8 mmol, 2.0 equiv.). The crude product was purified by sFCC (10-30% acetone in PE) to afford the title compound as a yellow oil (52.0 mg, 0.252 mmol, 63%).

R*f* 0.30 (20% acetone in PE).
^{1}H NMR (400 MHz, $CDCl_3$) *δ* 7.38 (app. t, *J* = 7.8 Hz, 2H, H-8), 7.23 (t, *J* = 7.6 Hz, 1H, H-9), 7.07 (d, *J* = 7.9 Hz, 2H, H-7), 4.83 (dd, *J* = 6.4, 6.2 Hz, 2H, H-1), 4.45 (app. t, *J* = 6.2 Hz, 2H, H-1'), 3.17-3.04 (m, 1H, H-2), 2.52 (t, *J* = 7.5 Hz, 2H, H-4), 2.15 (app. q, *J* = 7.5 Hz, 2H, H-3).
^{13}C NMR (101 MHz, $CDCl_3$) *δ* 171.8 (C-5), 150.8 (C-6), 129.8 (C-8), 126.2 (C-9), 121.6 (C-7), 77.2 (C-1), 34.8 (C-2), 32.1 (C-4), 28.9 (C-3).
HRMS calculated for $[C_{12}H_{15}O_3]^+$ ($[M + H]^+$) 207.1021, found 207.1017.
IR (film) ν_{max}/cm^{-1} 2965 (sp^2 C–H), 2868 (sp^3 C–H), 1752 (C=O), 1493 (sp^3 C–H), 1192 (sp^2 C–O), 1137 (sp^3 C–O), 974, 750 (sp^2 C–H), 691, 500.

Tert-butyl 4-(3-oxo-3-(phenylamino)propyl)piperidine-1-carboxylate (**3x**)

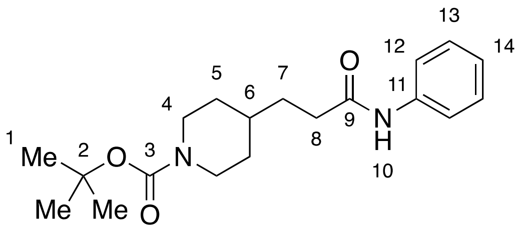

Prepared according to the general procedure using *N*-phenylacrylamide (60.0 mg, 0.4 mmol, 1.0 equiv.), anhydrous EtOH (2.0 mL), 1-Boc-4-iodopiperidine (249 mg, 0.8 mmol, 2.0 equiv.) and TTMSS (250 µL, 0.8 mmol, 2.0 equiv.). The crude product was purified by sFCC (10-40% EtOAc in PE) to afford the title compound as a colourless oil (56.8 mg, 0.171 mmol, 43%).

^{1}H NMR (400 MHz, $CDCl_3$) δ 7.50 (d, *J* = 7.9 Hz, 2H, H-12), 7.32 (app. t, *J* = 7.6 Hz, 2H, H-13), 7.16 (br. s, 1H, H-10), 7.11 (t, *J* = 7.4 Hz, 1H, H-14), 4.21-3.99 (m, 2H, H-4), 2.68 (app. t, *J* = 11.9 Hz, 2H, H-4’), 2.39 (t, *J* = 7.7 Hz, 2H, H-8), 1.75-1.63 (m, 4H, H-5, H-7), 1.55-1.48 (m, 1H, H-6), 1.46 (s, 9H, H-1), 1.13 (app. dq, *J* = 12.3, 4.0 Hz, 2H, H-5’).
^{13}C NMR (101 MHz, $CDCl_3$) δ 171.5 (C-9), 155.1 (C-3), 138.1 (C-11), 129.0 (C-13), 124.4 (C-14), 120.0 (C-12), 79.6 (C-2), 44.1 (C-4), 35.5 (C-6), 34.7 (C-8), 32.1 (C-7), 32.0 (C-5), 28.7 (C-1).
HRMS calculated for $[C_{19}H_{29}N_2O_3]^+$ ($[M + H]^+$) 333.2178, found 333.2186.
IR (film) ν_{max}/cm^{-1} 3275 (br., N−H), 2960 (sp^2 C−H), 2922, 2864 (sp^3 C−H), 1698 (carbamate C=O), 1660 (anilide C=O), 1599, 1543, 1497, 1419, 1364 (sp^3 C−H), 1299 (sp^2 C−N), 1274, 1241, 1157 (sp^3 C−O), 1122 (sp^3 C−N), 753 (sp^2 C−H).

3-(1,1-Dioxidotetrahydrothiophen-3-yl)-*N*-phenylpropanamide (**3y**)

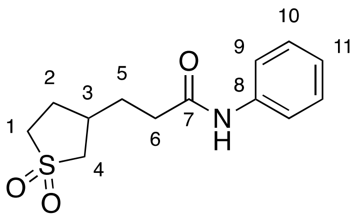

Prepared according to the general procedure using *N*-phenylacrylamide (60.0 mg, 0.4 mmol, 1.0 equiv.), anhydrous EtOH (2.0 mL), 3-iodotetrahydrothiophene 1,1-dioxide (296 mg, 1.2 mmol, 3.0 equiv.) and TTMSS (375 µL, 1.2 mmol, 3.0 equiv.). The crude product was purified by sFCC (70-72% EtOAc in PE) to afford the title compound as a white solid (76.1 mg, 0.285 mmol, 71%).

R_f 0.30 (80% EtOAc in PE).

^{1}H NMR (400 MHz, $CDCl_3$) δ 7.49 (d, *J* = 8.1 Hz, 2H, H-9), 7.33 (app. t, *J* = 7.8 Hz, 2H, H-10), 7.12 (t, *J* = 7.5 Hz, 1H, H-11), 3.27 (dd, *J* = 12.9, 7.1 Hz, 1H, H-4), 3.26-3.19 (m, 1H, H-1), 3.12-3.02 (m, 1H, H-1'), 2.71 (dd, *J* = 12.8, 10.4 Hz, 1H, H-4'), 2.61-2.47 (m, 1H, H-3), 2.45-2.31 (m, 3H, H-2, H-6), 2.06-1.81 (m, 3H, H-2', H-5).

^{13}C NMR (101 MHz, $CDCl_3$) δ 170.1 (C-7), 137.8 (C-8), 129.2 (C-10), 124.6 (C-11), 120.0 (C-9), 56.7 (C-4), 52.4 (C-1), 36.4 (C-3), 34.9 (C-6), 29.9 (C-5), 29.2 (C-2).

HRMS calculated for $[C_{13}H_{17}NO_3S]^+$ (M^+) 267.09291, found 267.0935.

IR (neat) ν_{max}/cm^{-1} 3376 (br., N–H), 2950 (sp^3 C–H), 1679 (C=O), 1596 (N–H), 1536, 1437, 1289 (S=O), 1232, 1117 (C–N), 754 (sp^2 C–H), 692, 567, 509, 464, 447.

4-(Oxan-4-yl)-*N*-phenylpentanamide (**3z**)

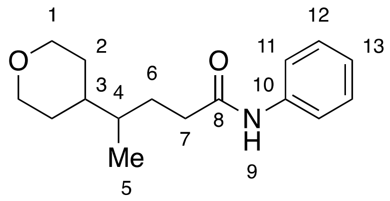

Prepared according to the general procedure using *N*-phenylacrylamide (29.5 mg, 0.2 mmol, 1.0 equiv.), anhydrous EtOH (1.0 mL), 4-(1-iodoethyl)oxane (145 mg, 0.6 mmol, 3.0 equiv.) and TTMSS (125 µL, 0.4 mmol, 2.0 equiv.). The crude product was purified by sFCC (10-12% acetone in PE) to afford the title compound as a pale yellow oil (26.3 mg, 0.101 mmol, 50%).

R_f 0.34 (30% acetone in PE).

^{1}H NMR (400 MHz, $CDCl_3$) δ 7.51 (d, *J* = 8.0 Hz, 2H, H-11), 7.32 (app. t, *J* = 7.8 Hz, 2H, H-12), 7.19 (br., s, 1H, H-9), 7.10 (t, *J* = 7.5 Hz, 1H, H-13), 3.99 (d, *J* = 11.0 Hz, 2H, H-1), 3.35 (app. t, *J* = 11.3 Hz, 2H, H-1'), 2.49-2.38 (m, 1H, H-7), 2.35-2.23 (m, 1H, H-7'), 1.95-1.84 (m, 1H, H-6), 1.58-1.31 (m, 7H, H-2, H-3, H-4, H-6'), 0.90 (d, *J* = 6.4 Hz, 3H, H-5).

^{13}C NMR (101 MHz, $CDCl_3$) δ 171.5 (C-8), 138.1 (C-10), 129.0 (C-12), 124.4 (C-13), 120.0 (C-11), 68.5 (C-1), 40.3 (C-3), 37.5 (C-4), 35.7 (C-7), 30.7 (C-2), 29.5 (C-6), 29.3 (C-2'), 15.8 (C-5).

HRMS calculated for $[C_{16}H_{24}NO_2]^+$ ($[M + H]^+$) 262.1807, found 262.1800.

IR (film) ν_{max}/cm^{-1} 3276 (br., N–H), 2933 (sp^2 C–H), 2846 (sp^3 C–H), 1663 (C=O), 1599, 1544, 1499, 1442 (sp^3 C–H), 1308, 1093 (C–O), 755 (sp^2 C–H), 693 (sp^2 C–H).

Tert-butyl 4-(4-(3-oxo-3-(phenylamino)propyl)piperidin-1-yl)benzoate (**3aa**)

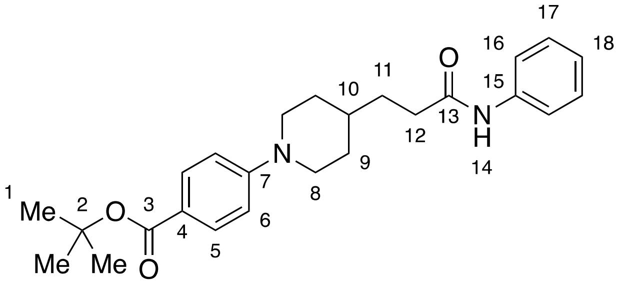

Prepared according to the general procedure using *N*-phenylacrylamide (29.5 mg, 0.2 mmol, 1.0 equiv.), anhydrous EtOH (1.0 mL), *tert*-butyl 4-(4-iodopiperidin-1-yl)benzoate (233 mg, 0.6 mmol, 3.0 equiv.) and TTMSS (125 µL, 0.4 mmol, 2.0 equiv.). The crude product was purified by sFCC (30-50% EtOAc in PE containing 0.1% Et_3N) to afford the title compound as a yellow solid (65.0 mg, 0.159 mmol, 80%).

R*f* 0.31 (40% EtOAc in PE containing 0.1% Et_3N).

^{1}H NMR (400 MHz, $CDCl_3$) *δ* 7.84 (d, *J* = 8.9 Hz, 2H, H-5), 7.53 (d, *J* = 7.8 Hz, 2H, H-16), 7.31 (app. t, *J* = 7.8 Hz, 2H, H-17), 7.19 (br. s, 1H, H-14), 7.10 (t, *J* = 7.5 Hz, 1H, H-18), 6.83 (d, *J* = 9.0 Hz, 2H, H-6), 3.89-3.74 (m, 2H, H-8), 2.78 (app. dt, *J* = 12.5, 1.9 Hz, 2H, H-8'), 2.40 (t, *J* = 7.7 Hz, 2H, H-12), 1.78 (d, *J* = 12.3 Hz, 2H, H-9), 1.71 (app. q, *J* = 7.4 Hz, 2H, H-11), 1.57 (s, 9H, H-1), 1.54-1.45 (m, 1H, H-10), 1.31 (app. dq, *J* = 12.5, 4.2 Hz, 2H, H-9').

^{13}C NMR (101 MHz, $CDCl_3$) *δ* 171.4 (C-13), 166.4 (C-3), 154.2 (C-7), 138.2 (C-15), 131.1 (C-5), 129.2 (C-17), 124.4 (C-18), 121.3 (C-4), 119.9 (C-16), 114.0 (C-6), 80.3 (C-2), 48.4 (C-8), 35.5 (C-10), 34.9 (C-12), 32.0 (C-11), 31.6 (C-9), 28.5 (C-1).

HRMS calculated for $[C_{25}H_{33}N_2O_3]^+$ ($[M + H]^+$) 409.2491, found 409.2500.

IR (neat) ν_{max}/cm^{-1} 3298 (br., N−H), 2930 (sp^2 C−H), 2845 (sp^3 C−H), 1691 (ester C=O), 1661 (amide C=O), 1544, 1444 (sp^3 C−H), 1389, 1364 (sp^3 C−H), 1312, 1295 (sp^2 C−N), 1273, 1255, 1236 (sp^3 C−N), 1161 (sp^3 C−O), 1117, 831, 773, 756 (sp^2 C−H), 722, 693 (sp^2 C−H).

N-Phenyl-3-(1-(3-(trifluoromethyl)-[1,2,4]triazolo[4,3-*b*]pyridazin-6-yl)pyrrolidin-3-yl)propenamide (**3ab**)

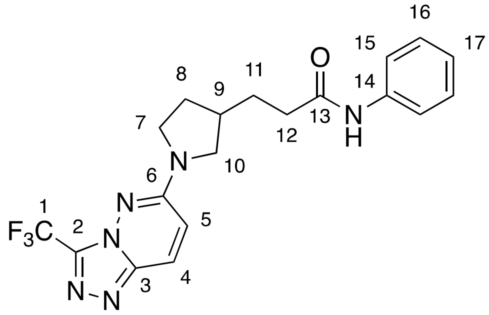

Prepared according to the general procedure using *N*-phenylacrylamide (29.5 mg, 0.2 mmol, 1.0 equiv.), anhydrous EtOH (1.0 mL), 6-(3-iodopyrrolidin-1-yl)-3-(trifluoromethyl)-[1,2,4]triazolo[4,3-*b*]pyridazine (200 mg, 1.2 mmol, 3.0 equiv.) and TTMSS (190 µL, 1.2 mmol, 3.0 equiv.). The crude product was purified by sFCC (70-100% EtOAc in PE) to afford the title compound as a pale yellow oil (35.6 mg, 8.8 x 10^{-2} mmol, 44%).

R*f* 0.07 (80% EtOAc in PE).

^{1}H NMR (400 MHz, MeOH-d_4) δ 7.96 (d, *J* = 10.1 Hz, 1H, H-4), 7.54 (dd, *J* = 8.5, 1.0 Hz, 2H, H-15), 7.28 (dd, *J* = 8.2, 7.7 Hz, 2H, H-16), 7.17 (d, *J* = 10.4 Hz, 1H, H-5), 7.07 (tt, *J* = 7.4, 1.0 Hz, 1H, H-17), 3.80 (dd, *J* = 10.7, 7.4 Hz, 1H, H-10), 3.72 (ddd, *J* = 11.0, 8.3, 3.1 Hz, 1H, H-7), 3.57-3.47 (m, 1H, H-7'), 3.23-3.12 (app. t, *J* = 9.4 Hz, 1H, H-10'), 2.50 (t, *J* = 7.6 Hz, 2H, H-12), 2.45-2.37 (m, 1H, H-9), 2.32-2.22 (m, 1H, H-8), 1.90 (app. q, *J* = 7.8 Hz, 2H, H-11), 1.83-1.71 (m, 1H, H-8').

^{13}C NMR (101 MHz, MeOH-d_4) δ 173.9 (C-13), 155.0 (C-6), 146.0 (C-3), 139.8 (C-14), 139.4 (q, *J* = 41.0 Hz, C-2), 129.8 (C-16), 125.2 (C-17), 124.1 (C-4), 121.2 (C-15), 120.0 (q, *J* = 272.2 Hz, C-1), 118.6 (C-5), 53.6 (C-10), 48.1 (C-7), 39.7 (C-9), 36.4 (C-12), 32.2 (C-8), 30.1 (C-11).

^{19}F NMR (376 MHz, MeOH-d_4) δ –66.2 (s, 3F, F-1).

HRMS calculated for $[C_{19}H_{20}F_3N_6O]^+$ ($[M + H]^+$) 405.1651, found 405.1636.

N,5-Diphenylpentanamide (**3ac**)

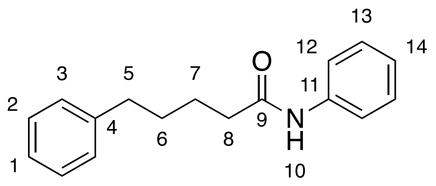

Prepared according to the general procedure using *N*-phenylacrylamide (60.0 mg, 0.4 mmol, 1.0 equiv.), anhydrous EtOH (2.0 mL), (2-iodoethyl)benzene (120 µL, 0.8 mmol, 2.0 equiv.) and TTMSS (250 µL, 0.8 mmol, 2.0 equiv.). The crude product was purified by sFCC (10-20% acetone in PE) to afford the title compound as an off-white solid (60.6 mg, 0.239 mmol, 60%).

R*f* 0.26 (20% acetone in PE).

^{1}H NMR (400 MHz, $CDCl_3$) *δ* 7.49 (d, *J* = 7.8 Hz, 2H, H-12), 7.31 (app. t, *J* = 7.8 Hz, 2H, H-13), 7.28 (app. t, *J* = 7.4 Hz, 2H, H-2), 7.22-7.15 (m, 3H, H-1, H-3), 7.14 (m, 2H, H-10, H-14), 2.67 (t, *J* = 7.2 Hz, 2H, H-5), 2.37 (t, *J* = 7.2 Hz, 2H, H-8), 1.84-1.66 (m, 4H, H-6, H-7).

^{13}C NMR (101 MHz, $CDCl_3$) *δ* 171.3 (C-9), 142.2 (C-4), 138.1 (C-11), 129.1 (C-13), 128.5 (C-3), 128.4 (C-2) 125.9 (C-1), 124.3 (C-14), 120.1 (C-12), 37.7 (C-8), 35.8 (C-5), 31.4 (C-6), 25.6 (C-7).

HRMS calculated for $[C_{17}H_{19}NO]^+$ (M^+) 253.14666, found 254.1467.

IR (neat) ν_{max}/cm^{-1} 3313 (br., N–H), 3028 (sp^2 C–H), 2926, 2854 (sp^3 C–H), 1649 (C=O), 1598 (N–H), 1526, 1498, 1442 (sp^3 C–H), 746 (sp^2 C–H), 699, 586, 551, 506.

N-Phenylpentanamide (**3ad**)

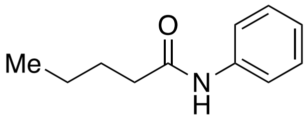

Prepared according to the general procedure using *N*-phenylacrylamide (60.0 mg, 0.4 mmol, 1.0 equiv.), anhydrous EtOH (2.0 mL), ethyl iodide (98.0 µL, 0.8 mmol, 2.0 equiv.) and TTMSS (250 µL, 0.8 mmol, 2.0 equiv.). The crude product was purified by sFCC (20-40% Et_2O in PE) to afford the title compound as an off-white solid (49.6 mg, 0.280 mmol, 70%).

^{1}H NMR (400 MHz, $CDCl_3$) δ 7.51 (d, *J* = 7.5 Hz, 2H), 7.32 (app. t, *J* = 7.7 Hz, 2H), 7.22-7.03 (m, 2H), 2.36 (t, *J* = 7.4 Hz, 2H), 1.72 (app. quint., *J* = 7.6 Hz, 2H), 1.42 (app. sext., *J* = 7.4 Hz, 2H), 0.95 (t, *J* = 7.4 Hz, 3H).
^{13}C NMR (101 MHz, $CDCl_3$) δ 171.8, 138.4, 129.3, 124.4, 120.1, 37.8, 28.0, 22.6, 14.0.
Data in agreement with those reported in the literature.[7]

7-Chloro-*N*-phenylheptanamide (**3ae**)

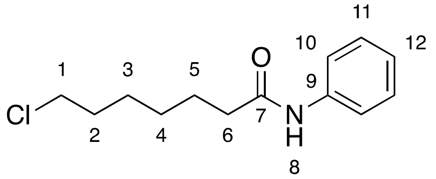

Prepared according to the general procedure using *N*-phenylacrylamide (60.0 mg, 0.4 mmol, 1.0 equiv.), anhydrous EtOH (2.0 mL), 1-chloro-4-iodobutane (98.0 µL, 0.8 mmol, 2.0 equiv.) and TTMSS (250 µL, 0.8 mmol, 2.0 equiv.). The crude product was purified by sFCC (10-30% EtOAc in PE) to afford the title compound as a white solid (54.4 mg, 0.227 mmol, 57%).

R_f 0.24 (20% EtOAc in PE).

^{1}H NMR (400 MHz, $CDCl_3$) δ 7.51 (d, *J* = 7.9 Hz, 2H, H-10), 7.32 (app. t, *J* = 7.8 Hz, 2H, H-11), 7.15-7.05 (m, 2H, H-8, H-12), 3.54 (t, *J* = 6.6 Hz, 2H, H-1), 2.36 (t, *J* = 7.5 Hz, 2H, H-6), 1.84-1.70 (m, 4H, H-2, H-5), 1.54-1.36 (m, 4H, H-3, H-4).

^{13}C NMR (101 MHz, $CDCl_3$) δ 171.4 (C-7), 138.1 (C-9), 129.2 (C-11), 124.4 (C-12), 120.0 (C-10), 45.2 (C-1), 37.7 (C-6), 32.5 (C-2), 28.6 (C-4), 26.7 (C-3), 25.5 (C-5).

HRMS calculated for $[C_{13}H_{18}{}^{35}ClNO]^+$ (M^+) 239.10769, found 239.1075.

IR (neat) ν_{max}/cm^{-1} 3260 (br., N–H), 2944 (sp^2 C–H), 2851 (sp^3 C–H), 1665 (C=O), 1599 (N–H), 1548, 1498, 1442 (sp^3 C–H), 1314 (C–N), 1263, 1183, 758, 692 (C–Cl), 506.

Radical Clock Experiment

Performed according to the general procedure using phenyl acrylate (56.0 µL, 0.4 mmol, 1.0 equiv.), anhydrous *t*-BuOH (2.0 mL), cyclopropylmethyl iodide (146 mg, 0.8 mmol, 2.0 equiv.) and TTMSS (250 µL, 0.8 mmol, 2.0 equiv.). The crude product was purified by sFCC (10-40% CH_2Cl_2 in PE) to afford cyclopentane **7** as a pale yellow oil (23.5 mg, 0.115 mmol, 29%, 1.0:1.7 *dr*).

Phenyl 2-methylcyclopentane-1-carboxylate (7):

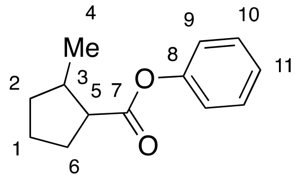

Major Isomer $\mathbf{R_f}$ 0.21 (20% CH_2Cl_2 in PE).

^{1}H NMR (400 MHz, $CDCl_3$) δ 7.37 (dd, *J* = 8.3, 7.5 Hz, 2H, H-10), 7.21 (tt, *J* =7.4, 1.2 Hz, 1H, H-11), 7.08 (dd, *J* = 8.6, 1.1 Hz, 2H, H-9), 3.04 (app. q, *J* = 7.6 Hz, 1H, H-5), 2.47 (app. sept., *J* = 7.3 Hz, 1H, H-3), 2.15-2.04 (m, 1H, H-6), 1.97-1.81 (m, 3H, H-1, H-2, H-6'), 1.69-1.57 (m, 1H, H-1'), 1.53-1.42 (m, 1H, H-2'), 1.10 (d, *J* = 7.0 Hz, 3H, H-4).

^{13}C NMR (101 MHz, $CDCl_3$) δ 173.9 (C-7), 151.1 (C-8), 129.6 (C-10), 125.7 (C-11), 121.9 (C-9), 48.4 (C-5), 38.0 (C-3), 34.2 (C-2), 27.8 (C-6), 24.0 (C-1), 16.5 (C-4).

HRMS calculated for $[C_{13}H_{17}O_2]^+$ ($[M + H]^+$) 205.1229, found 205.1236.

IR (film) ν_{max}/cm^{-1} 2971 (sp^2 C−H), 2901 (sp^3 C−H), 1747 (C=O), 1453 (sp^3 C−H), 1394, 1250 (C−O), 1195, 906, 729 (sp^2 C−H).

Minor Isomer $\mathbf{R_f}$ 0.21 (20% CH_2Cl_2 in PE).

^{1}H NMR (400 MHz, $CDCl_3$) δ 7.37 (dd, *J* = 8.3, 7.5 Hz, 2H, H-10), 7.22 (tt, *J* = 7.4, 1.3 Hz, 1H, H-11), 7.08 (dd, *J* = 8.6, 1.1 Hz, 2H, H-9), 2.50 (app. q, *J* = 8.6 Hz, 1H, H-5), 2.36-2.24 (m, 1H, H-3), 2.14-1.92 (m, 3H, H-2, H-6), 1.79-1.71 (m, 2H, H-1), 1.34-1.23 (m, 1H, H-2'), 1.18 (d, *J* = 6.6 Hz, 3H, H-4).

^{13}C NMR (101 MHz, $CDCl_3$) δ 175.1 (C-7), 151.1 (C-8), 129.6 (C-10), 125.7 (C-11), 121.7 (C-9), 52.0 (C-5), 39.8 (C-3), 35.1 (C-2), 30.3 (C-6), 24.7 (C-1), 19.9 (C-4).

HRMS calculated for $[C_{13}H_{17}O_2]^+$ ($[M + H]^+$) 205.1229, found 205.1233.

IR (film) ν_{max}/cm^{-1} 2969 (sp^2 C−H), 2901 (sp^3 C−H), 1749 (C=O), 1492, 1453 (sp^3 C−H), 1394, 1194, 1066, 907, 757 (sp^2 C−H).

Halogen bond exploration

In order to probe our halogen bonding hypothesis and observe an interaction between the hydridic component of the silane and the halogen of the alkyl halide reactant, a variety of titration experiments were performed and monitored by either ^{1}H NMR, IR or UV-Vis spectroscopy.[8]

^{1}H NMR spectroscopy

It has been previously identified that the incorporation of fluorine atoms into an alkyl halide strengthens its halogen bonding with a variety of acceptors.[8,9] Reports highlighted perfluorooctyl bromide as a suitable halogen bonding donor in order to strengthen any interactions with the silane hydride, and so aid visualisation.[10] Although our results suggested that solvent was not involved in this supposed interaction (Table 1), previous unreported results in our lab had concluded the instability of TTMSS in chloroform, and so this was not suitable. We subsequently selected benzene due to its proposed minimal involvement in any halogen bonding interactions.[11]

To an NMR tube was added TTMSS (37.0 µL, 0.1 mmol, 1.0 equiv.) and benzene-d_6 (0.6 mL). Perfluorooctyl bromide was added in increments of 0.5 equiv. from 0.0-2.0 equiv., after which a further 1.0 equiv. was added to provide a total of 3.0 equiv., and then an additional 2.0 equiv. was added to achieve 5.0 equiv. total, and finally an additional 5.0 equiv. was added to provide 10.0 equiv. total. After each addition, the NMR tube was vigorously shaken and a ^{1}H NMR spectrum recorded. The overlay is shown in Figure S1a.

A downfield shift in the silane hydride signal was observed between 0.0-1.5 equiv. perfluorooctyl bromide (Figure S1b); this observation was mirrored in the other silane methyl signal present in the NMR spectra, though not as significant (and hence not shown herein). The addition of further perfluorooctyl bromide to this solution resulted in a biphasic mixture, alongside no further change in the chemical shifts of the signals present.

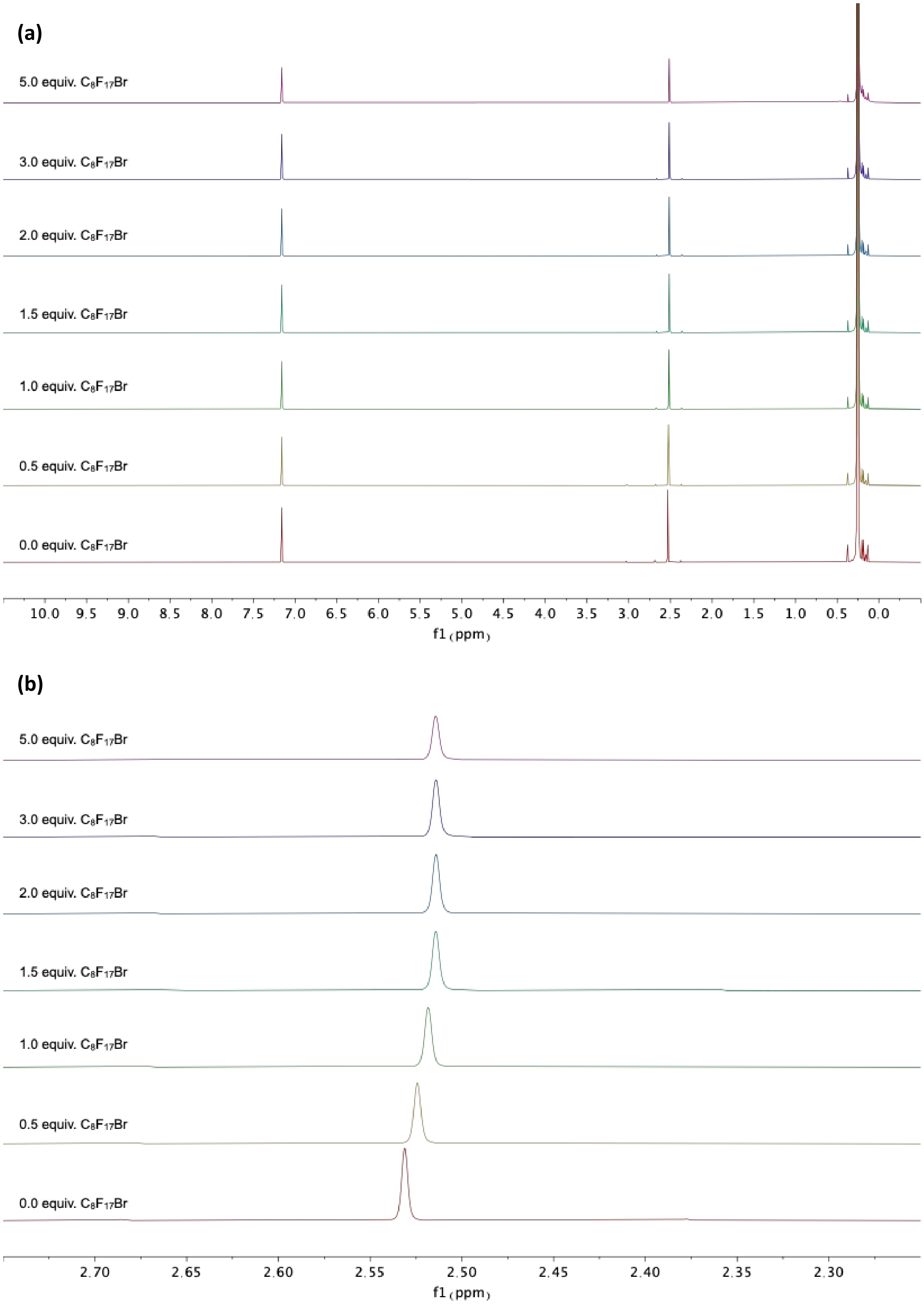

Figure S1. (a) Overlay of ^{1}H NMR titration between perfluorooctyl bromide and TTMSS in C_6D_6. **(b)** An expanded region of the overlay between δ_H = 2.25-2.75, highlighting the silane hydride signal.

With these results in hand, we sought to investigate whether this observation could be replicated with an iodide halogen bonding donor as well, also similarly activated through the incorporation of multiple fluorine substituents to maximise interactions with TTMSS. Based upon previous reports, iodopentafluorobenzene was chosen as a suitable partner.[12]

To an NMR tube was added TTMSS (37.0 µL, 0.1 mmol, 1.0 equiv.) and benzene-d_6 (0.6 mL). Iodopentafluorobenzene was added in increments of 0.5 equiv. from 0.0-1.5 equiv. After each addition, the NMR tube was vigorously shaken and a ^{1}H NMR spectrum recorded. The overlay is shown in Figure S2a.

As exemplified in Figure S2b, a downfield shift of the silane hydride signal was observed as the concentration of iodopentafluorobenzene increased, similar to the results obtained from the previous titration experiment. Conversely, however, an upfield shift of the TTMSS methyl protons was also identified, although this was not as prominent in comparison to the Si–H peak (and so not focused upon herein).

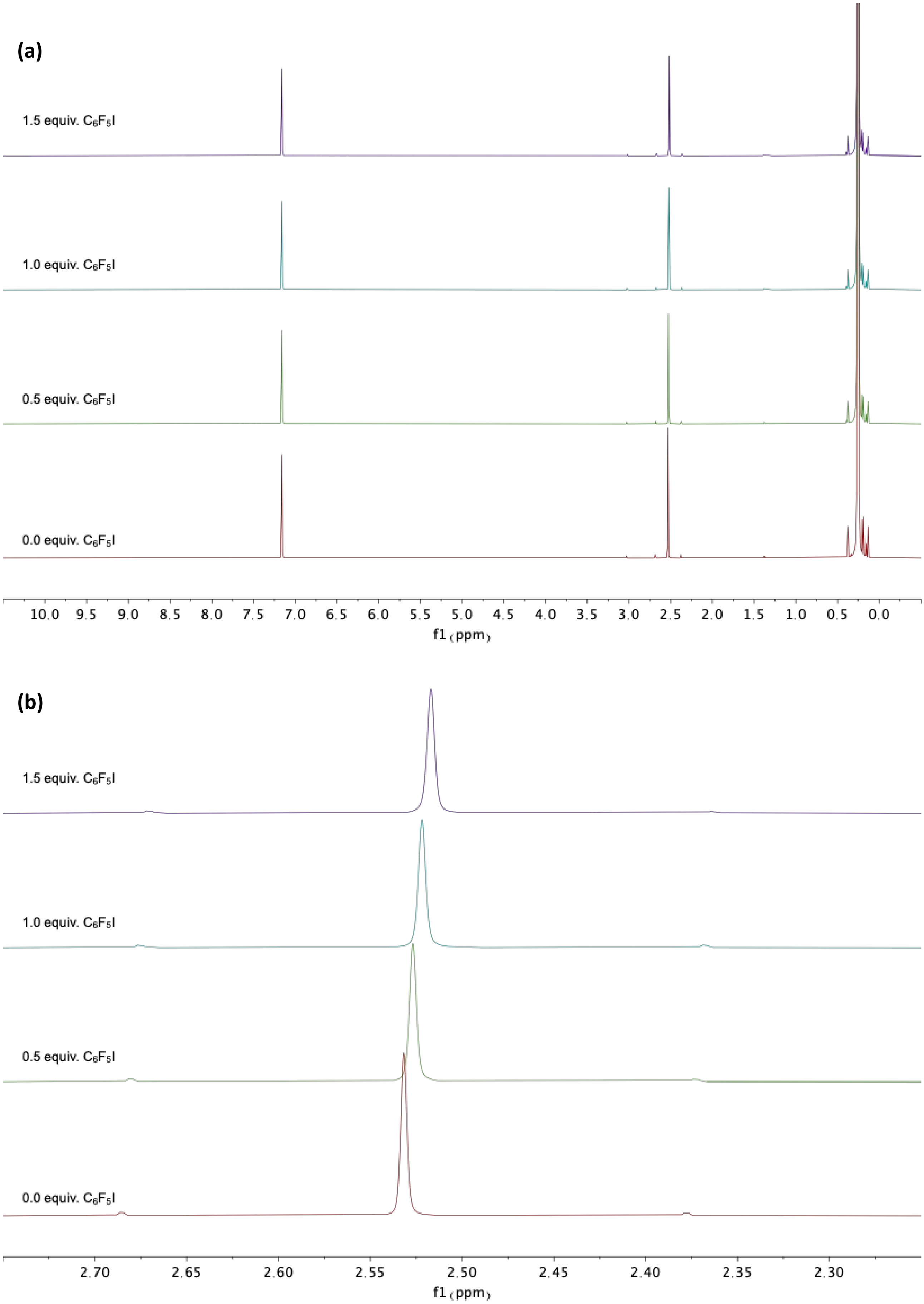

Figure S2. (a) Overlay of ^{1}H NMR spectra from the titration of iodopentafluorobenzene into TTMSS in C_6D_6. **(b)** Expanded region from the overlay focusing on the silane hydride signal.

Considering downfield shifts in the silane hydride NMR signal had been observed during the independent titration of two activated halide compounds, we sought to establish whether a similar phenomenon would occur in the presence of an unactivated alkyl halide donor. Subsequently, isopropyl iodide from our optimisation studies into a model Giese reaction (Table 2) was selected. A titration experiment with this potential halogen bonding partner would also provide the opportunity to monitor if any concurrent changes in the environment of its protons was also in effect, which (in theory) should oppose the direction of any shifts experienced by the TTMSS protons.

To an NMR tube was added TTMSS (37.0 μL, 0.1 mmol, 1.0 equiv.) and benzene-d_6 (0.6 mL). isopropyl iodide was added in increments of 0.5 equiv. from 0.0-2.0 equiv., after which a further 1.0 equiv. was added to provide a total of 3.0 equiv., and then an additional 2.0 equiv. was added to achieve 5.0 equiv. total, and finally additional 5.0 equiv. increments were added up to a maximum of 25.0 equiv. total. After each addition, the NMR tube was vigorously shaken and a ^{1}H NMR spectrum recorded. The overlay is shown in Figure S3a.

As per the previous titration experiments conducted, a downfield shift of the TTMSS hydride signal was observed (Figure S3b). A similar downfield shift of the silane methyl protons was also observed, but not as significant as the movement identified for the Si–H environment. In line with our hypothesis described above, the isopropyl iodide methine peak performed a concurrent upfield shift; its methyl groups also experienced an upfield shift, although to a reduced extent.

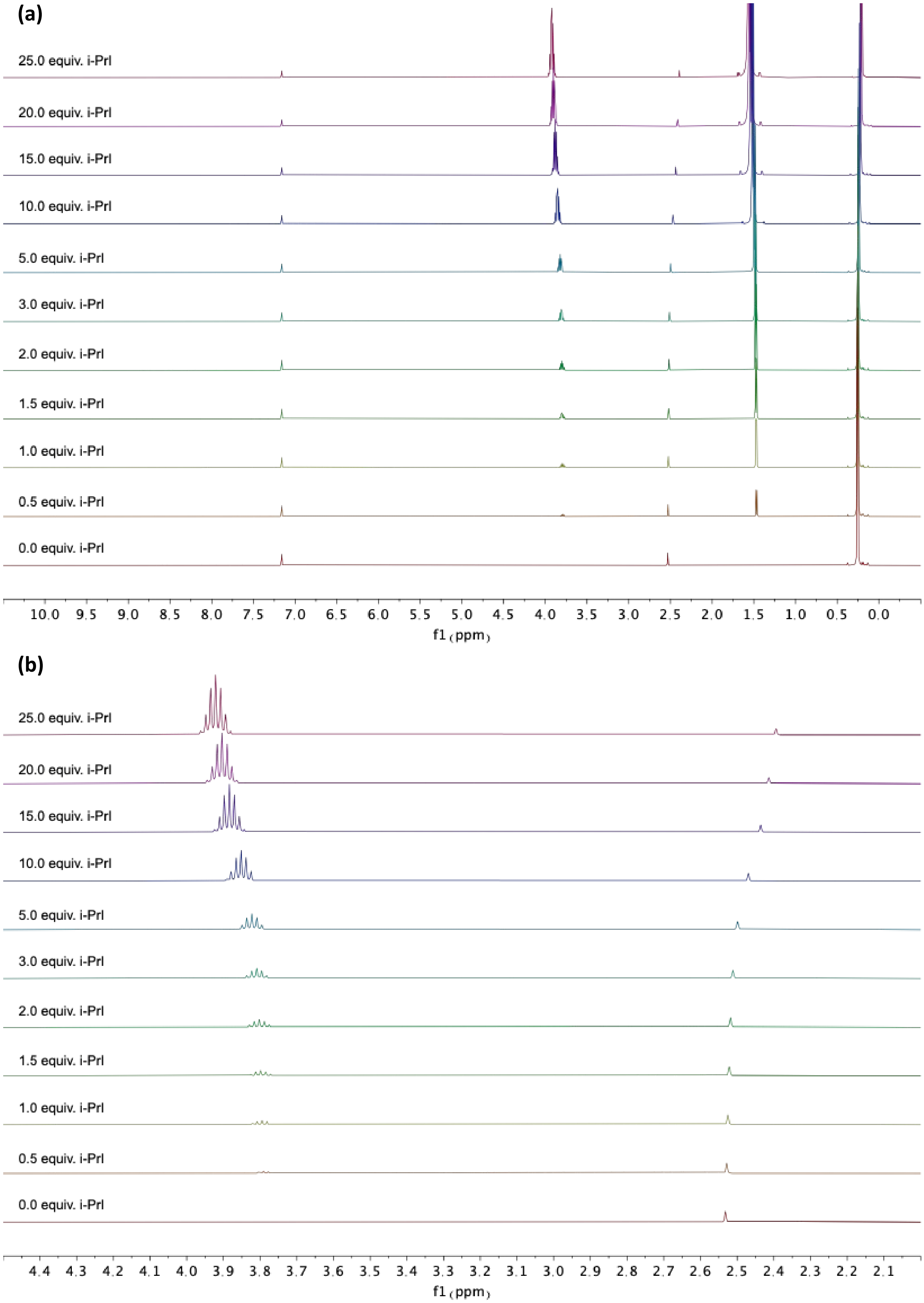

Figure S3. (a) Overlay of the ^{1}H NMR spectra for the titration of isopropyl iodide into TTMSS. **(b)** Expansion of the overlay highlighting the isopropyl iodide methine and silane hydride signals.

It was unexpected that a continual shift in both TTMSS and isopropyl iodide proton environments was observed up to the addition of 25.0 equiv., in particular considering the amount of silane available in solution was finite. Subsequently, a control experiment was run whereby the chemical shifts of the proton environments of isopropyl iodide were monitored as its concentration was incrementally increased in line with the previous titration study.

To an NMR tube was added isopropyl iodide (6.0 µL, 6.0 x 10^{-2} mmol) and benzene-d_6 (0.6 mL). the NMR tube was vigorously shaken and a ^1H spectrum recorded. Further isopropyl iodide (6.00 µL, 6.0 x 10^{-2} mmol) was added, the contents vigorously shaken and another ^1H NMR spectrum recorded. This addition sequence was repeated a further two times before increasing the charge of isopropyl iodide (12.0 µL, 0.1 mmol). The solution was vigorously shaken and a spectrum measured, after which a final dose of isopropyl iodide (24.0 µL, 0.2 mmol) was input, the NMR tube vigorously shaken and a spectrum recorded. The overlay is shown in Figure S4a.

An upfield shift of the isopropyl iodide methine signal was observed (Figure S4b), as well as for the methyl protons (although not as significant). Both of these movements were similar in magnitude to those identified for the same peaks during the previous titration experiment. Consequently, whilst an interaction between TTMSS and a halogen bonding donor may be in effect, the shifts of ^1H NMR signals observed through titration experiments cannot be attributed to this, but rather to possible modifications of the media and solvent environment instead.

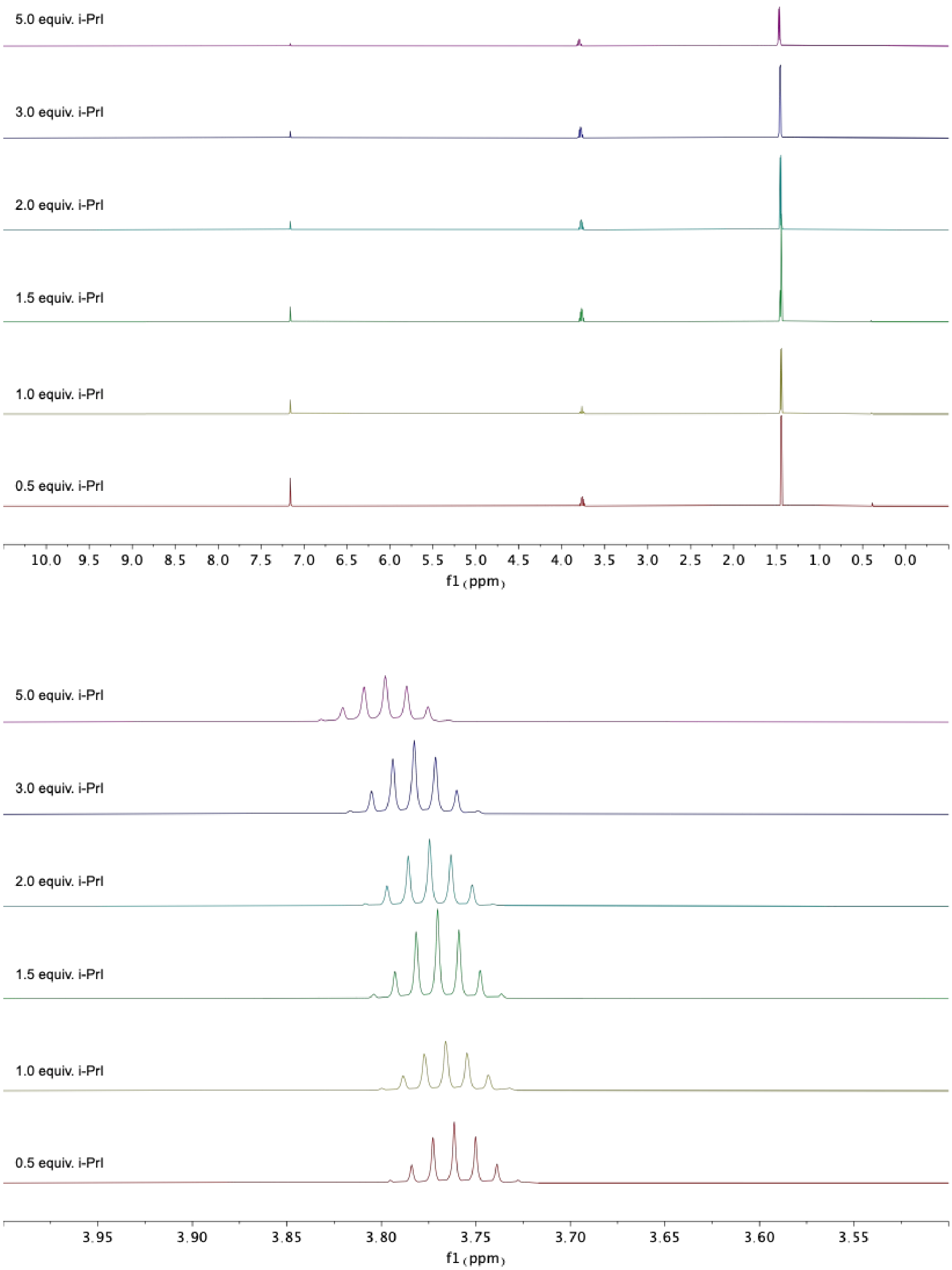

Figure S4. (a) Overlay of increasing concentration of isopropyl iodide in C_6D_6. **(b)** Expanded region of the overlay between δ_H = 3.50-4.00 focusing upon the isopropyl iodide methine signal.

UV-Visible titration studies

Our control experiments have highlighted the necessity for visible light in order to achieve initiation and radical formation (entries 9 and 11, Table 1). However, neither TTMSS[13] nor halocarbons[14] absorb in the visible region of the spectrum. Instead, this could be achieved by their proposed interaction, thereby resulting in homolytic cleavage of the Si–H and/or C–X bonds. Consequently, this absorption could be monitored by UV-Vis spectroscopy. In line with the previous NMR titration studies and to maximise our halogen bonding hypothesis, iodopentafluorobenzene was chosen as the initial donor, and benzene as solvent.

To a 1.0 mL UV quartz cuvette with a lid was added benzene (1.0 mL), and a reference spectrum taken. TTMSS (60.0 µL, 0.2 mmol, 1.0 equiv.) was added, the cuvette vigorously shaken, and a measurement recorded. Iodopentafluorobenzene (13.0 µL, 0.1 mmol, 0.5 equiv.) was added, the contents vigorously shaken, and a spectrum recorded. This addition sequence was repeated for a further five portions (resulting in 0.6 mmol (3.0 equiv.) of iodopentafluorobenzene total), before a final amount of iodopentafluorobenzene (26.0 µL, 0.2 mmol, 1.0 equiv.) was added to afford 0.8 mmol (4.0 equiv.) in total. The mixture was thoroughly mixed before a final spectrum was recorded. The overlay is shown in Figure S5 (and also includes a UV-Vis spectrum of iodopentafluorobenzene reference in benzene).

As the concentration of iodopentafluorobenzene increased, a red-shift in the absorbance of the mixture towards the visible region of the spectrum was observed. This could be the result of an interaction between the halide and TTMSS. However, it should be noted that the reactions discussed in our report were successful with concentrations of the halide component as minimal as 1.0 equiv. with respect to the silane, yet no distinct absorbance above 400 nm (the blue region of the spectrum generally emitted by the 40 W Kessil A160WE LEDs utilised on maximum intensity and colour settings) could be identified through this titration experiment utilising up to 4.0 equiv. of iodopentafluorobenzene.

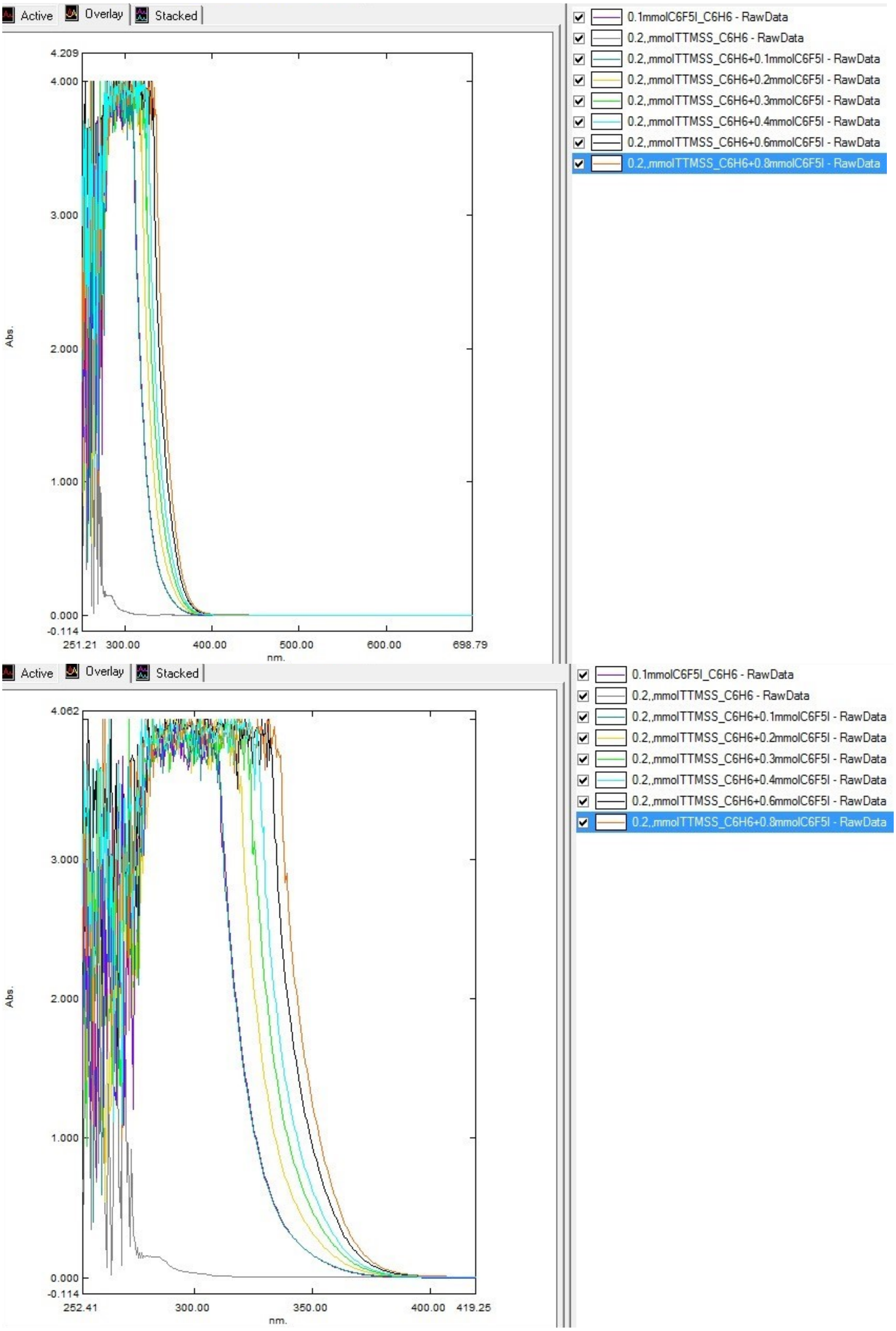

Figure S5. (a) Overlay of UV-Vis spectroscopy titration study of iodopentafluorobenzene into TTMSS. **(b)** Expanded region of the overlay highlighting a red-shift in absorbance of the mixture as the concentration of iodopentafluorobenzene is increased.

In order to validify that the red-shift observed in the previous titration experiment was undoubtedly the result of an interaction between the halide and TTMSS, a control experiment was conducted whereby the UV-Vis spectrum of increasing concentrations of iodopentafluorobenzene were recorded.

To a 1.0 mL UV quartz cuvette with a lid was added benzene (1.0 mL), and a reference spectrum taken. Iodopentafluorobenzene (6.5 µL, 5.0 x 10^{-2} mmol mmol) was added, the cuvette vigorously shaken, and a measurement recorded. This addition sequence was repeated once to provide a total of 0.1 mmol of iodopentafluorobenzene. Subsequently, iodopentafluorobenzene (13.0 µL, 0.1 mmol) was added to provide a total of 0.2 mmol. the mixture was vigorously shaken, and a spectrum measured. This addition sequence was repeated a further two times to afford a mixture containing 0.4 mmol iodopentafluorobenzene. The spectra recorded from this experiment are overlaid in Figure S6.

As the concentration of iodopentafluorobenzene was increased, a continual red-shift in the absorbance of the solution was observed. Consequently, the trajectory identified in the previous titration experiment could be attributed to alterations in the media environment, rather than a representation of an interaction between the halide and TTMSS.

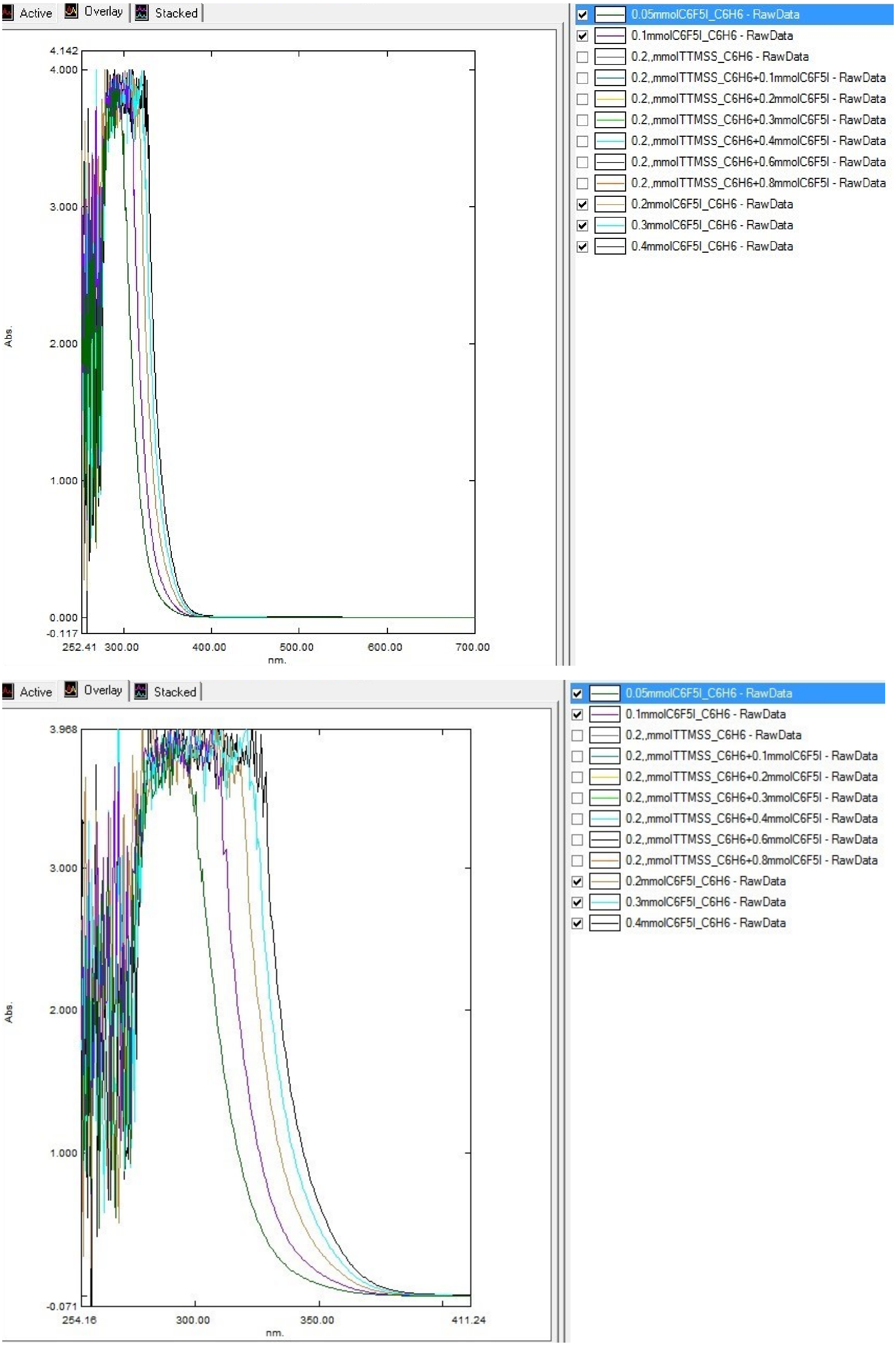

Figure S6. (a) Overlay of UV-Vis spectra recorded as the concentration of iodopentafluorobenzene was increased. **(b)** Expanded region of the overlay highlighting a red-shift in the absorbance.

Flow-IR spectroscopy titration studies

A potential interaction between TTMSS and a halocarbon resulting in initiation and radical formation could alter the lengths and strengths of the Si–H and C–X bonds, and so their IR stretching frequencies as well. Consequently, the distinct band representing Si–H at 2052 cm^{-1} could be monitored as the concentration of the halogen component was increased.[15] In order to maximise any halogen bonding effects as per previous titration experiments, iodopentafluorobenzene was chosen as the partner and benzene as the solvent. A recirculating flow setup was utilised to provide continuous analysis of the mixture, thereby facilitating detection of subtle modifications in peaks throughout the titration experiment.

To an unsealed, tapered Biotage glass microwave reaction vial (0.5-2.0 mL) was added benzene (1.0 mL). Inlet and outlet PTFE tubing flowing through a benchtop IR spectrometer were inserted, and recirculation of the solution was started at a rate of 0.1 mL min^{-1} to provide a background reference spectrum. TTMSS (60.0 µL, 0.2 mmol, 1.0 equiv.) was added, and the mixture was recirculated for 5 min. before iodopentafluorobenzene (13.0 µL, 0.1 mmol, 0.5 equiv.) was charged in 10 min. increments up to 2.0 equiv. total. After flowing for a further 10 min., an increased loading of iodopentafluorobenzene (26.0 µL, 0.2 mmol, 1.0 equiv., 3.0 equiv. total) was applied. The system was equilibrated for 10 min., and then a final portion of iodopentafluorobenzene (26.0 µL, 0.2 mmol, 1.0 equiv., 5.0 equiv. total) was added. This mixture was recirculated for a further 45 min. before termination of the experiment. A 3-D plot of the continuous IR spectra generated throughout this sequence is displayed in Figure S7.

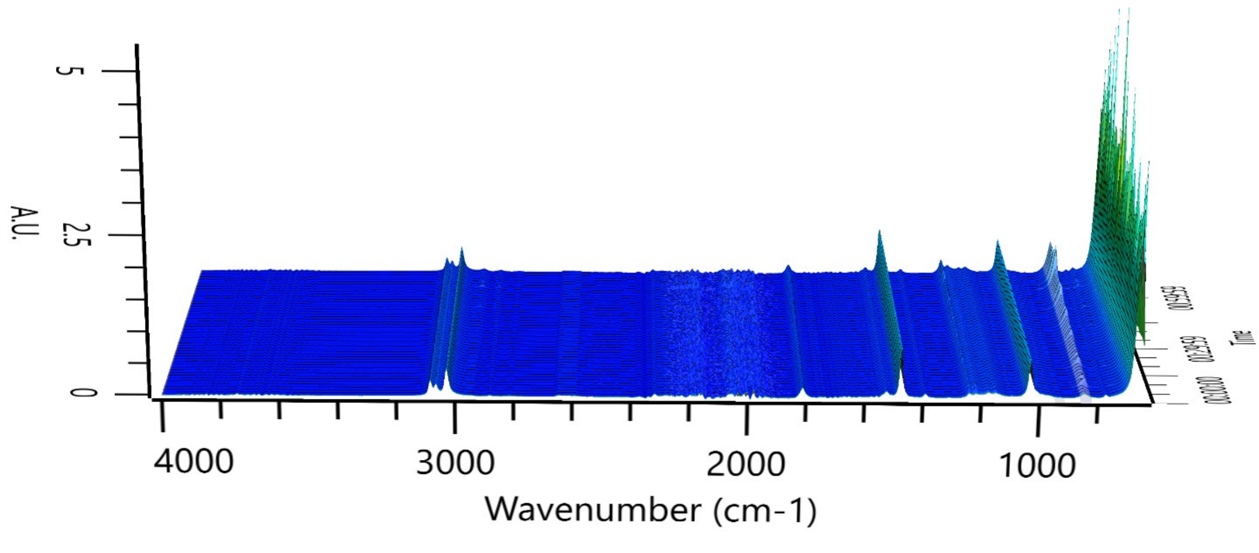

Figure S7. A 3-D plot of the continuous IR spectra generated through the titration experiment.

As exemplified, no distinct shifts in the peaks present in the IR spectrum was observed, nor the appearance of additional bands which could be attributed to an interaction between TTMSS and iodopentafluorobenzene.

Spectroscopic titration studies conclusion

Overall, whilst our experimental results outlined in Table 1 suggest the presence of a unique interaction between TTMSS and haloalkanes which afford visible light-mediated initiation and radical formation, all titration studies outlined herein were inconclusive. We hypothesise that, regardless of the nature of this interaction, it may be present in concentrations under the detection limits of the spectroscopic methods attempted; alternatively, it could possess a transient lifetime that is shorter than the timescale of the spectroscopic experiments we have run, thereby evading detection and observation.

References

(1) Dao, H. T., Li, C., Michaudel, Q., Maxwell, B. D., Baran, P. S. *J. Am. Chem. Soc.* **2015**, *137*, 8046–8049.

(2) Manley, D. W., McBurney, R. T., Miller, P., Walton, J. C., Mills, A., O'Rourke, C. *J. Org. Chem.* **2014**, *79*, 1386–1398.

(3) Ackerman, L. K. G., Martinez Alvarado, J. I., Doyle, A. G. *J. Am. Chem. Soc.* **2018**, *140*, 14059–14063.

(4) Adeyemi, A., Bergman, J., Brånalt, J., Sävmarker, J., Larhed, M. *Org. Process Res. Dev.* **2017**, *21*, 947–955.

(5) Russell, G. A., Shi, B. Z., Jiang, W., Hu, S., Kim, B. H., Baik, W. *J. Am. Chem. Soc.* **1995**, *117*, 3952–3962.

(6) Jin, Y., Yang, H., Fu, H. *Org. Lett.* **2016**, *18*, 6400–640

(7) Yan, Y., Zhang, Z., Wan, Y., Zhang, G., Ma, N., Liu, Q. *J. Org. Chem.* **2017**, *82*, 7957–7963.

(8) Cavallo, G., Metrangolo, P., Milani, R., Pilati, T., Priimagi, A., Resnati, G., Terraneo, G. *Chem. Rev.* **2016**, *116*, 2478–2601, and references therein

(9) Politzer, P., Lane, P., Concha, M. C., Ma, Y., Murray, J. S. *J. Mol. Model.* **2007**, *13*, 305–311

(10) (a) Van Dyke Tiers, G. *J. Fluor. Chem.* **1999**, *98*, 89–95; (b) Van Dyke Tiers, G. *J. Fluor. Chem.* **2000**, *102*, 175–184

(11) Glaser, R., Chen, N., Wu, H., Knotts, N., Kaupp, M. *J. Am. Chem. Soc.* **2004**, *126*, 4412–4419

(12) (a) Präsang, C., Whitwood, A. C., Bruce, D. W. *Cryst. Growth Des.*, **2009**, *9*, 5319–5326; (b) Riley, K. E., Murray, J. S., Fanfrlík, J., Řezáč, J., Solá, R. J., Concha, M. C., Ramos, F. M., Politzer, P. *J. Mol. Model.* **2011**, *17*, 3309–3318

(13) Gilman, H., Atwell, W. H. Sen, P. K. Smith, C. L. *J. Organomet. Chem.* **1965**, *4*, 163–167

(14) Renaud, J., Scaiano, J. C. *Can. J. Chem.* **1996**, *74*, 1724–1730

(15) Boo, B. H., Kang, H. K., Kang, S. K., Lee, S. S., Kim, D., Lee, M. H. *J. Organomet. Chem.* **1992**, *436*, 1–9.

^{1}H and ^{13}C Spectra

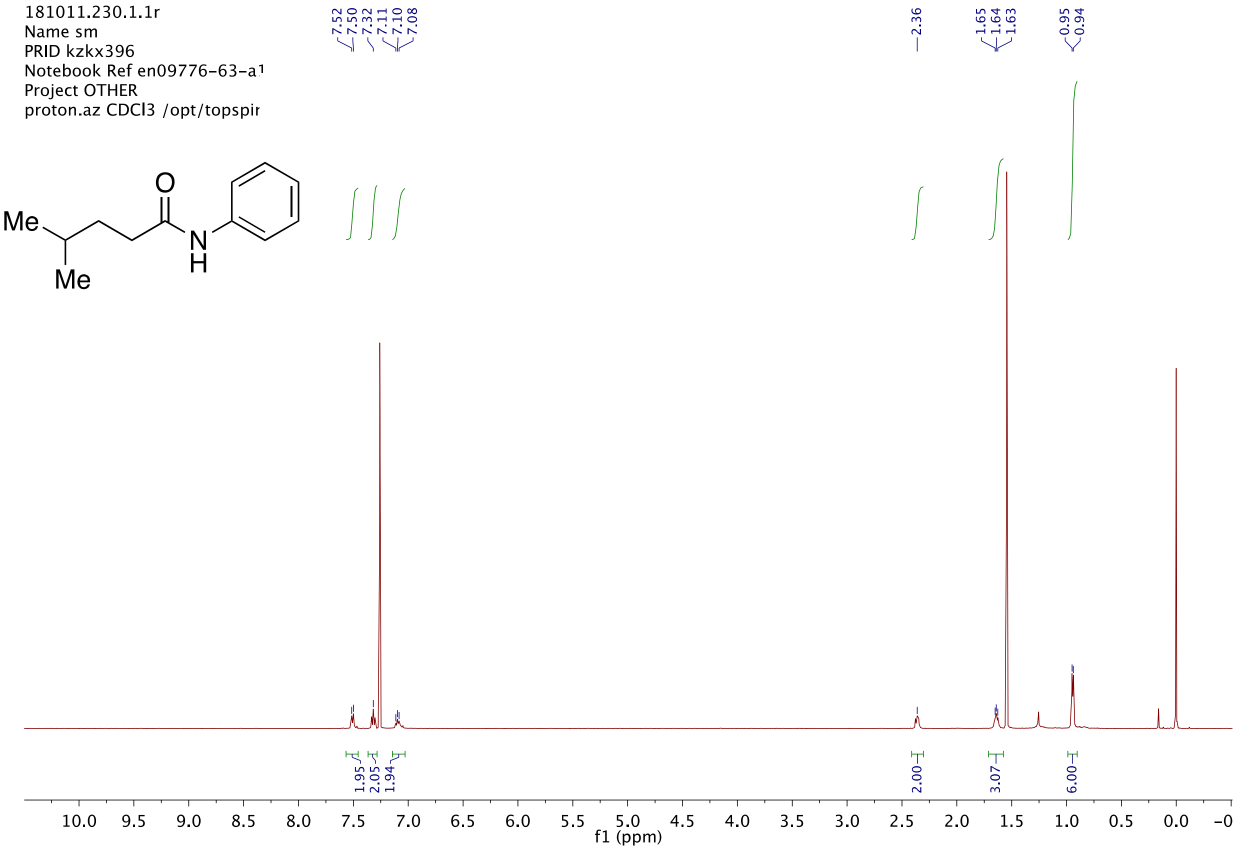

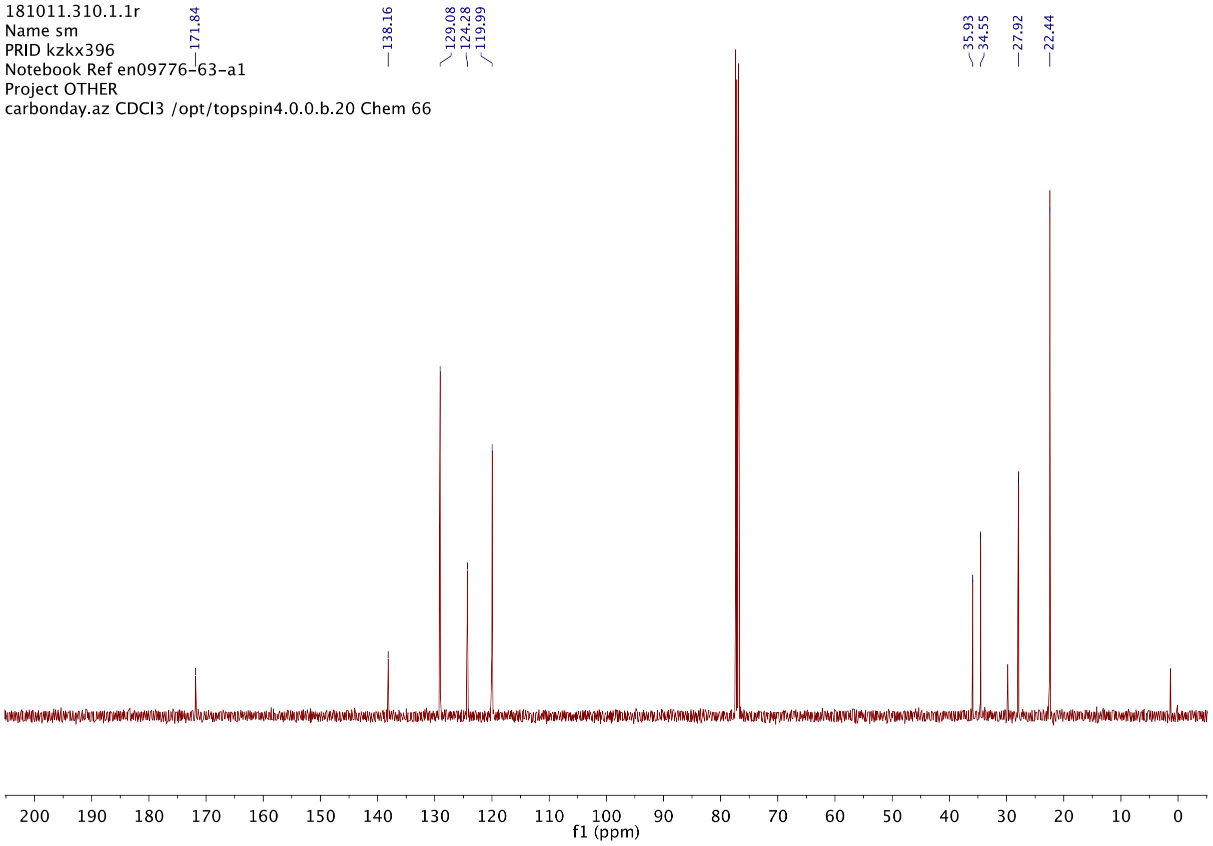

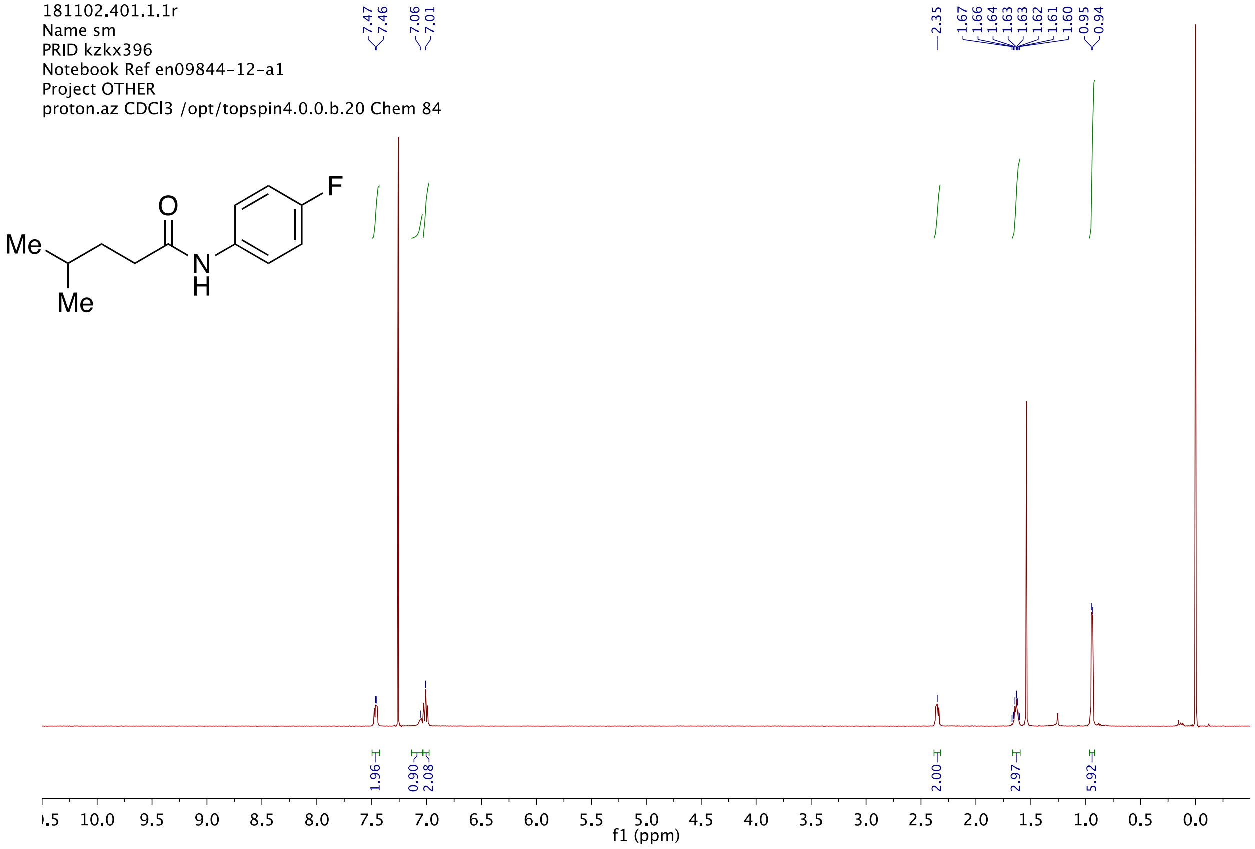
181102.401.1.1r
Name sm
PRID kzkx396
Notebook Ref en09844-12-a1
Project OTHER
proton.az CDCl3 /opt/topspin4.0.0.b.20 Chem 84
Me
Me
O
N
H
F
7.47
7.46
7.06
7.01
2.35
1.67
1.66
1.64
1.63
1.63
1.62
1.61
1.60
0.95
0.94
1.96
0.90
2.08
2.00
2.97
5.92
f1 (ppm)

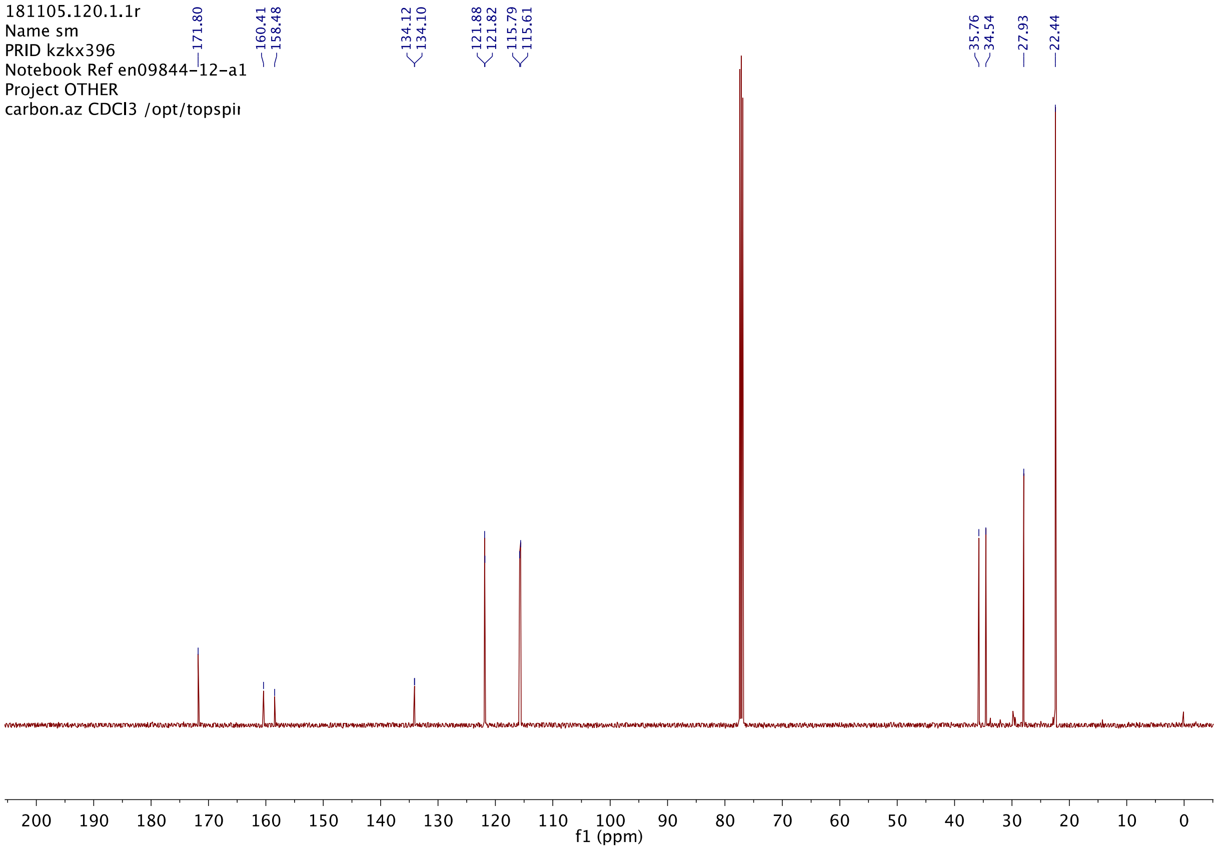
181105.120.1.1r
Name sm
PRID kzkx396
Notebook Ref en09844-12-a1
Project OTHER
carbon.az CDCl3 /opt/topspi
171.80
160.41
158.48
134.12
134.10
121.88
121.82
115.79
115.61
35.76
34.54
27.93
22.44
f1 (ppm)

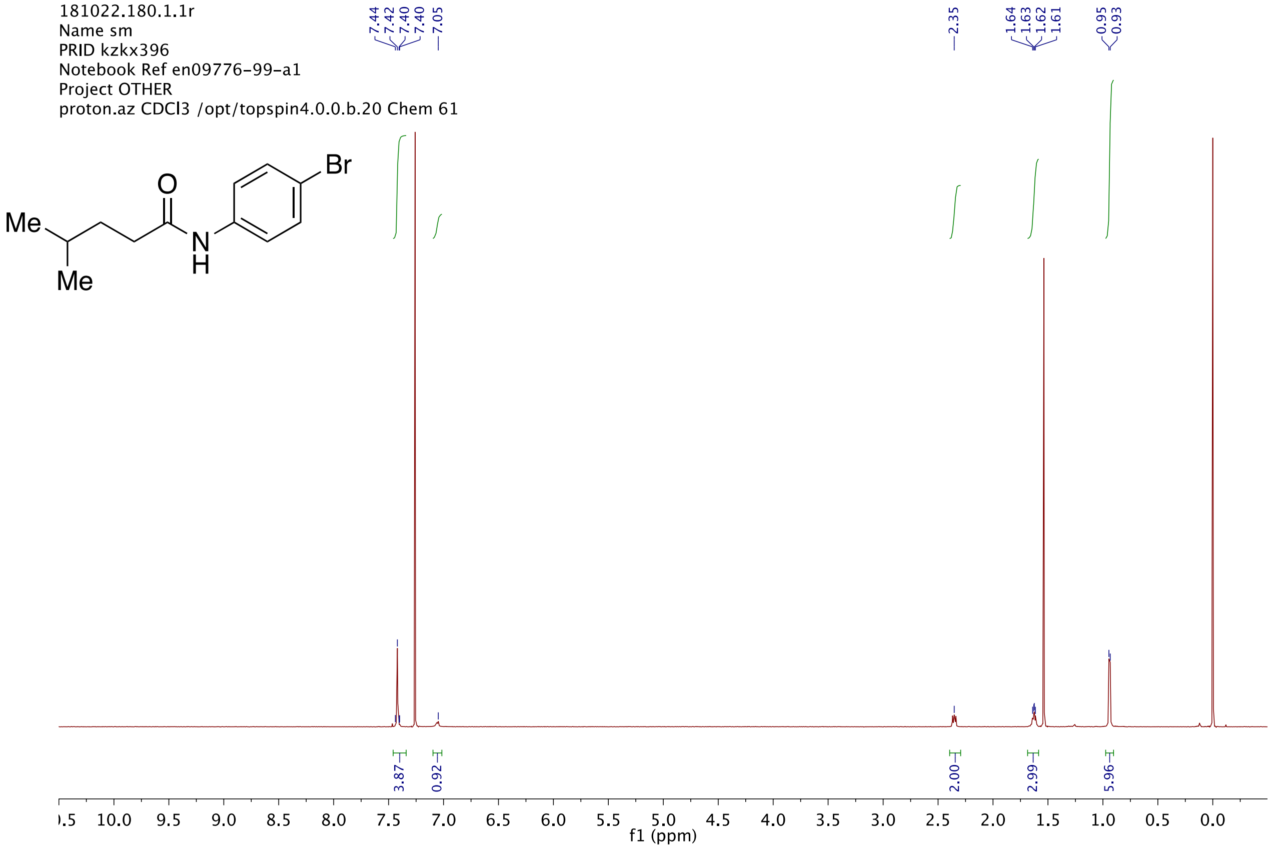
181022.180.1.1r
Name sm
PRID kzkx396
Notebook Ref en09776-99-a1
Project OTHER
proton.az CDCl3 /opt/topspin4.0.0.b.20 Chem 61
7.44
7.42
7.40
7.40
7.05
2.35
1.64
1.63
1.62
1.61
0.95
0.93
Br
O
Me
N
H
Me
3.87
0.92
2.00
2.99
5.96
10.0 9.5 9.0 8.5 8.0 7.5 7.0 6.5 6.0 5.5 5.0 4.5 4.0 3.5 3.0 2.5 2.0 1.5 1.0 0.5 0.0
f1 (ppm)

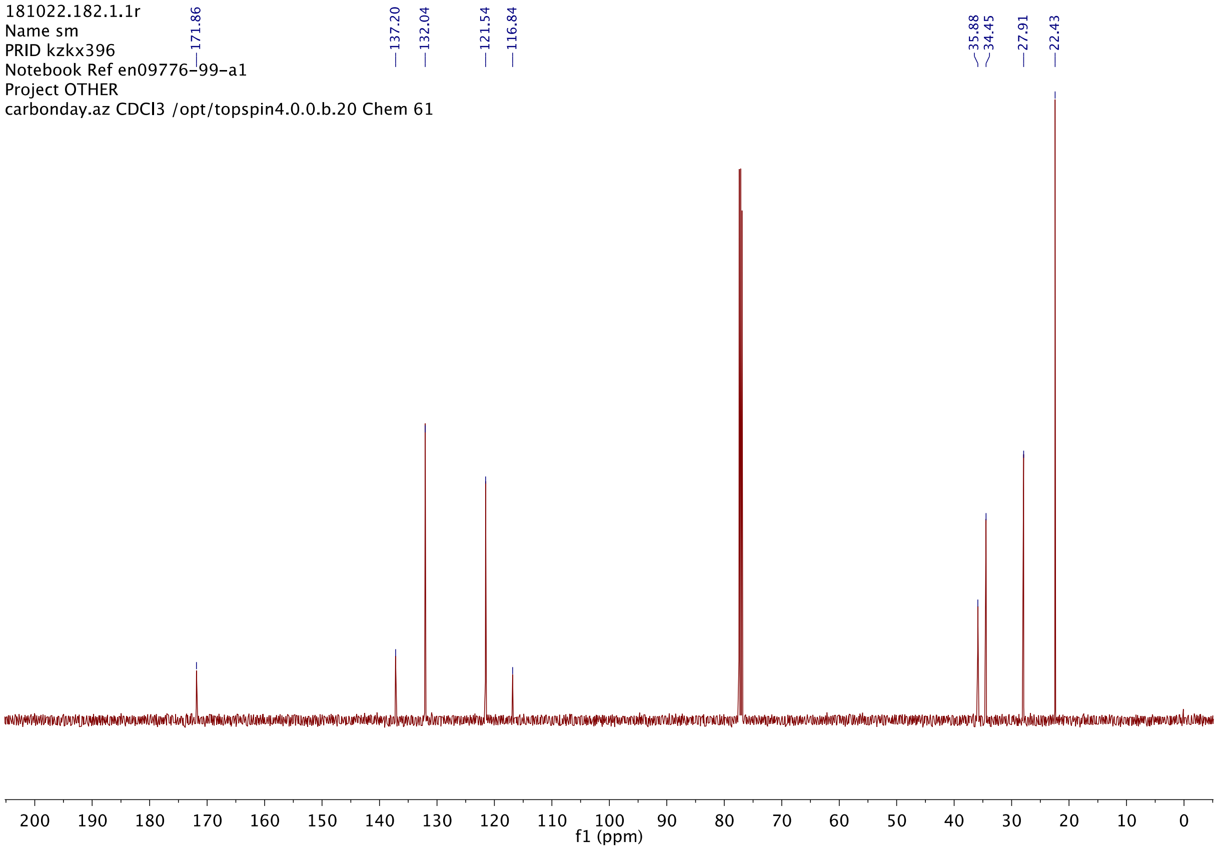
181022.182.1.1r
Name sm
PRID kzkx396
Notebook Ref en09776-99-a1
Project OTHER
carbonday.az CDCl3 /opt/topspin4.0.0.b.20 Chem 61
171.86
137.20
132.04
121.54
116.84
35.88
34.45
27.91
22.43
200 190 180 170 160 150 140 130 120 110 100 90 80 70 60 50 40 30 20 10 0
f1 (ppm)

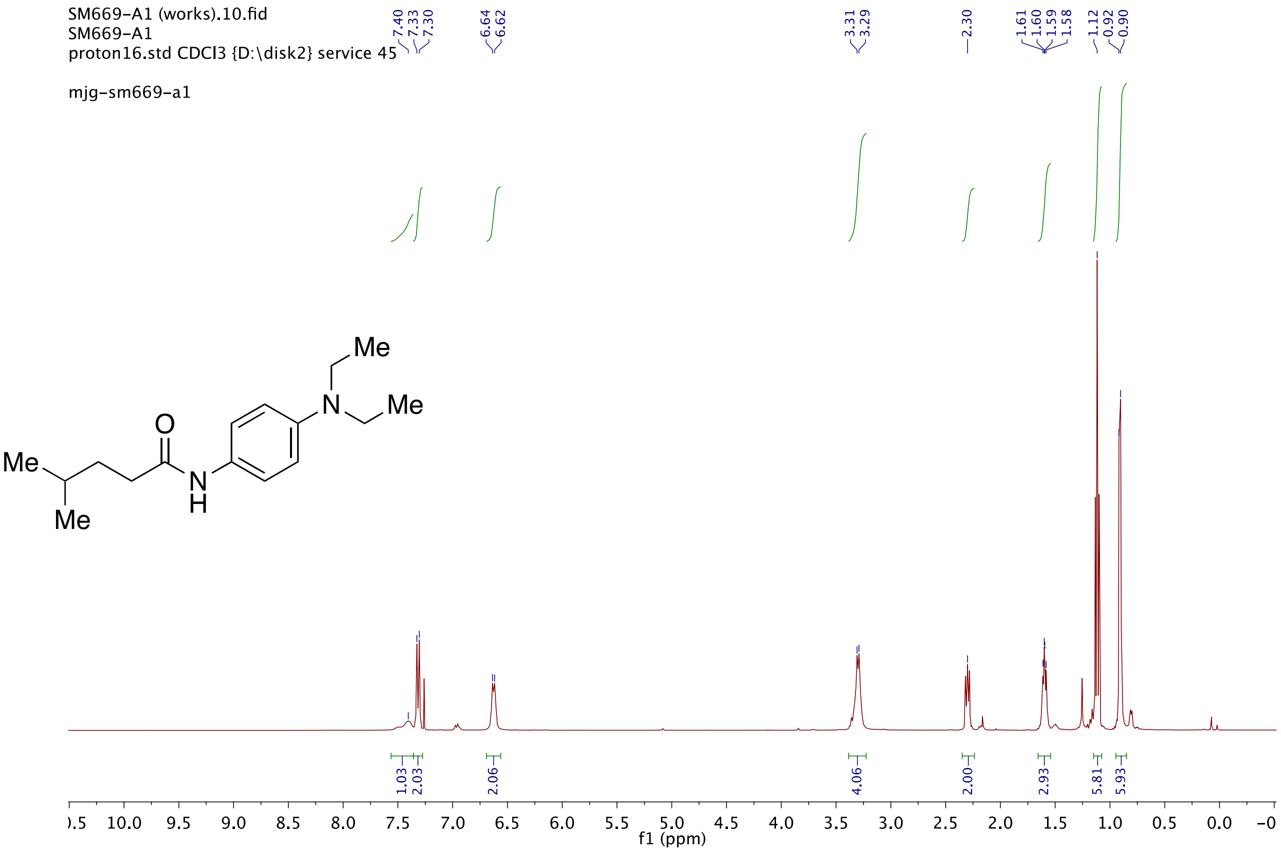
SM669-A1 (works).10.fid
SM669-A1
proton16.std CDCl3 {D:\disk2} service 45
mjg-sm669-a1
7.40
7.33
7.30
6.64
6.62
3.31
3.29
2.30
1.61
1.60
1.59
1.58
1.12
0.92
0.90
Me
N
Me
O
Me
N
H
Me
1.03
2.03
2.06
4.06
2.00
2.93
5.81
5.93
f1 (ppm)

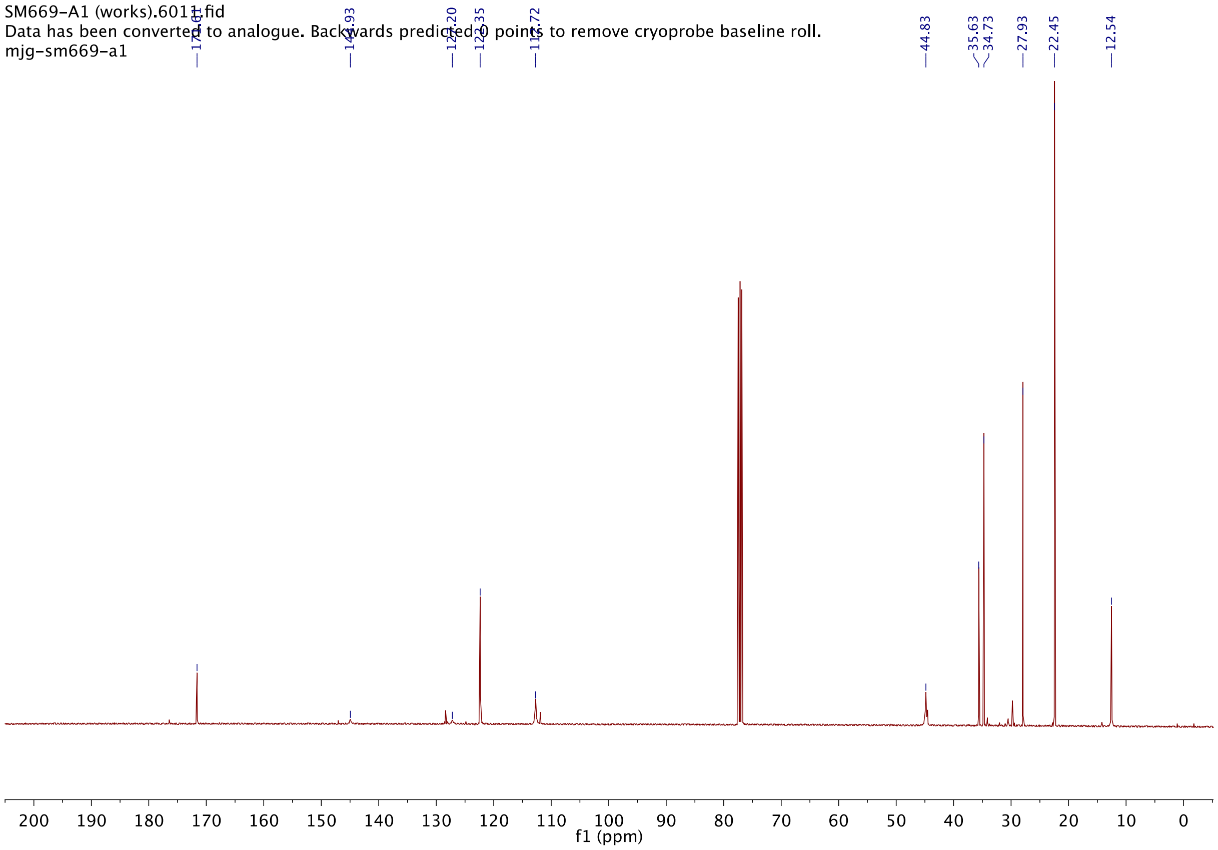
SM669-A1 (works).6011.fid
Data has been converted to analogue. Backwards predicted 0 points to remove cryoprobe baseline roll.
mjg-sm669-a1
44.83
35.63
34.73
27.93
22.45
12.54
f1 (ppm)

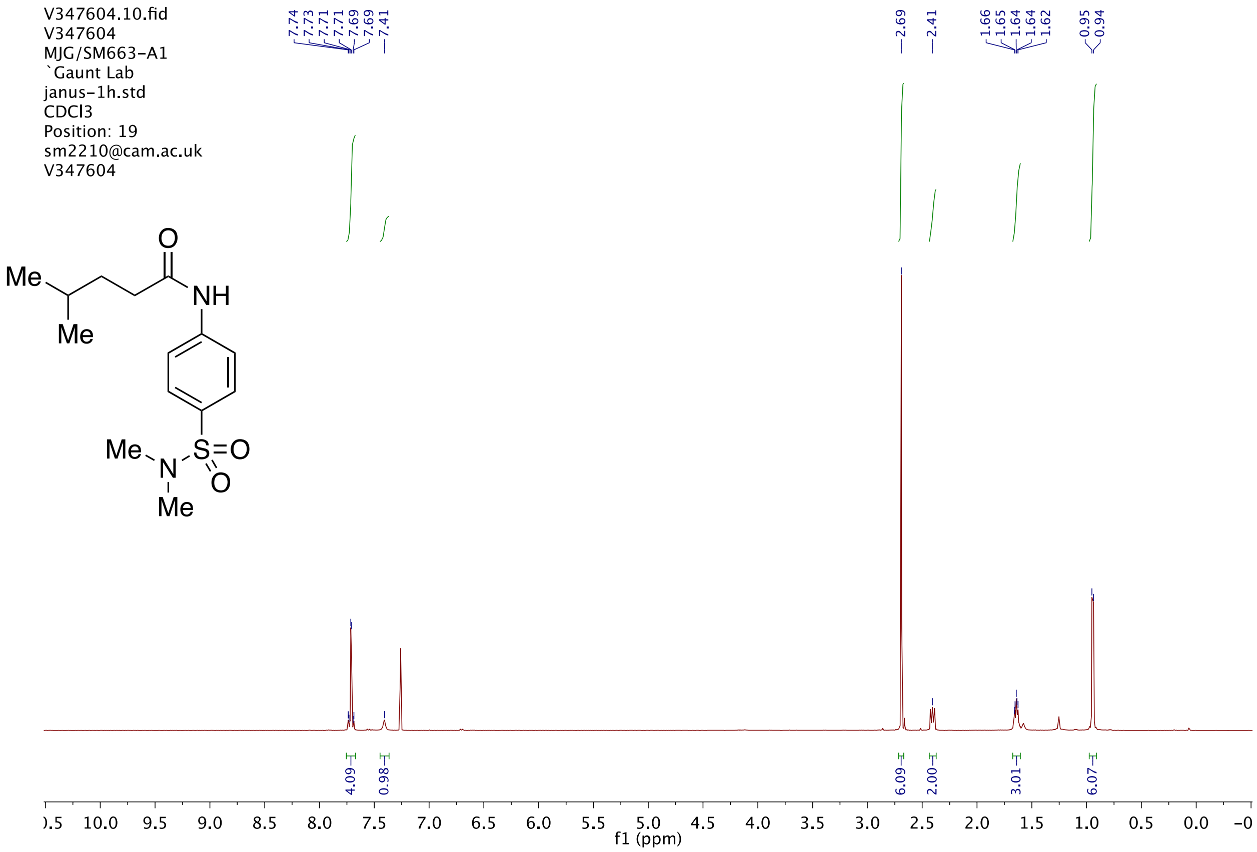
V347604.10.fid
V347604
MJG/SM663-A1
`Gaunt Lab
janus-1h.std
CDCl3
Position: 19
sm2210@cam.ac.uk
V347604
7.74
7.73
7.71
7.71
7.69
7.69
7.41
2.69
2.41
1.66
1.65
1.64
1.64
1.62
0.95
0.94
Me
Me
O
NH
Me
N
S
O
O
Me
4.09
0.98
6.09
2.00
3.01
6.07
f1 (ppm)

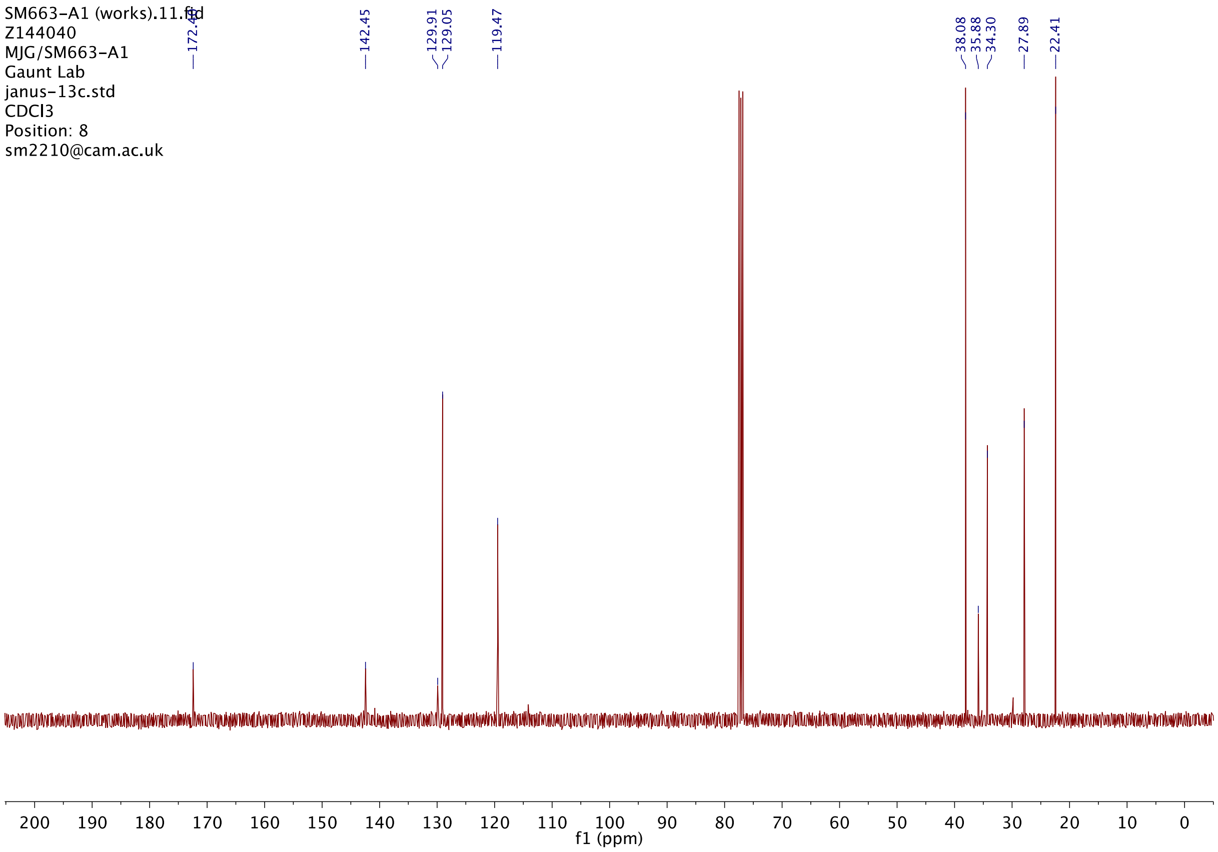
SM663-A1 (works).11.fid
Z144040
MJG/SM663-A1
Gaunt Lab
janus-13c.std
CDCl3
Position: 8
sm2210@cam.ac.uk
172.40
142.45
129.91
129.05
119.47
38.08
35.88
34.30
27.89
22.41
f1 (ppm)

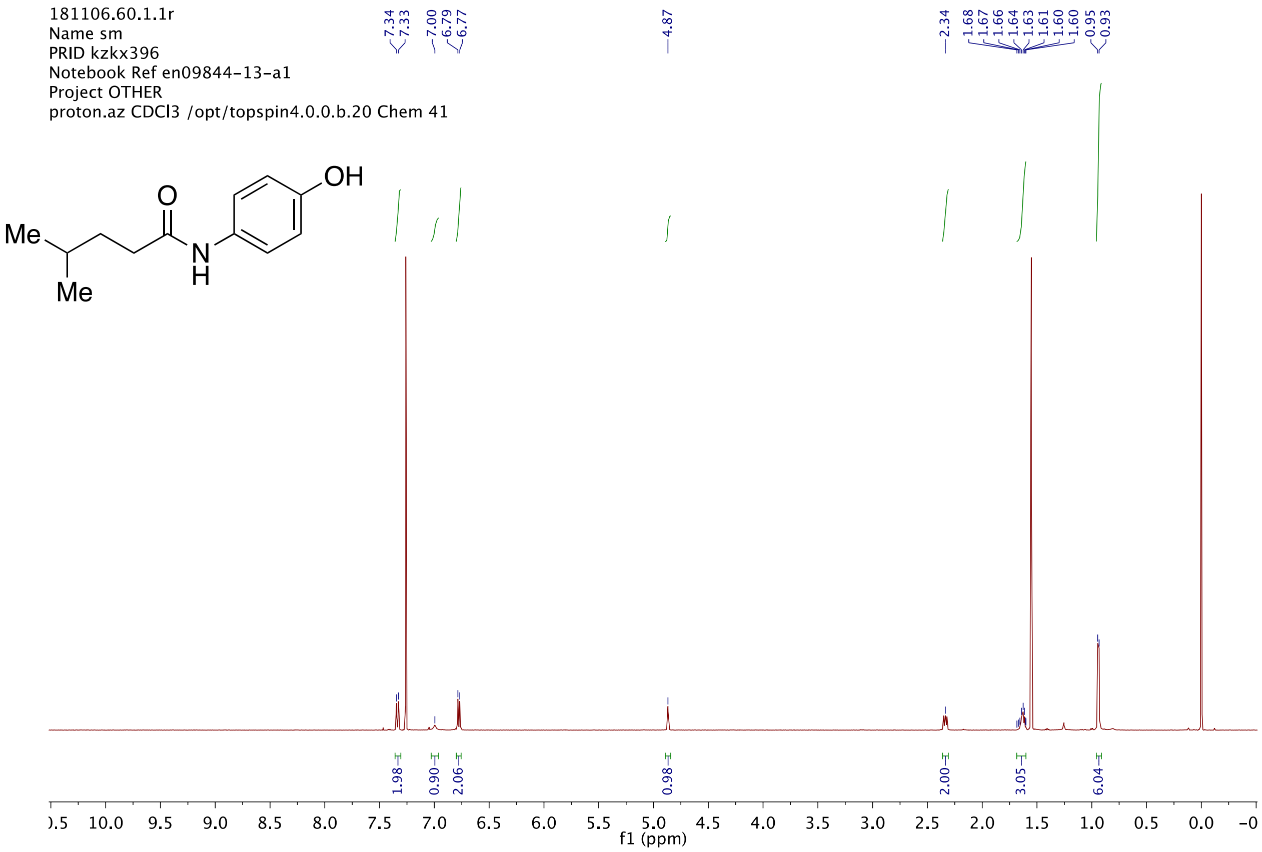
181106.60.1.1r
Name sm
PRID kzkx396
Notebook Ref en09844-13-a1
Project OTHER
proton.az CDCl3 /opt/topspin4.0.0.b.20 Chem 41
7.34
7.33
7.00
6.79
6.77
4.87
2.34
1.68
1.67
1.66
1.64
1.63
1.61
1.60
1.60
0.95
0.93
Me
Me
O
N
H
OH
1.98
0.90
2.06
0.98
2.00
3.05
6.04
f1 (ppm)

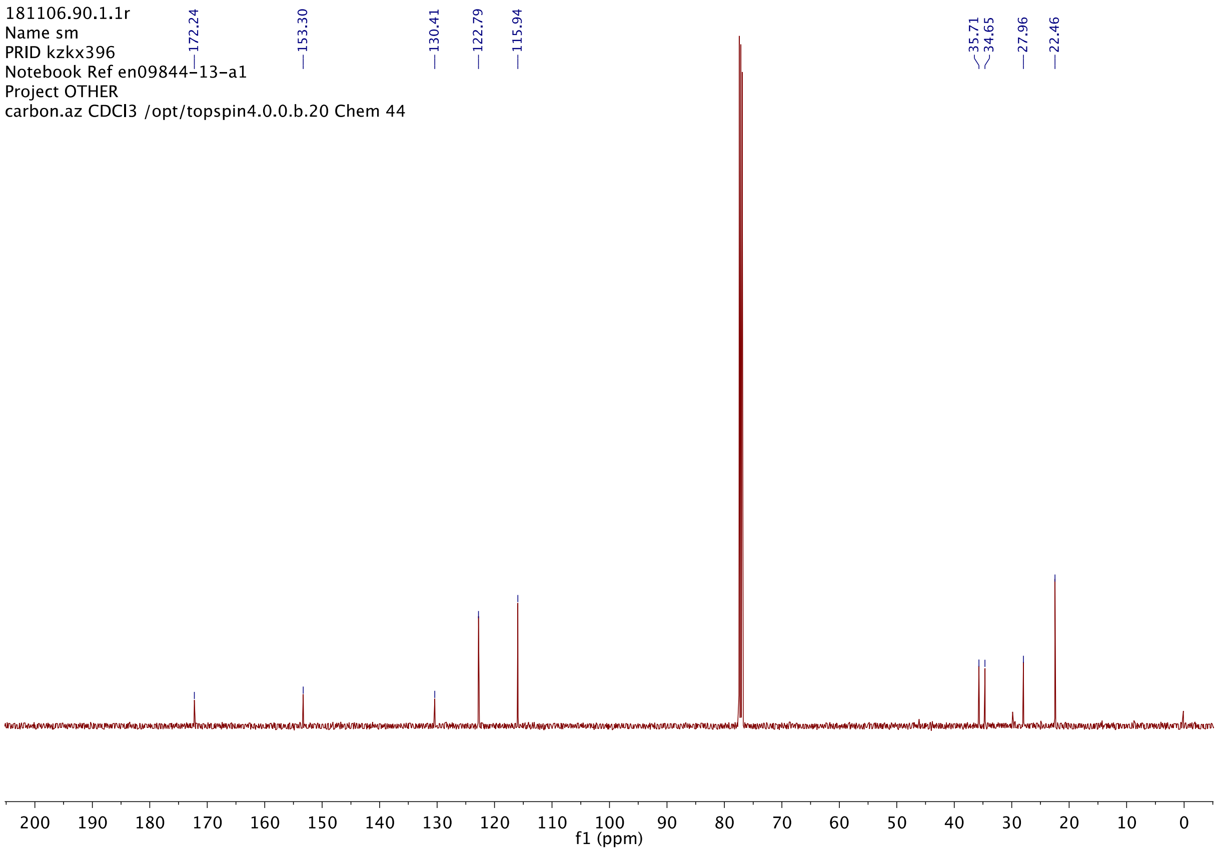
181106.90.1.1r
Name sm
PRID kzkx396
Notebook Ref en09844-13-a1
Project OTHER
carbon.az CDCl3 /opt/topspin4.0.0.b.20 Chem 44
172.24
153.30
130.41
122.79
115.94
35.71
34.65
27.96
22.46
f1 (ppm)

181026.260.1.1r
Name sm
PRID KZKX396
Notebook Ref EN09844-07-A1
Project OTHER
proton.az CDCl3 /opt/topspin4.0.0.b.20 Chem 5

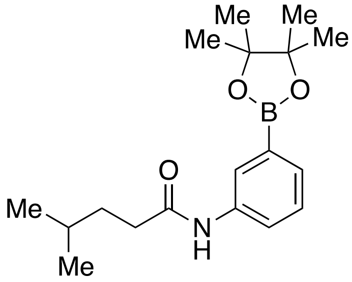

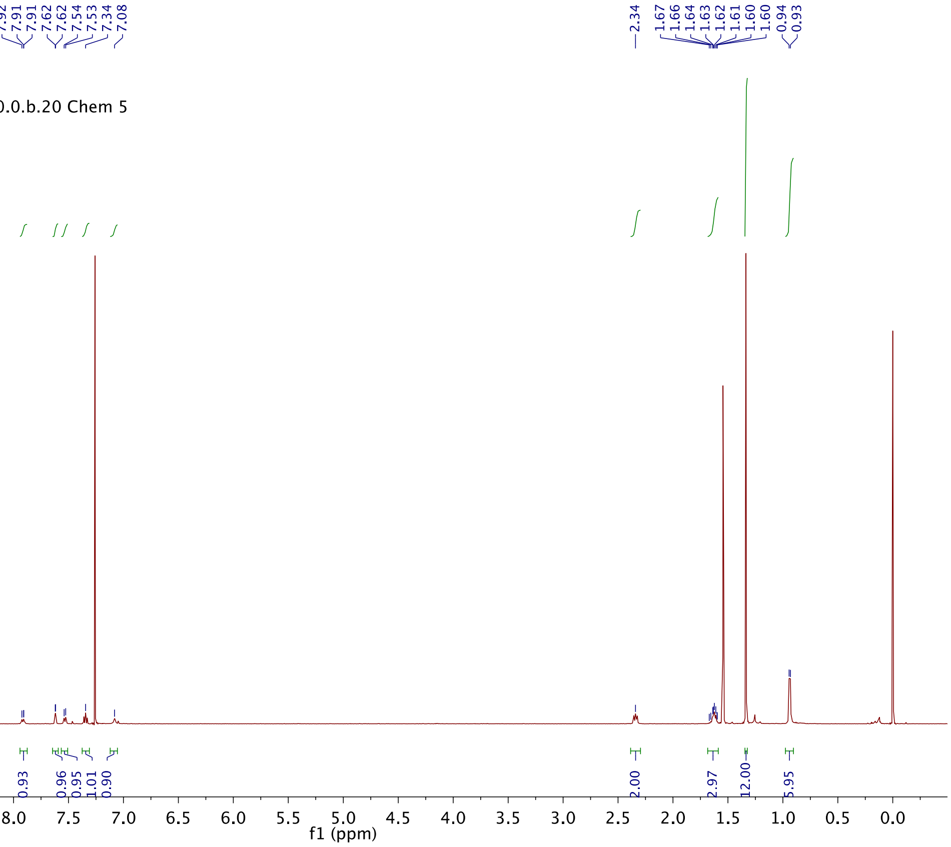

181031.90.1.1r
Name sm
PRID kzkx396
Notebook Ref en09844-07-a1
Project OTHER
carbonday.az CDCl3 /opt/topspin4.0.0.b.20 Chem 72

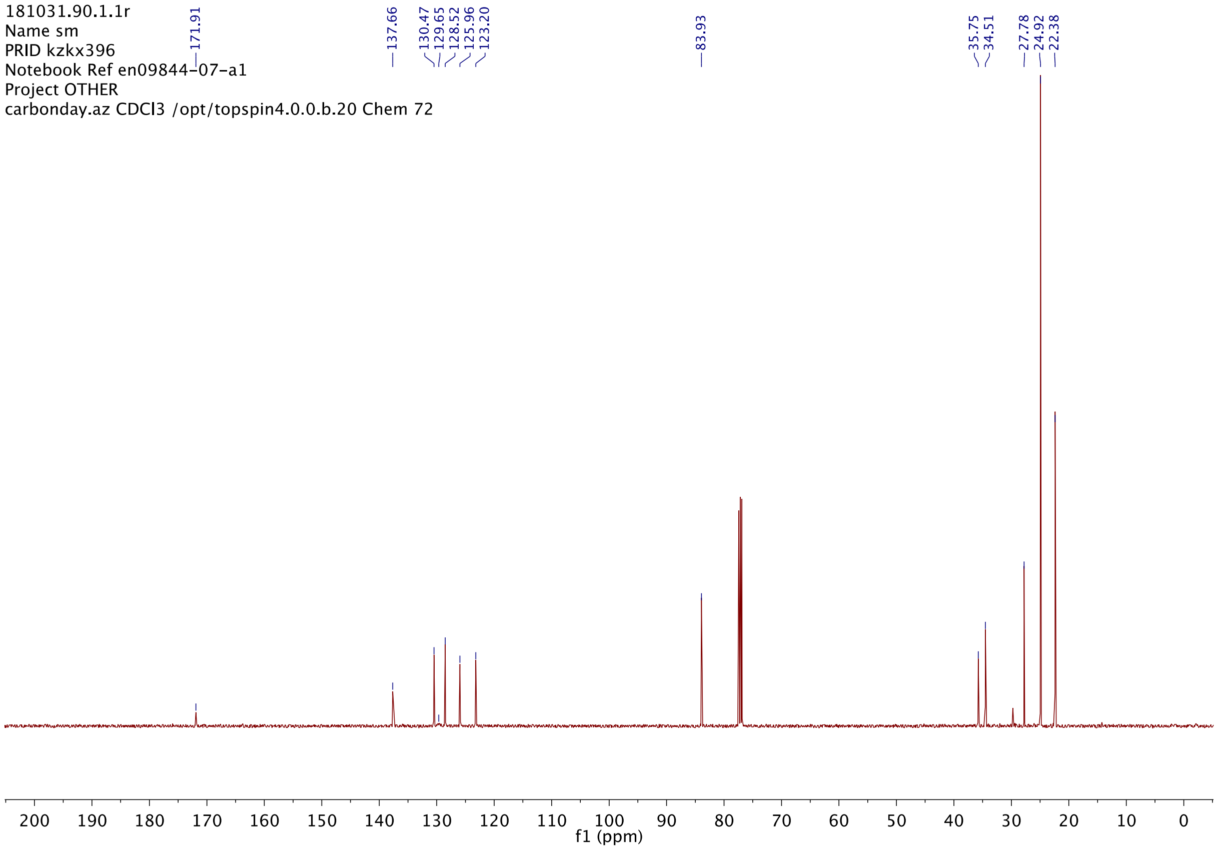

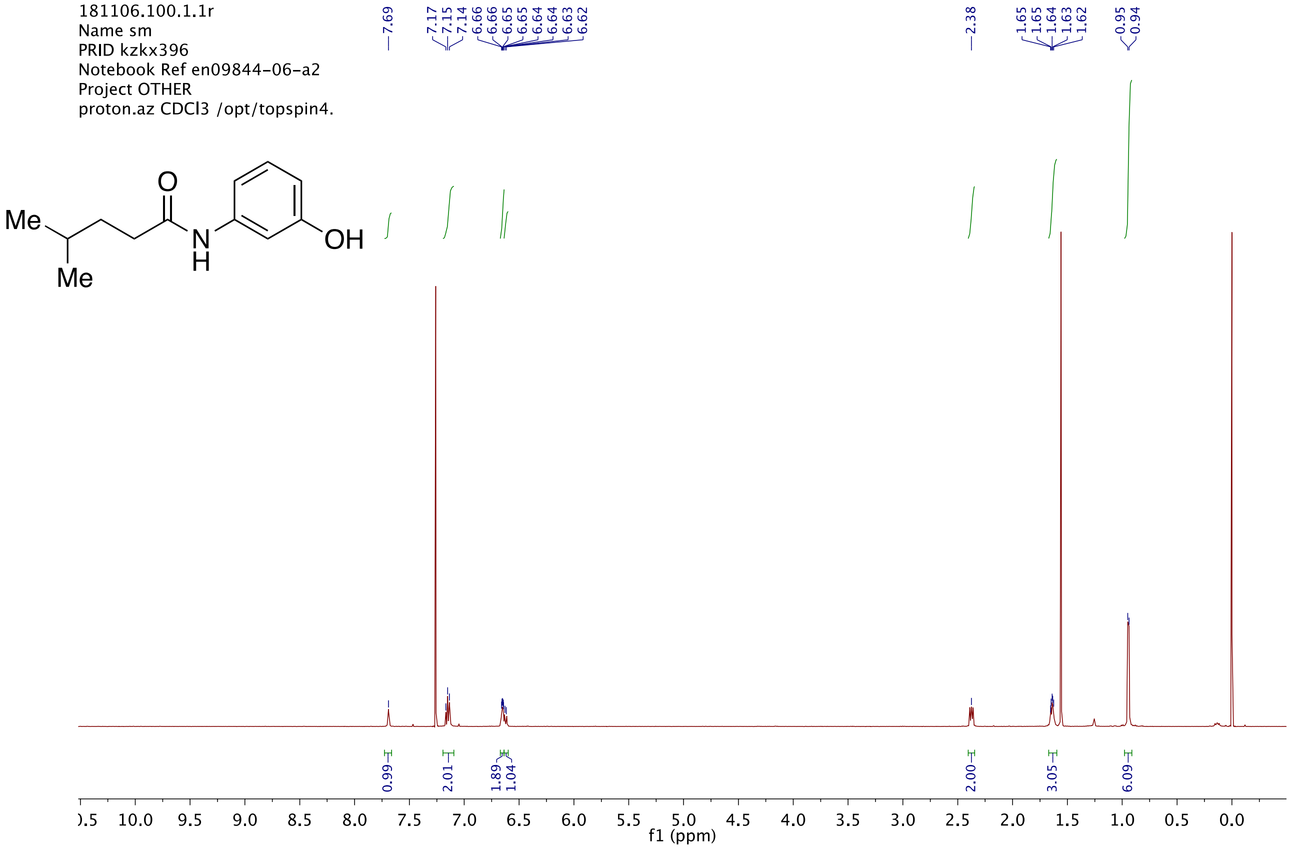
181106.100.1.1r
Name sm
PRID kzkx396
Notebook Ref en09844-06-a2
Project OTHER
proton.az CDCl3 /opt/topspin4.
Me
Me
O
N
H
OH
7.69
7.17
7.15
7.14
6.66
6.66
6.65
6.65
6.64
6.64
6.63
6.62
2.38
1.65
1.65
1.64
1.63
1.62
0.95
0.94
0.99
2.01
1.89
1.04
2.00
3.05
6.09
f1 (ppm)

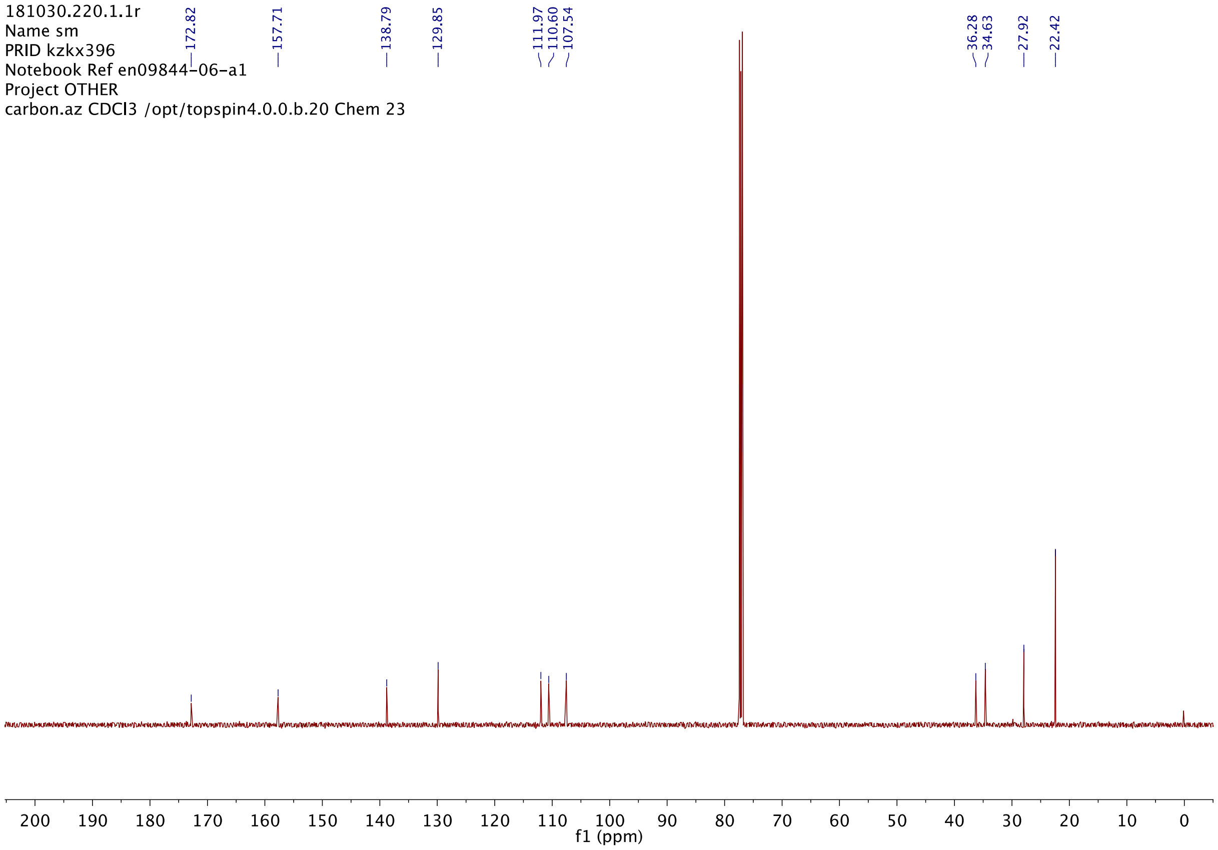
181030.220.1.1r
Name sm
PRID kzkx396
Notebook Ref en09844-06-a1
Project OTHER
carbon.az CDCl3 /opt/topspin4.0.0.b.20 Chem 23
172.82
157.71
138.79
129.85
111.97
110.60
107.54
36.28
34.63
27.92
22.42
f1 (ppm)

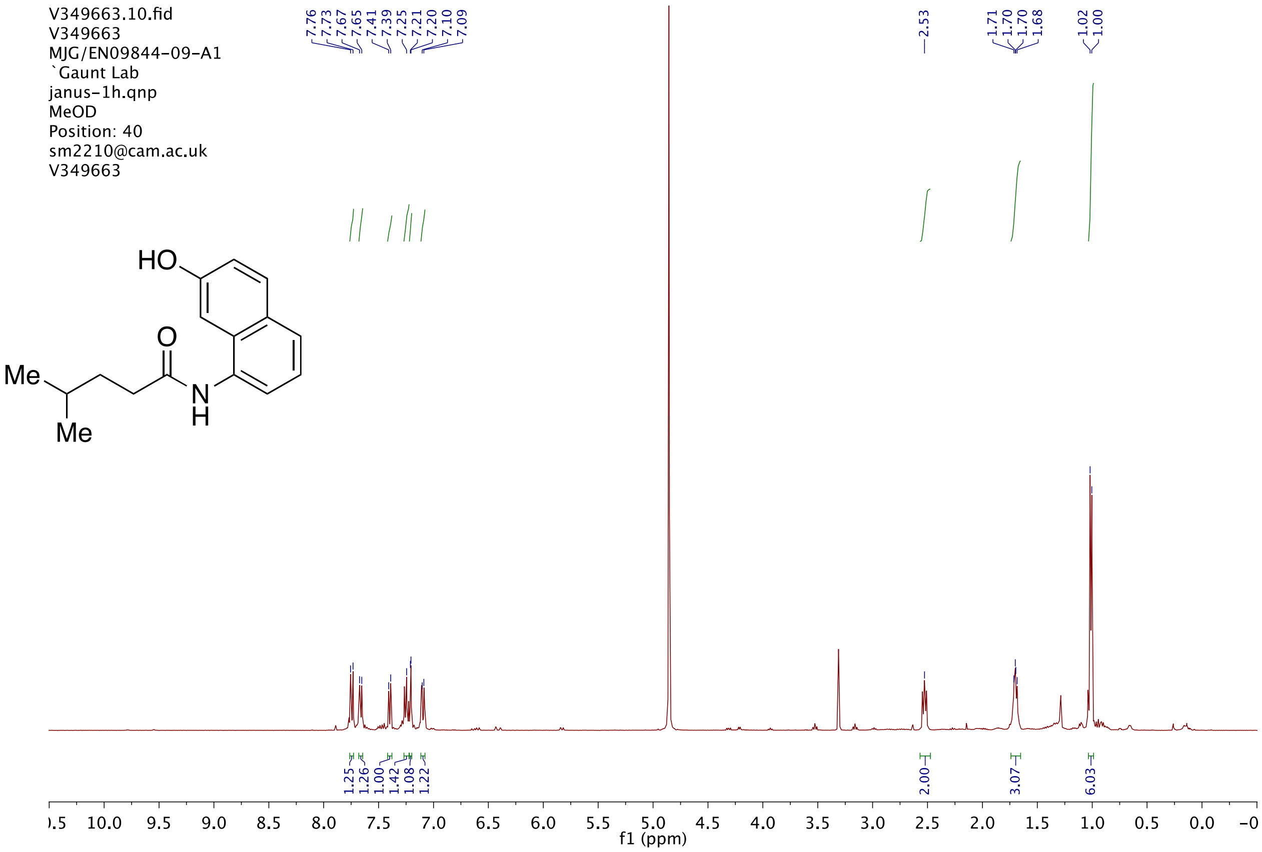
V349663.10.fid
V349663
MJG/EN09844-09-A1
`Gaunt Lab
janus-1h.qnp
MeOD
Position: 40
sm2210@cam.ac.uk
V349663
7.76
7.73
7.67
7.65
7.41
7.39
7.25
7.21
7.20
7.10
7.09
2.53
1.71
1.70
1.70
1.68
1.02
1.00
HO
O
Me
Me
N
H
1.25
1.26
1.00
1.42
1.08
1.22
2.00
3.07
6.03
f1 (ppm)

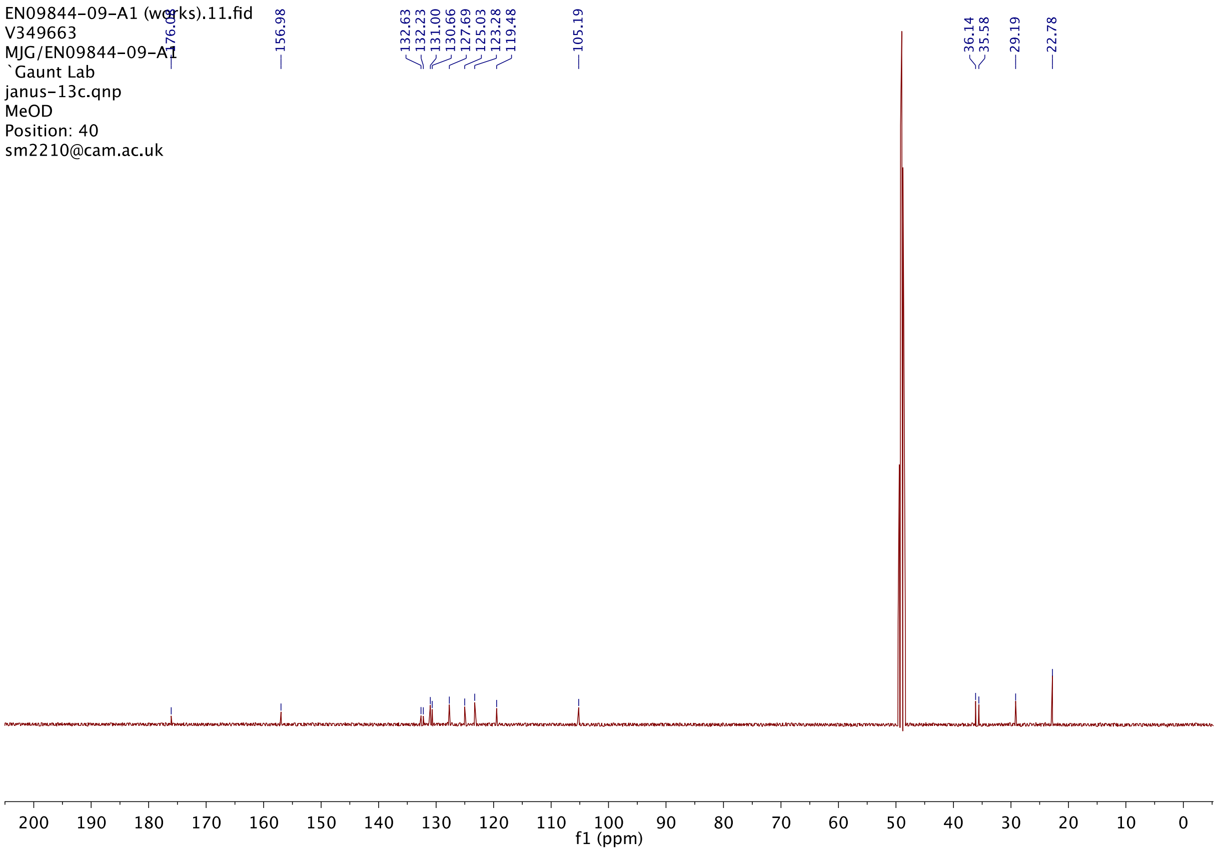
EN09844-09-A1 (works).11.fid
V349663
MJG/EN09844-09-A1
`Gaunt Lab
janus-13c.qnp
MeOD
Position: 40
sm2210@cam.ac.uk
156.98
132.63
132.23
131.00
130.66
127.69
125.03
123.28
119.48
105.19
36.14
35.58
29.19
22.78
f1 (ppm)

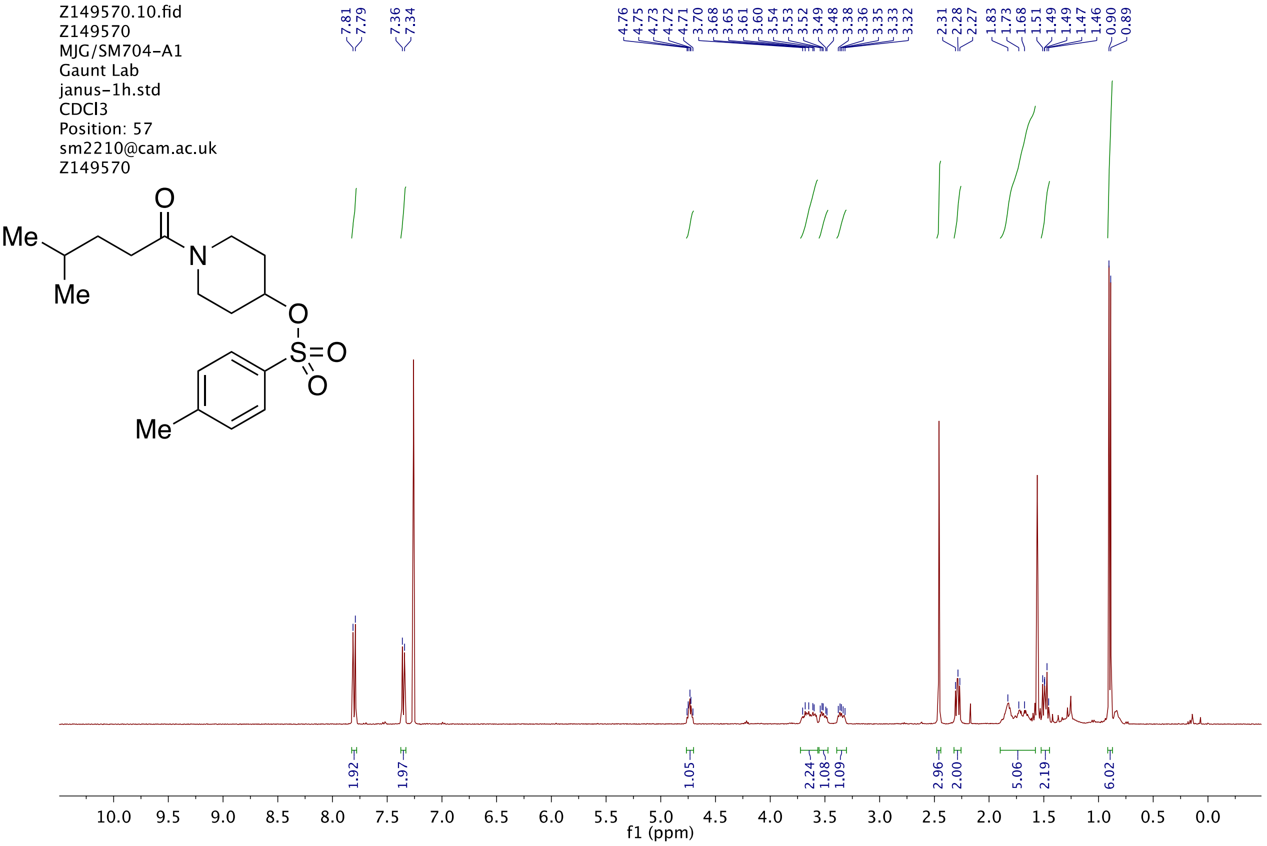
Z149570.10.fid
Z149570
MJG/SM704-A1
Gaunt Lab
janus-1h.std
CDCl3
Position: 57
sm2210@cam.ac.uk
Z149570
Me
Me
O
N
O
S=O
O
Me
7.81
7.79
7.36
7.34
4.76
4.75
4.73
4.72
4.71
3.70
3.68
3.65
3.61
3.60
3.54
3.53
3.52
3.49
3.48
3.38
3.36
3.35
3.33
3.32
2.31
2.28
2.27
1.83
1.73
1.68
1.51
1.49
1.49
1.47
1.46
0.90
0.89
1.92
1.97
1.05
2.24
1.08
1.09
2.96
2.00
5.06
2.19
6.02
f1 (ppm)

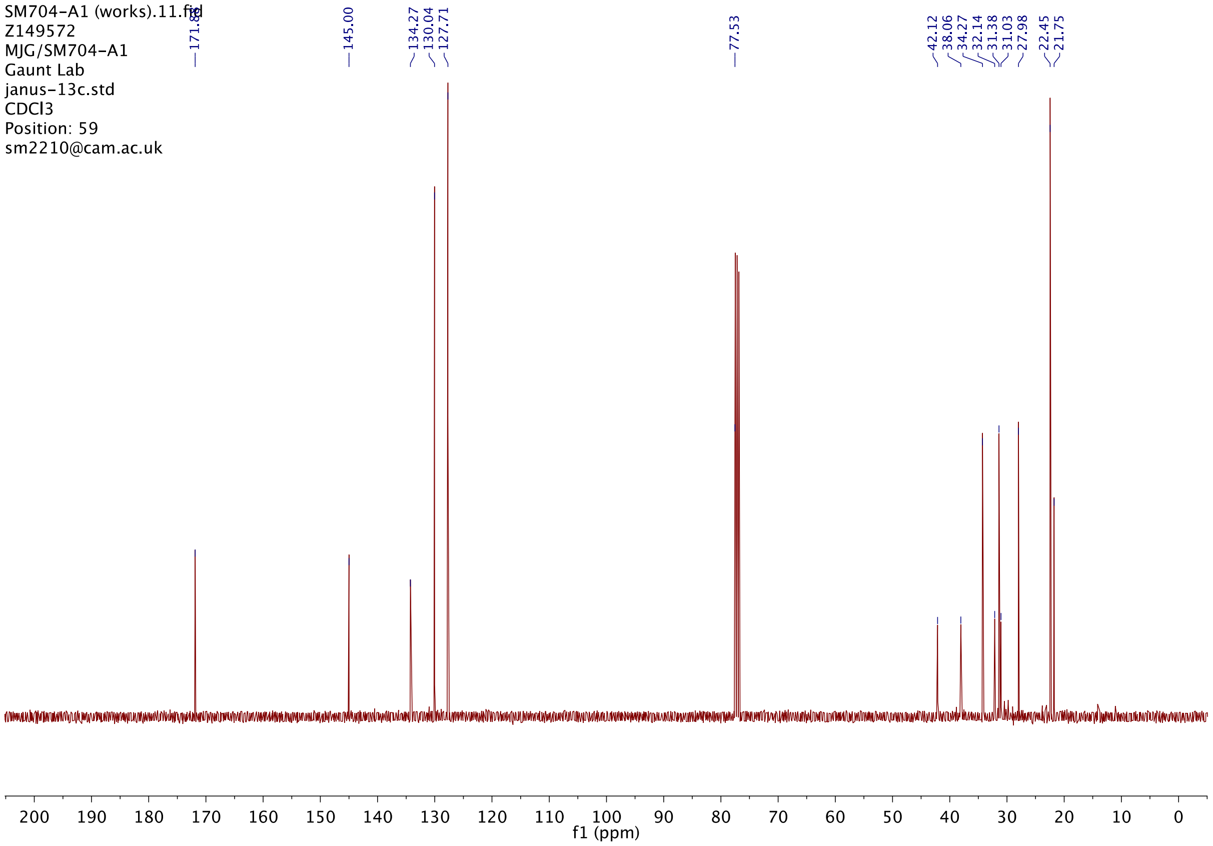
SM704-A1 (works).11.fid
Z149572
MJG/SM704-A1
Gaunt Lab
janus-13c.std
CDCl3
Position: 59
sm2210@cam.ac.uk
171.8
145.00
134.27
130.04
127.71
77.53
42.12
38.06
34.27
32.14
31.38
31.03
27.98
22.45
21.75
f1 (ppm)

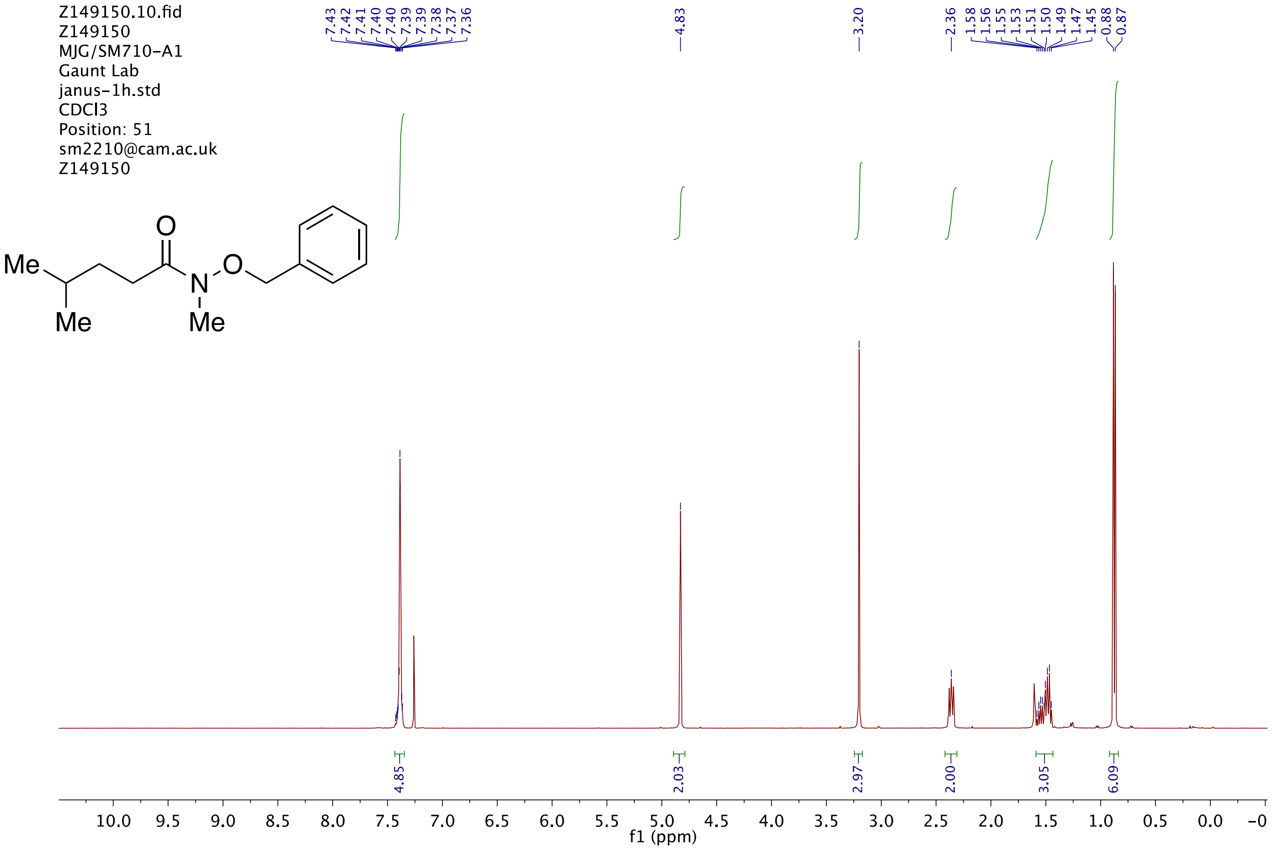
Z149150.10.fid
Z149150
MJG/SM710-A1
Gaunt Lab
janus-1h.std
CDCl3
Position: 51
sm2210@cam.ac.uk
Z149150
Me
Me
O
N
O
Me
7.43
7.42
7.41
7.40
7.39
7.39
7.38
7.37
7.36
4.83
3.20
2.36
1.58
1.56
1.55
1.53
1.51
1.50
1.49
1.47
1.45
0.88
0.87
4.85
2.03
2.97
2.00
3.05
6.09
10.0 9.5 9.0 8.5 8.0 7.5 7.0 6.5 6.0 5.5 5.0 4.5 4.0 3.5 3.0 2.5 2.0 1.5 1.0 0.5 0.0 −0
f1 (ppm)

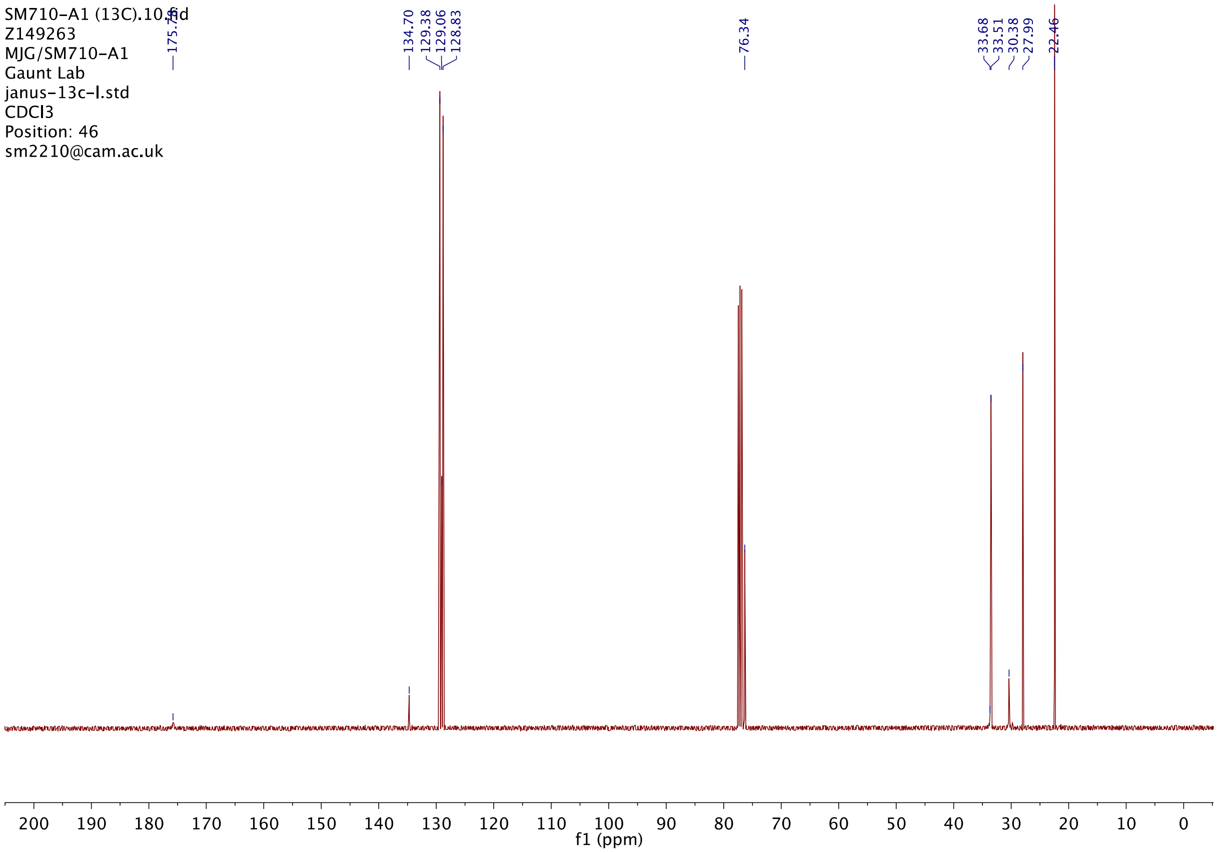
SM710-A1 (13C).10.fid
Z149263
MJG/SM710-A1
Gaunt Lab
janus-13c-l.std
CDCl3
Position: 46
sm2210@cam.ac.uk
134.70
129.38
129.06
128.83
76.34
33.68
33.51
30.38
27.99
22.46
200 190 180 170 160 150 140 130 120 110 100 90 80 70 60 50 40 30 20 10 0
f1 (ppm)

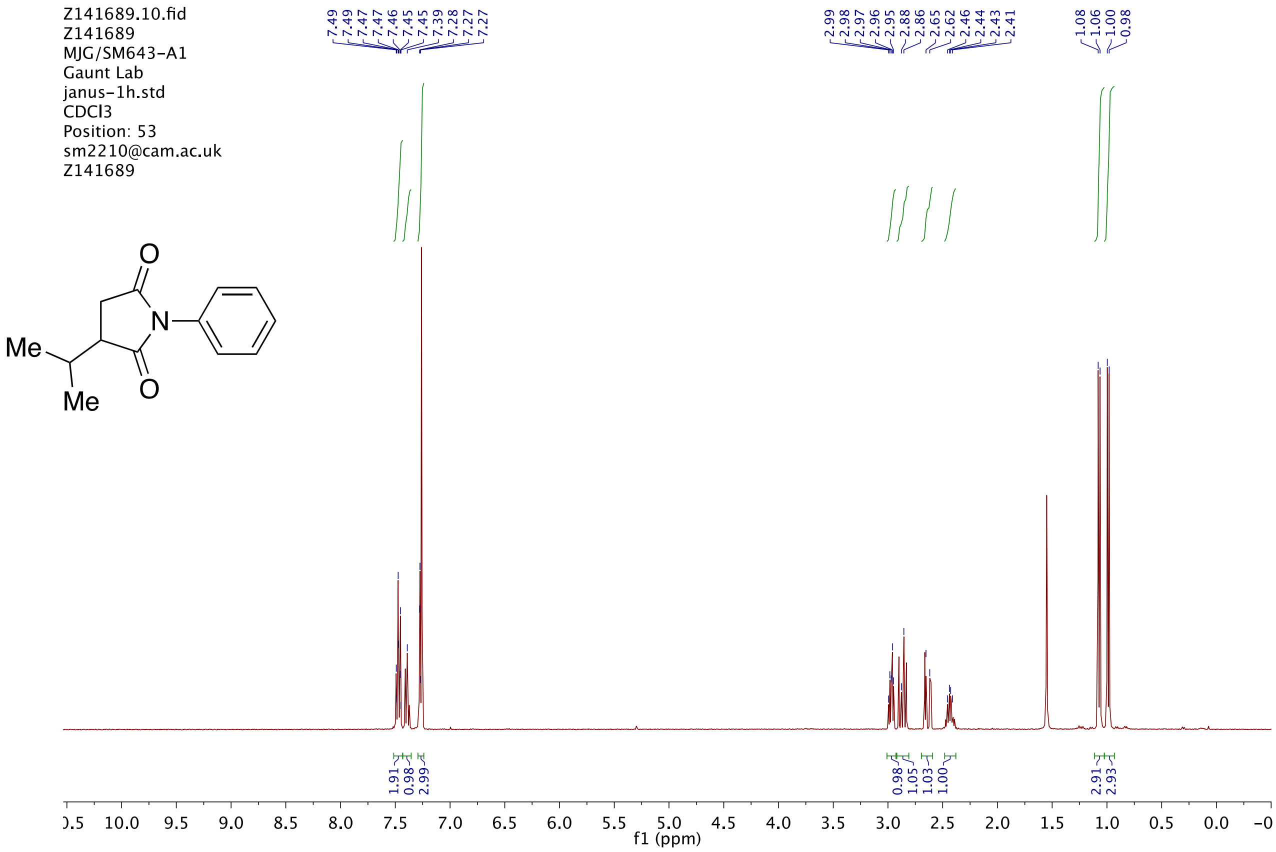
Z141689.10.fid
Z141689
MJG/SM643-A1
Gaunt Lab
janus-1h.std
CDCl3
Position: 53
sm2210@cam.ac.uk
Z141689
7.49 7.49 7.47 7.47 7.46 7.45 7.45 7.39 7.28 7.27 7.27
2.99 2.98 2.97 2.96 2.95 2.88 2.86 2.65 2.62 2.46 2.44 2.43 2.41
1.08 1.06 1.00 0.98
O
N
Me
Me
O
1.91 0.98 2.99
0.98 1.05 1.03 1.00
2.91 2.93
f1 (ppm)

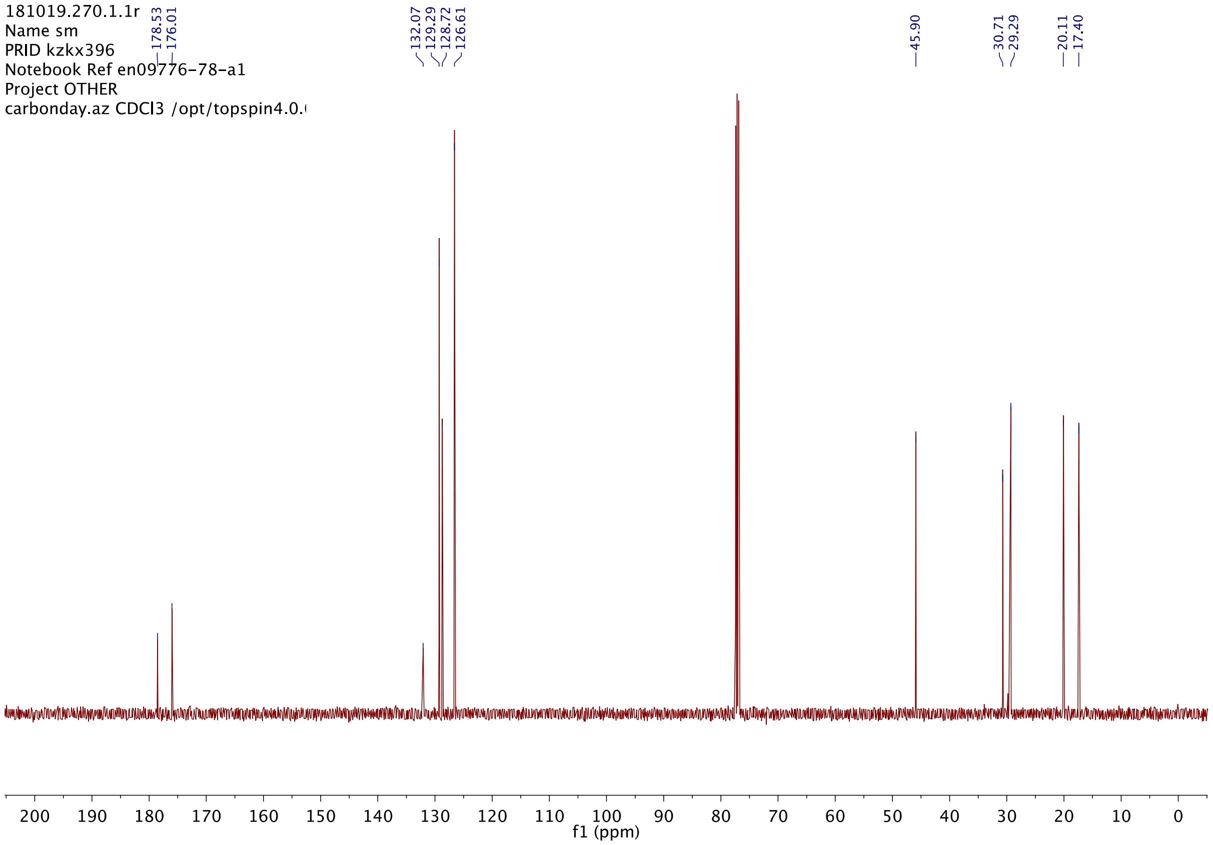
181019.270.1.1r
Name sm
PRID kzkx396
Notebook Ref en09776-78-a1
Project OTHER
178.53 176.01
132.07 129.29 128.72 126.61
45.90
30.71 29.29
20.11 17.40
f1 (ppm)

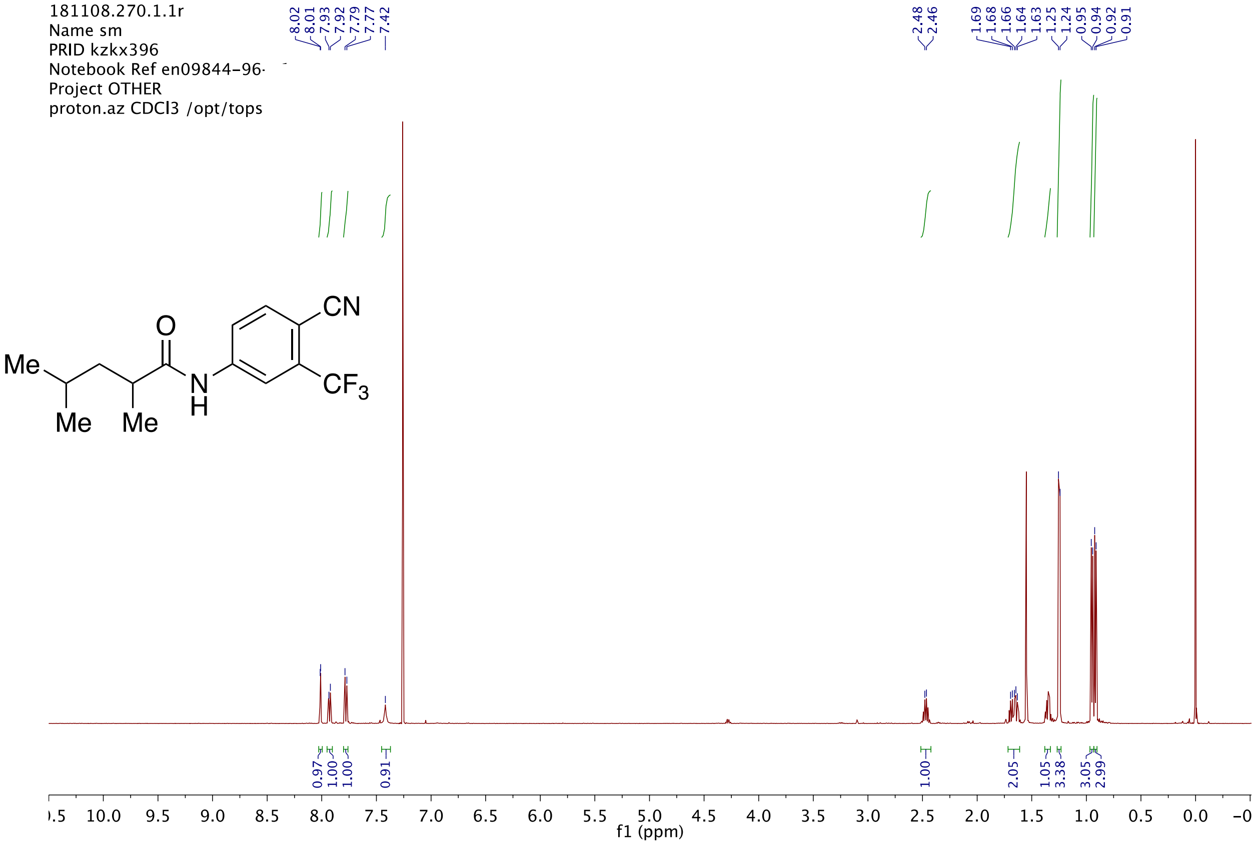
181108.270.1.1r
Name sm
PRID kzkx396
Notebook Ref en09844-96-
Project OTHER
proton.az CDCl3 /opt/tops
8.02 8.01 7.93 7.92 7.79 7.77 7.42
2.48 2.46
1.69 1.68 1.66 1.64 1.63 1.25 1.24 0.95 0.94 0.92 0.91
Me
Me
Me
O
N
H
CN
CF3
0.97 1.00 1.00 0.91 1.00 2.05 1.05 3.38 3.05 2.99
f1 (ppm)

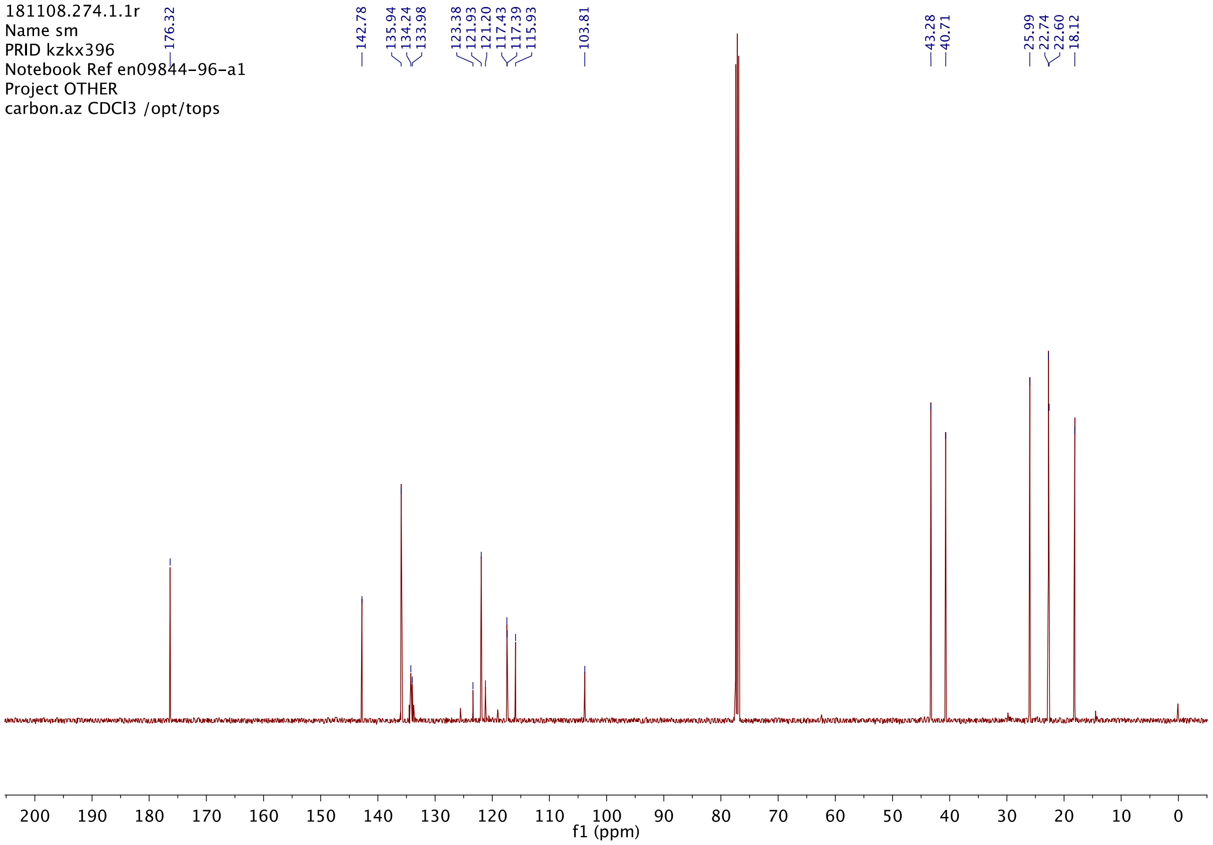
181108.274.1.1r
Name sm
PRID kzkx396
Notebook Ref en09844-96-a1
Project OTHER
carbon.az CDCl3 /opt/tops
176.32
142.78 135.94 134.24 133.98
123.38 121.93 121.20 117.43 117.39 115.93
103.81
43.28 40.71
25.99 22.74 22.60 18.12
f1 (ppm)

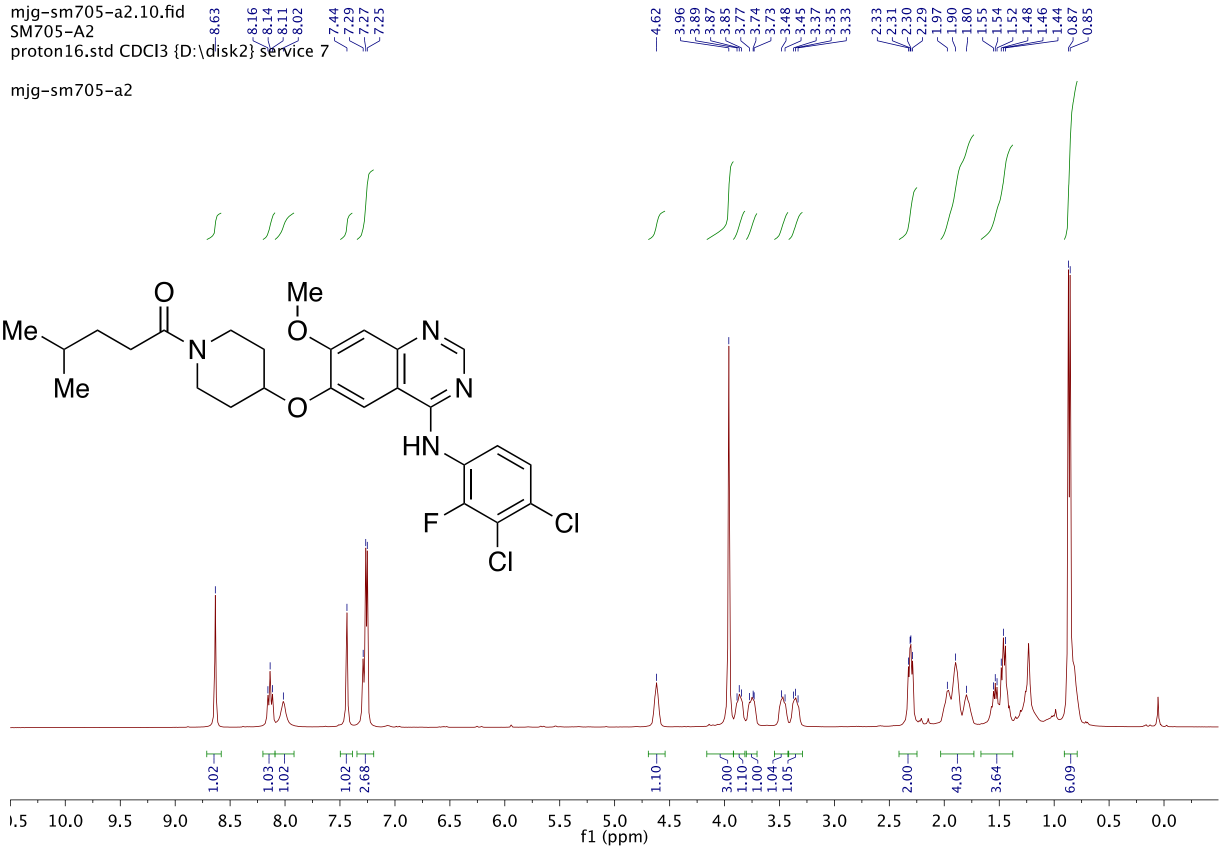
mjg-sm705-a2.10.fid
SM705-A2
proton16.std CDCl3 {D:\disk2} service 7
mjg-sm705-a2
8.63 8.16 8.14 8.11 8.02 7.44 7.29 7.27 7.25
4.62 3.96 3.89 3.87 3.85 3.77 3.74 3.73 3.48 3.45 3.37 3.35 3.33
2.33 2.31 2.30 2.29 1.97 1.90 1.80 1.55 1.54 1.52 1.48 1.46 1.44 0.87 0.85
O
Me
Me
N
O
N
N
Me
O
HN
F
Cl
Cl
1.02 1.03 1.02 1.02 2.68 1.10 3.00 1.10 1.00 1.04 1.05 2.00 4.03 3.64 6.09
f1 (ppm)

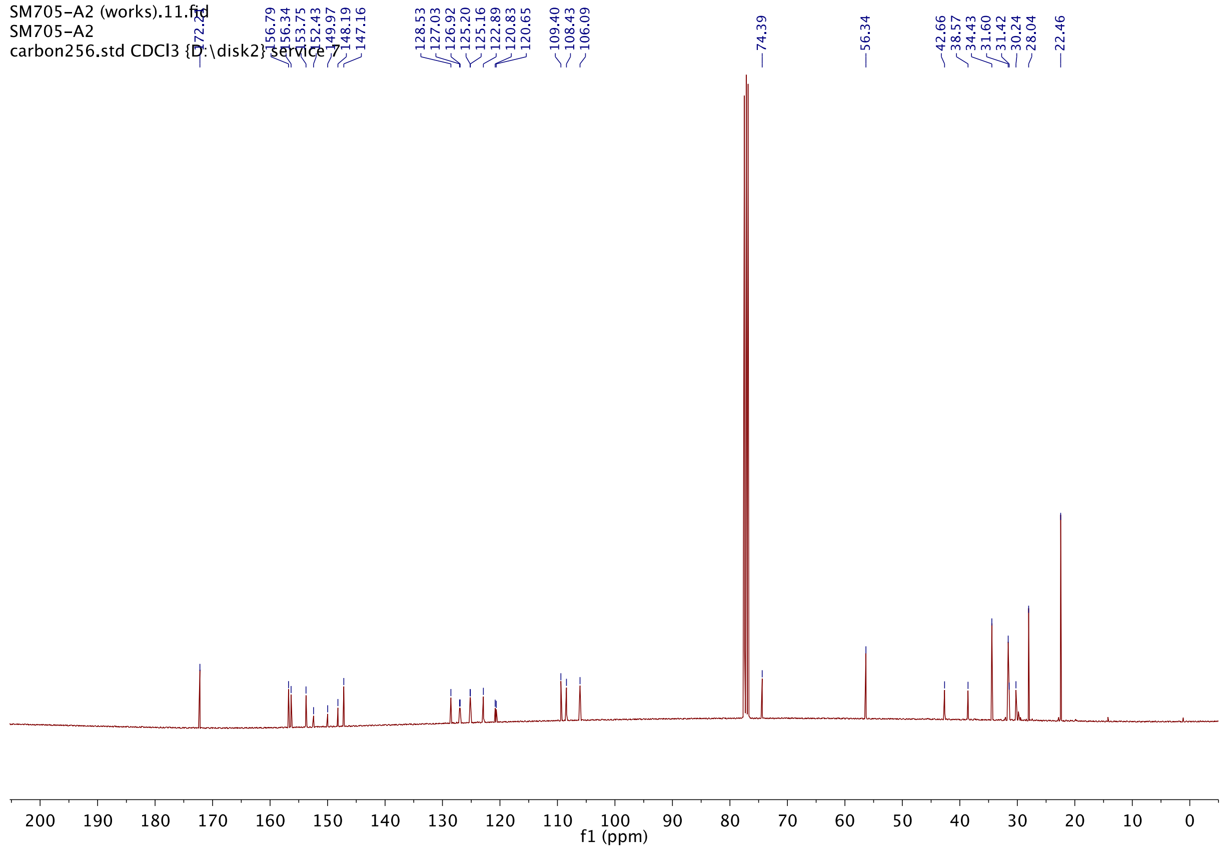
SM705-A2 (works).11.fid
SM705-A2
carbon256.std CDCl3 {D:\disk2} service 7
172.2
156.79 156.34 153.75 152.43 149.97 148.19 147.16
128.53 127.03 126.92 125.20 125.16 122.89 120.83 120.65
109.40 108.43 106.09
74.39
56.34
42.66 38.57 34.43 31.60 31.42 30.24 28.04
22.46
f1 (ppm)

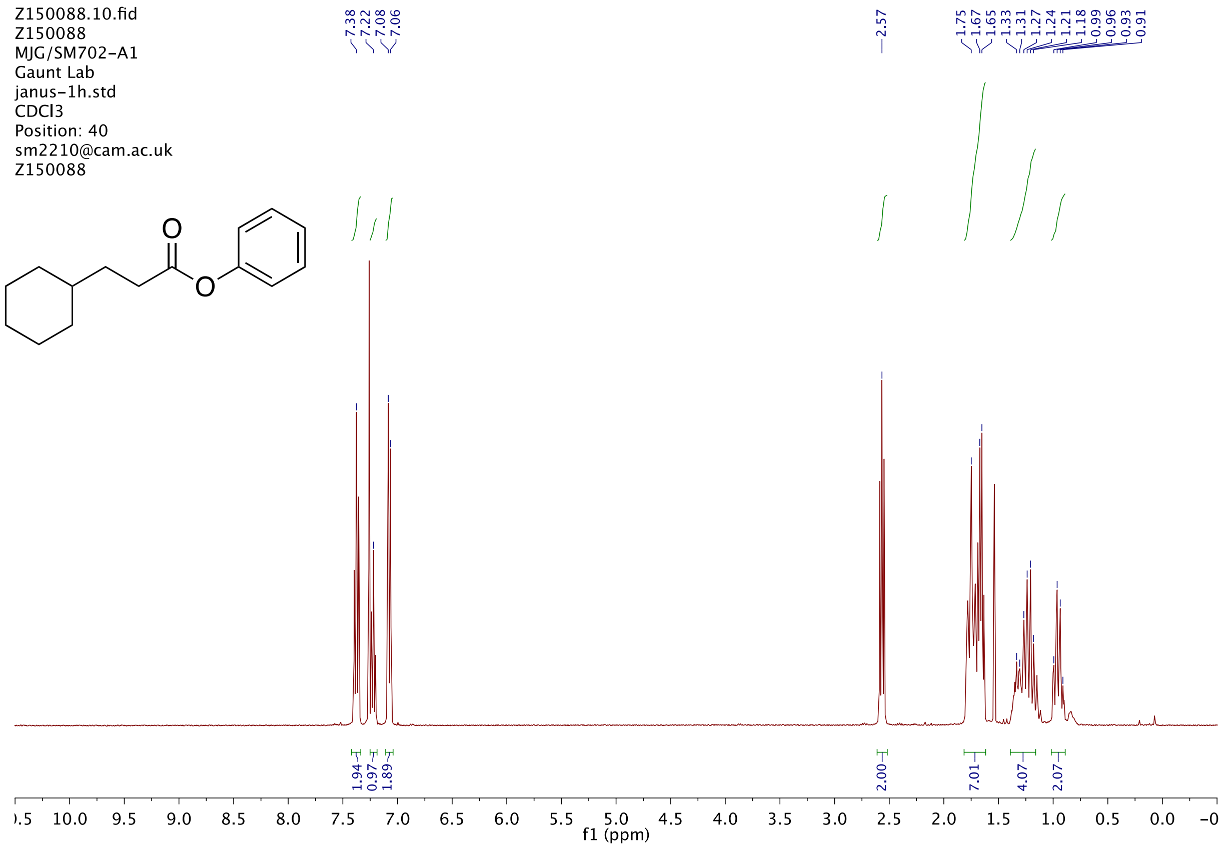
Z150088.10.fid
Z150088
MJG/SM702-A1
Gaunt Lab
janus-1h.std
CDCl3
Position: 40
sm2210@cam.ac.uk
Z150088
O
O
7.38
7.22
7.08
7.06
2.57
1.75
1.67
1.65
1.33
1.31
1.27
1.24
1.21
1.18
0.99
0.96
0.93
0.91
1.94
0.97
1.89
2.00
7.01
4.07
2.07
10.0 9.5 9.0 8.5 8.0 7.5 7.0 6.5 6.0 5.5 5.0 4.5 4.0 3.5 3.0 2.5 2.0 1.5 1.0 0.5 0.0
f1 (ppm)

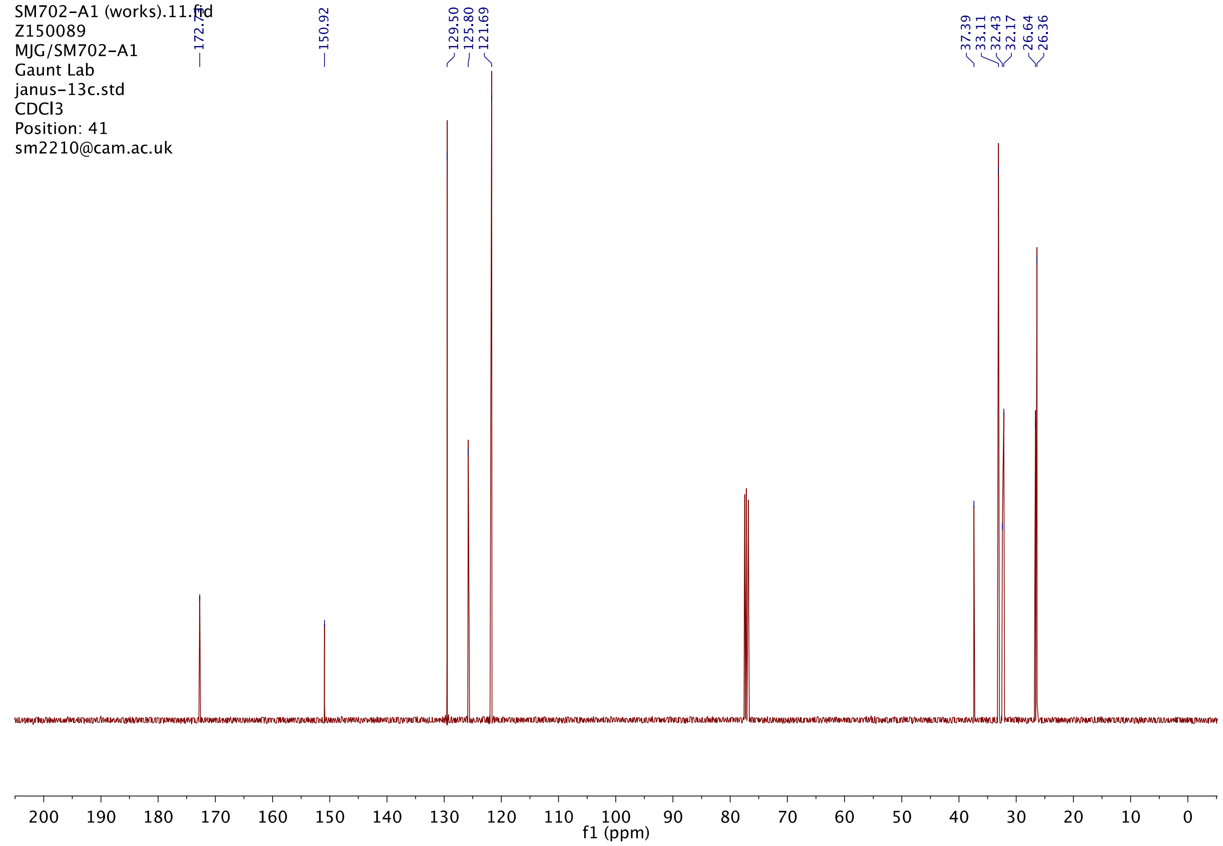
SM702-A1 (works).11.fid
Z150089
MJG/SM702-A1
Gaunt Lab
janus-13c.std
CDCl3
Position: 41
sm2210@cam.ac.uk
172.73
150.92
129.50
125.80
121.69
37.39
33.11
32.43
32.17
26.64
26.36
200 190 180 170 160 150 140 130 120 110 100 90 80 70 60 50 40 30 20 10 0
f1 (ppm)

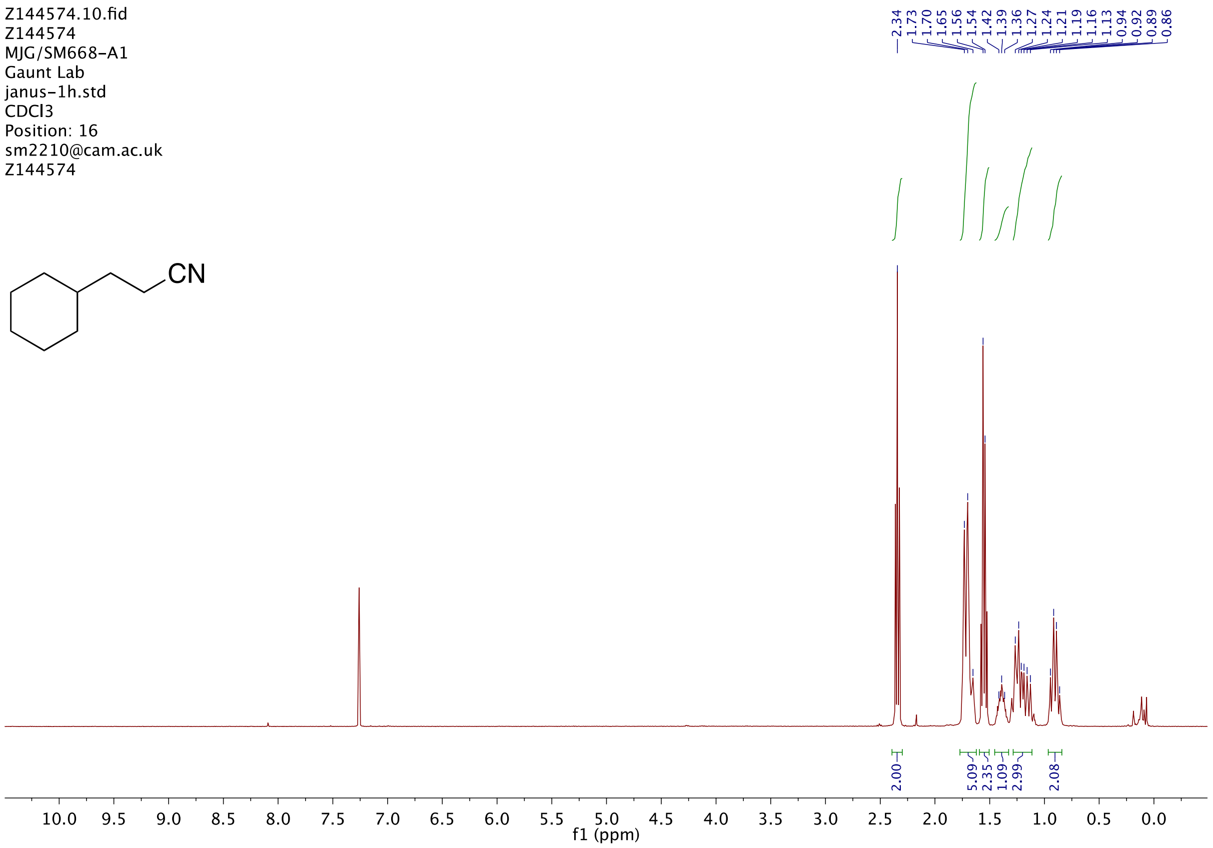
Z144574.10.fid
Z144574
MJG/SM668-A1
Gaunt Lab
janus-1h.std
CDCl3
Position: 16
sm2210@cam.ac.uk
Z144574
CN
2.34
1.73
1.70
1.65
1.56
1.54
1.42
1.39
1.36
1.27
1.24
1.21
1.19
1.16
1.13
0.94
0.92
0.89
0.86
2.00
5.09
2.35
1.09
2.99
2.08
10.0 9.5 9.0 8.5 8.0 7.5 7.0 6.5 6.0 5.5 5.0 4.5 4.0 3.5 3.0 2.5 2.0 1.5 1.0 0.5 0.0
f1 (ppm)

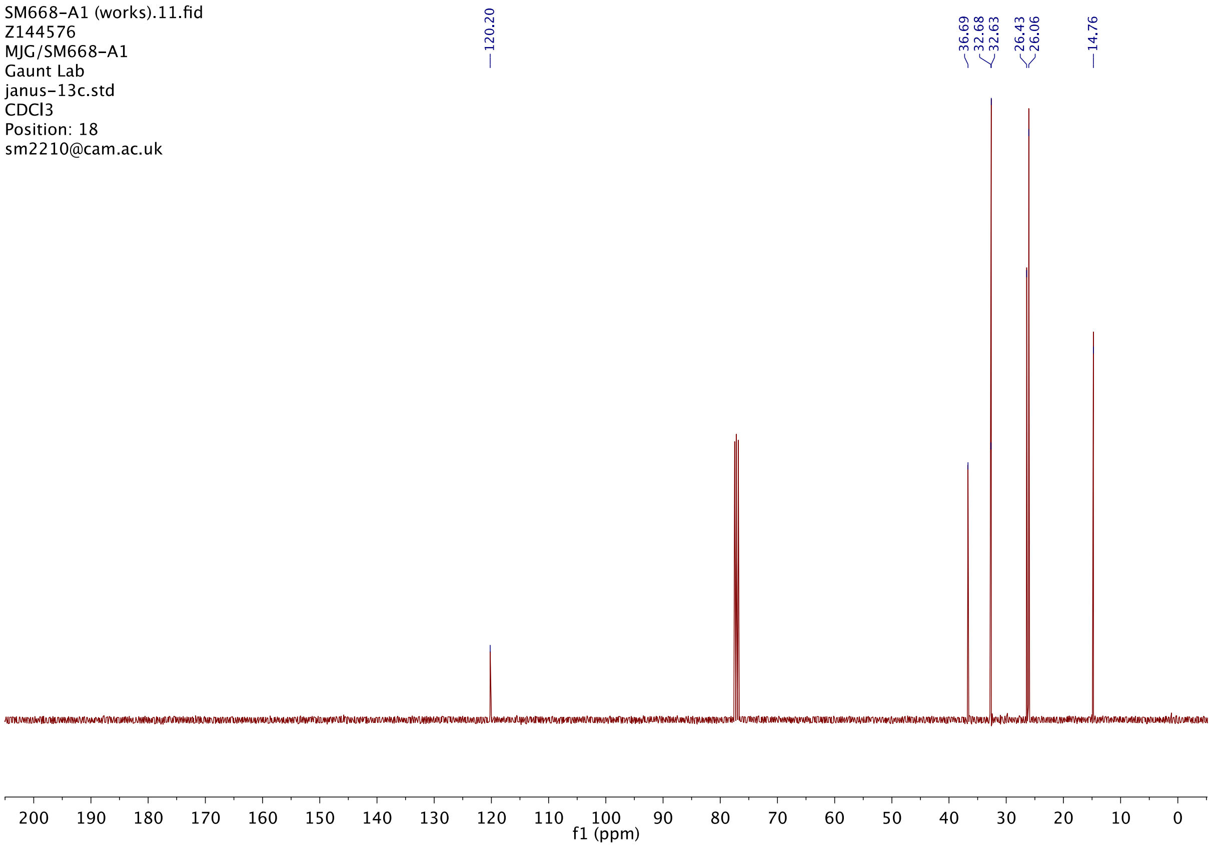
SM668-A1 (works).11.fid
Z144576
MJG/SM668-A1
Gaunt Lab
janus-13c.std
CDCl3
Position: 18
sm2210@cam.ac.uk
120.20
36.69
32.68
32.63
26.43
26.06
14.76
200 190 180 170 160 150 140 130 120 110 100 90 80 70 60 50 40 30 20 10 0
f1 (ppm)

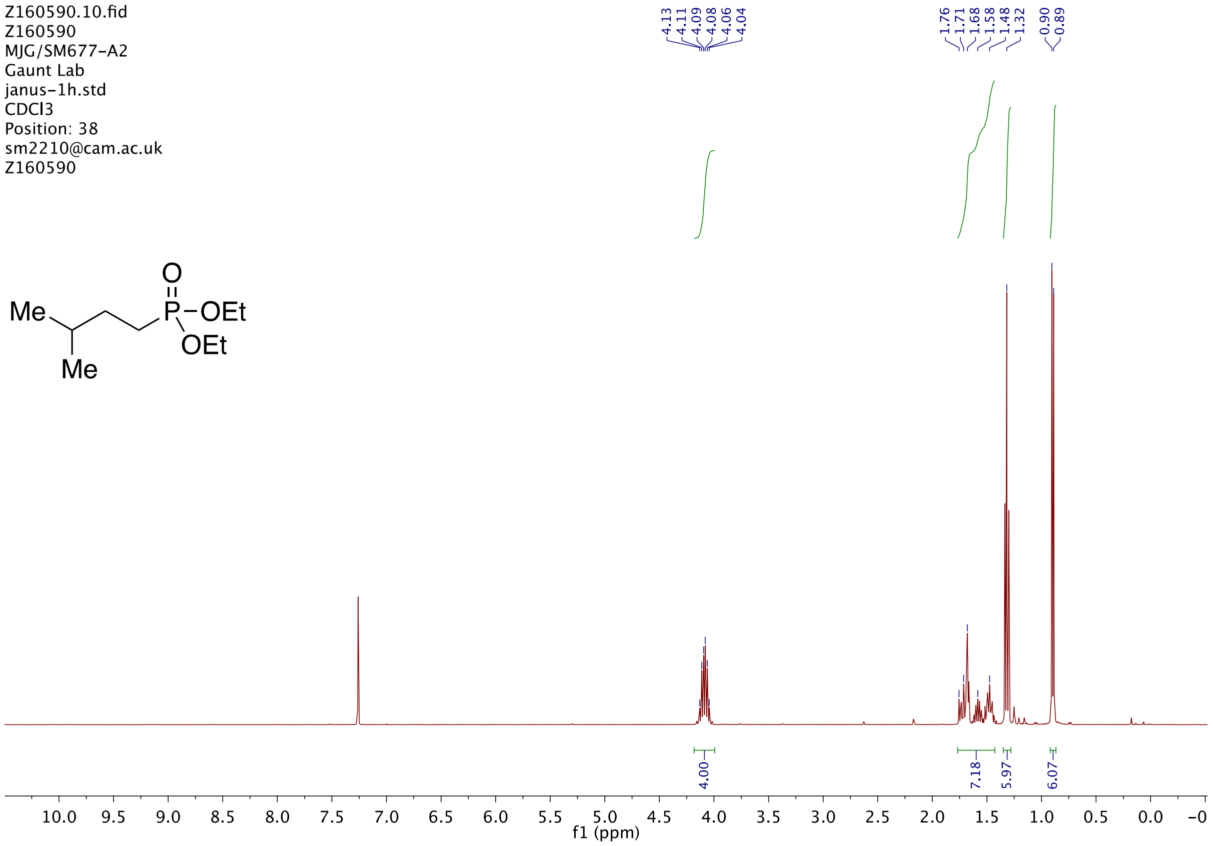
Z160590.10.fid
Z160590
MJG/SM677-A2
Gaunt Lab
janus-1h.std
CDCl3
Position: 38
sm2210@cam.ac.uk
Z160590
4.13
4.11
4.09
4.08
4.06
4.04
1.76
1.71
1.68
1.58
1.48
1.32
0.90
0.89
O
P
OEt
OEt
Me
Me
4.00
7.18
5.97
6.07
10.0
9.5
9.0
8.5
8.0
7.5
7.0
6.5
6.0
5.5
5.0
4.5
4.0
3.5
3.0
2.5
2.0
1.5
1.0
0.5
0.0
f1 (ppm)

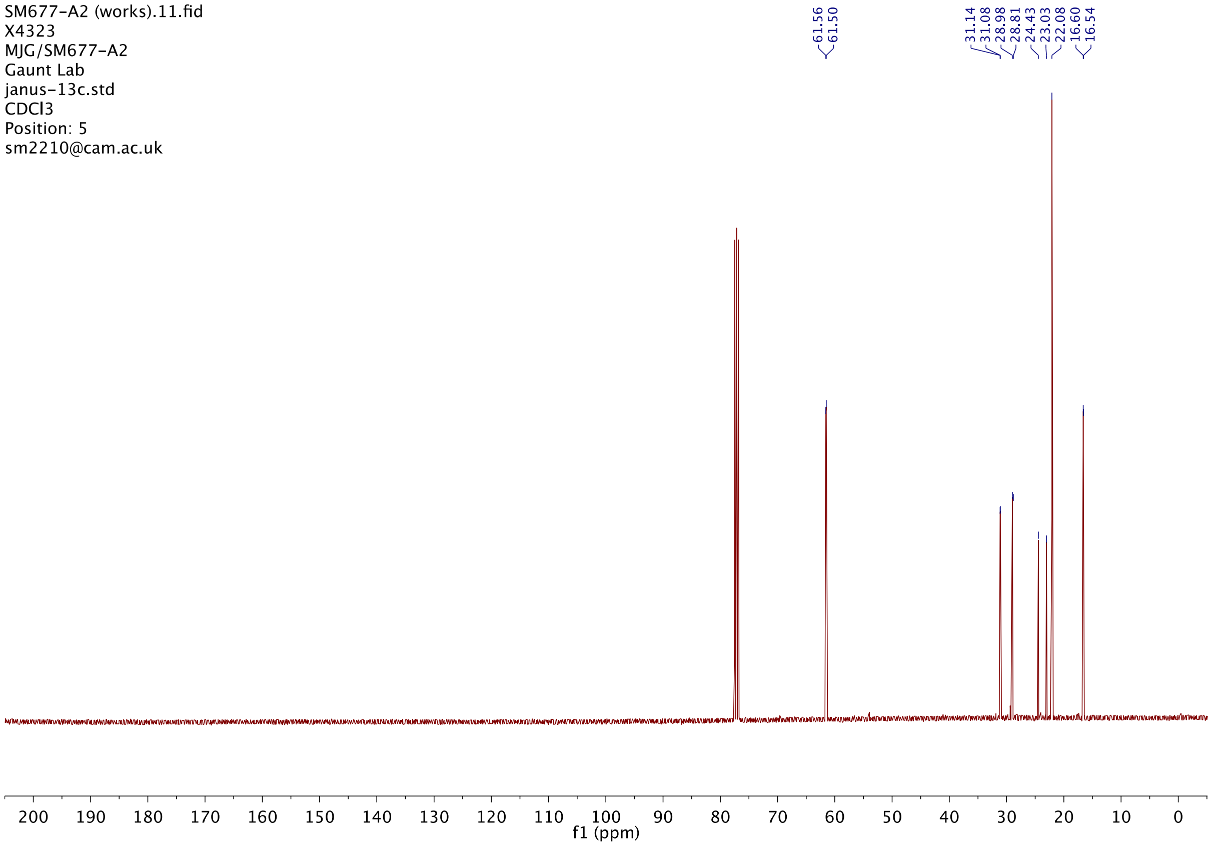
SM677-A2 (works).11.fid
X4323
MJG/SM677-A2
Gaunt Lab
janus-13c.std
CDCl3
Position: 5
sm2210@cam.ac.uk
61.56
61.50
31.14
31.08
28.98
28.81
24.43
23.03
22.08
16.60
16.54
200
190
180
170
160
150
140
130
120
110
100
90
80
70
60
50
40
30
20
10
0
f1 (ppm)

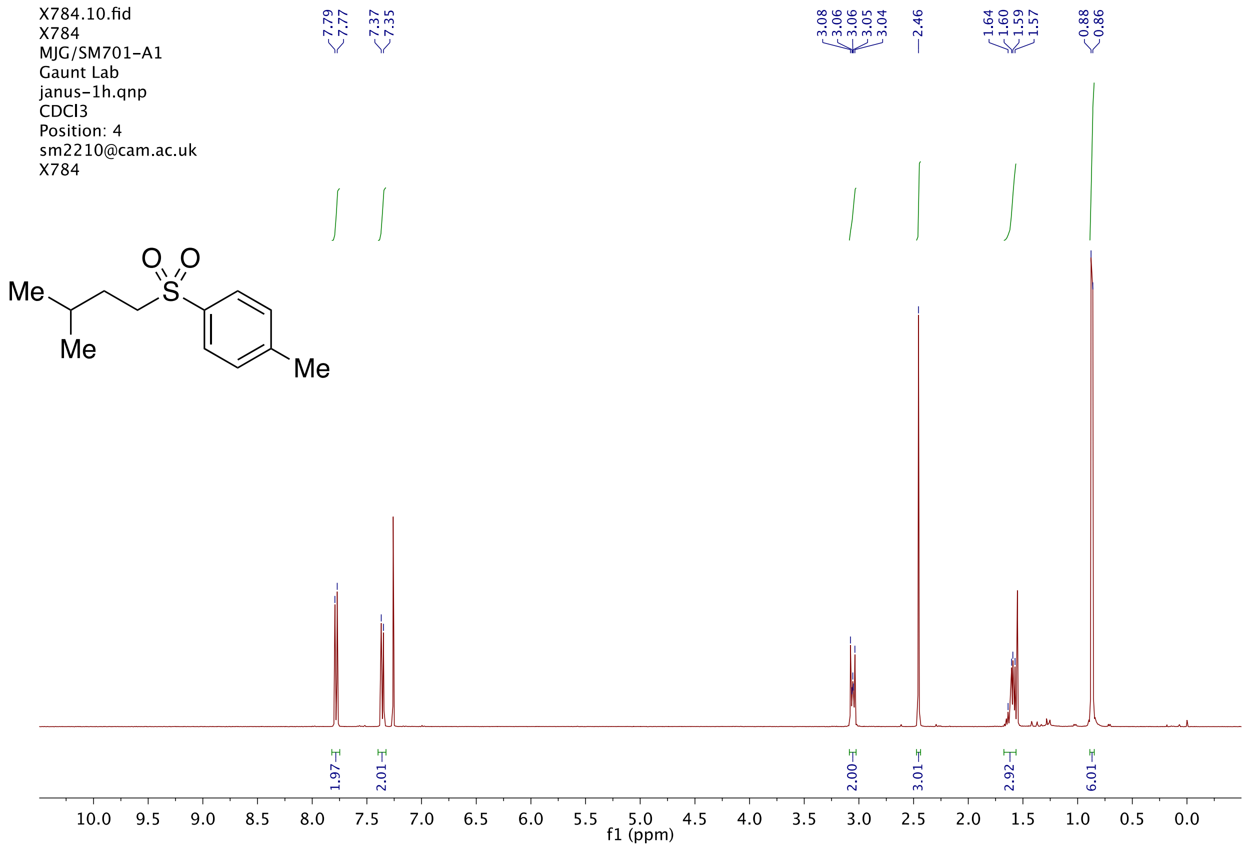
X784.10.fid
X784
MJG/SM701-A1
Gaunt Lab
janus-1h.qnp
CDCl3
Position: 4
sm2210@cam.ac.uk
X784
7.79
7.77
7.37
7.35
3.08
3.06
3.06
3.05
3.04
2.46
1.64
1.60
1.59
1.57
0.88
0.86
Me
Me
O
O
S
Me
1.97
2.01
2.00
3.01
2.92
6.01
10.0 9.5 9.0 8.5 8.0 7.5 7.0 6.5 6.0 5.5 5.0 4.5 4.0 3.5 3.0 2.5 2.0 1.5 1.0 0.5 0.0
f1 (ppm)

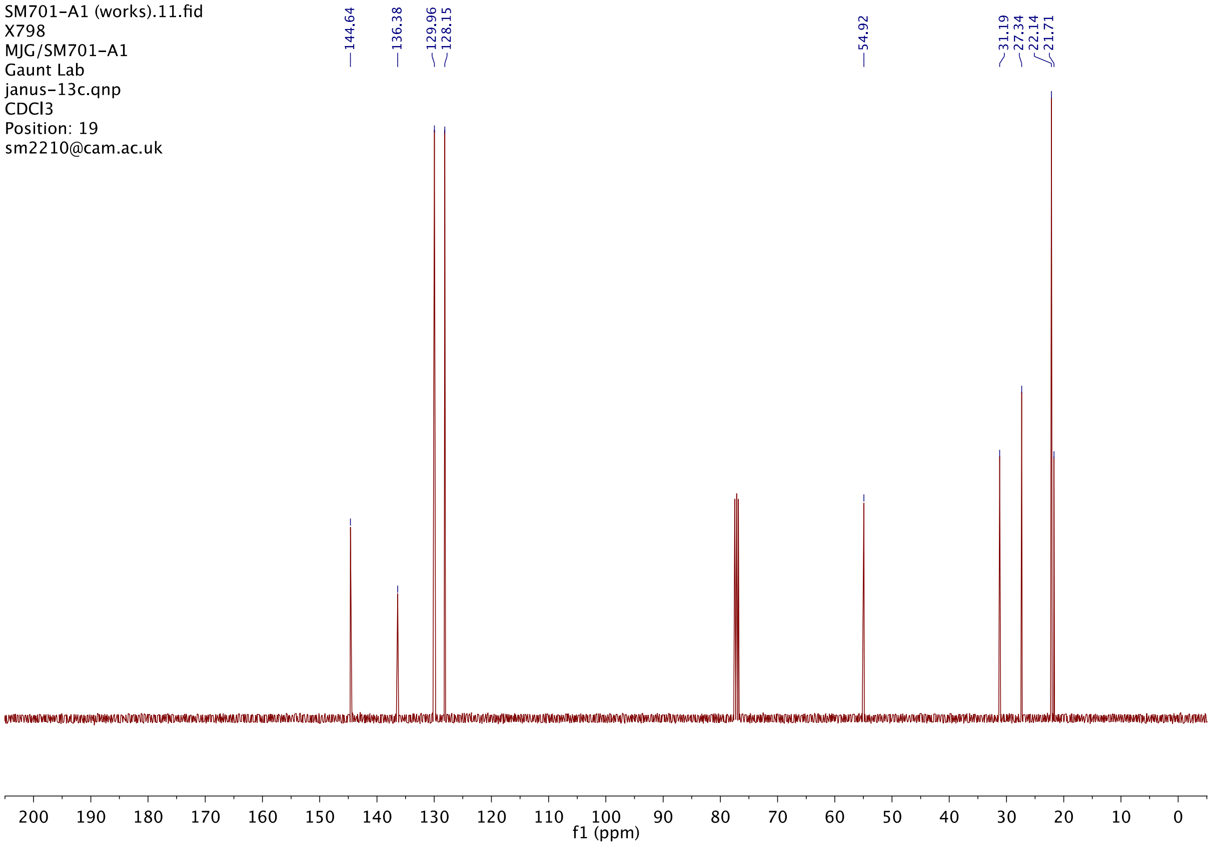
SM701-A1 (works).11.fid
X798
MJG/SM701-A1
Gaunt Lab
janus-13c.qnp
CDCl3
Position: 19
sm2210@cam.ac.uk
144.64
136.38
129.96
128.15
54.92
31.19
27.34
22.14
21.71
200 190 180 170 160 150 140 130 120 110 100 90 80 70 60 50 40 30 20 10 0
f1 (ppm)

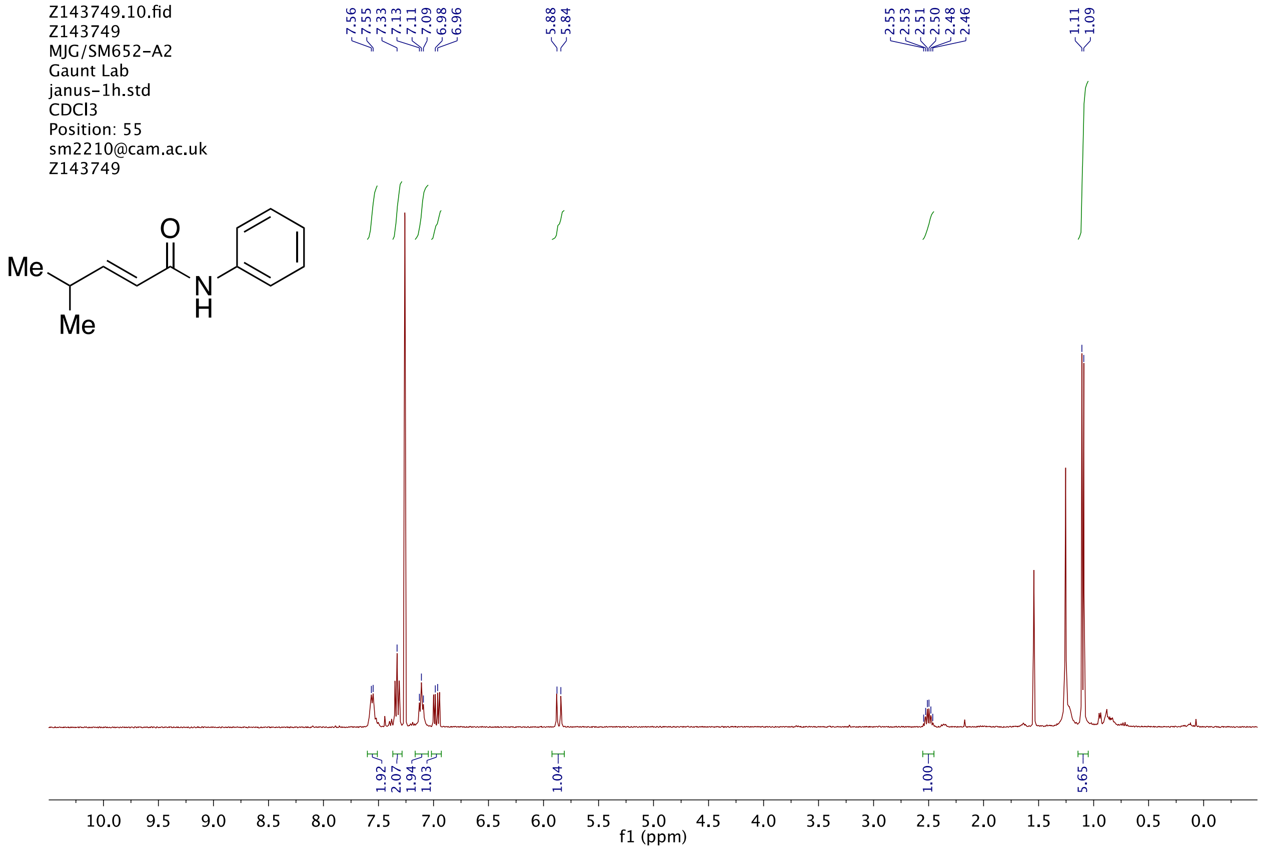
Z143749.10.fid
Z143749
MJG/SM652-A2
Gaunt Lab
janus-1h.std
CDCl3
Position: 55
sm2210@cam.ac.uk
Z143749
Me
Me
O
N
H
7.56
7.55
7.33
7.13
7.11
7.09
6.98
6.96
5.88
5.84
2.55
2.53
2.51
2.50
2.48
2.46
1.11
1.09
1.92
2.07
1.94
1.03
1.04
1.00
5.65
10.0 9.5 9.0 8.5 8.0 7.5 7.0 6.5 6.0 5.5 5.0 4.5 4.0 3.5 3.0 2.5 2.0 1.5 1.0 0.5 0.0
f1 (ppm)

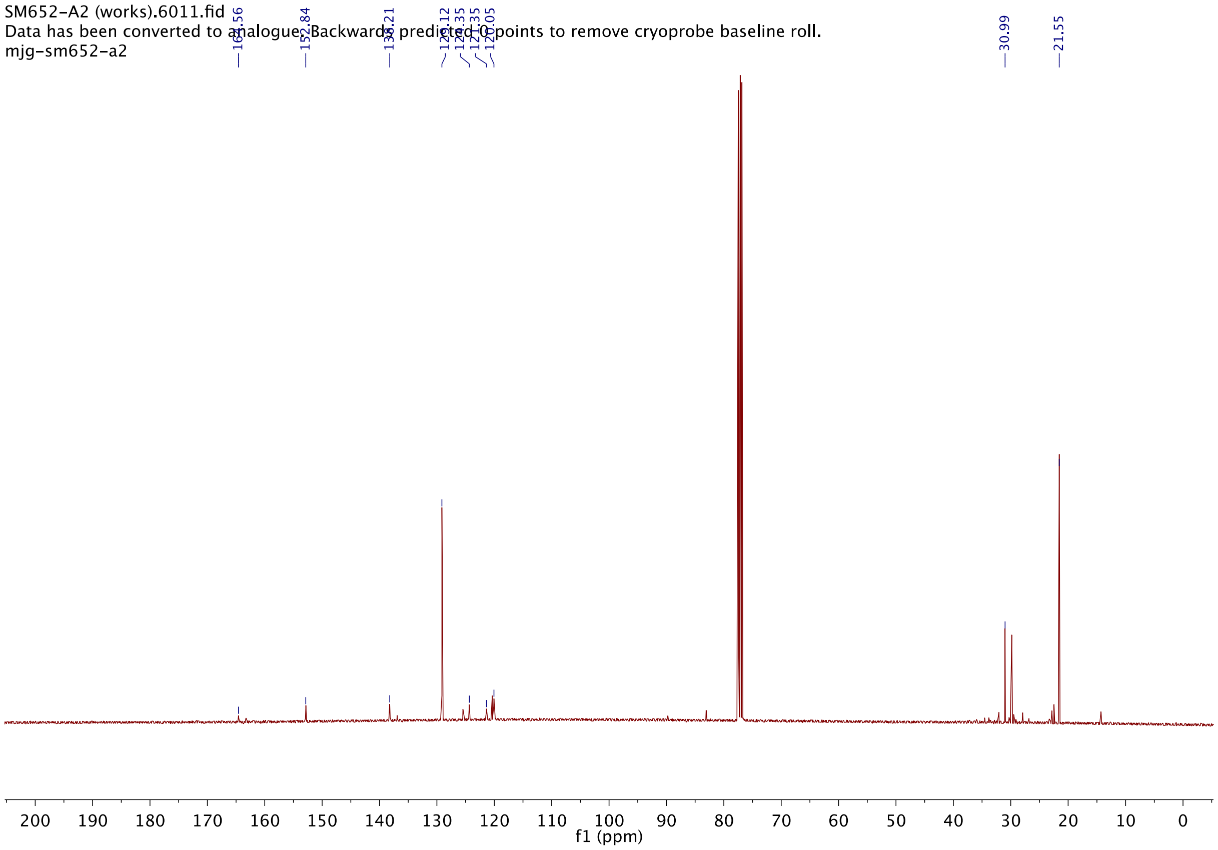
SM652-A2 (works).6011.fid
Data has been converted to analogue. Backwards predicted 0 points to remove cryoprobe baseline roll.
mjg-sm652-a2
[illegible]
152.84
138.21
129.12
124.35
121.35
30.99
21.55
200 190 180 170 160 150 140 130 120 110 100 90 80 70 60 50 40 30 20 10 0
f1 (ppm)

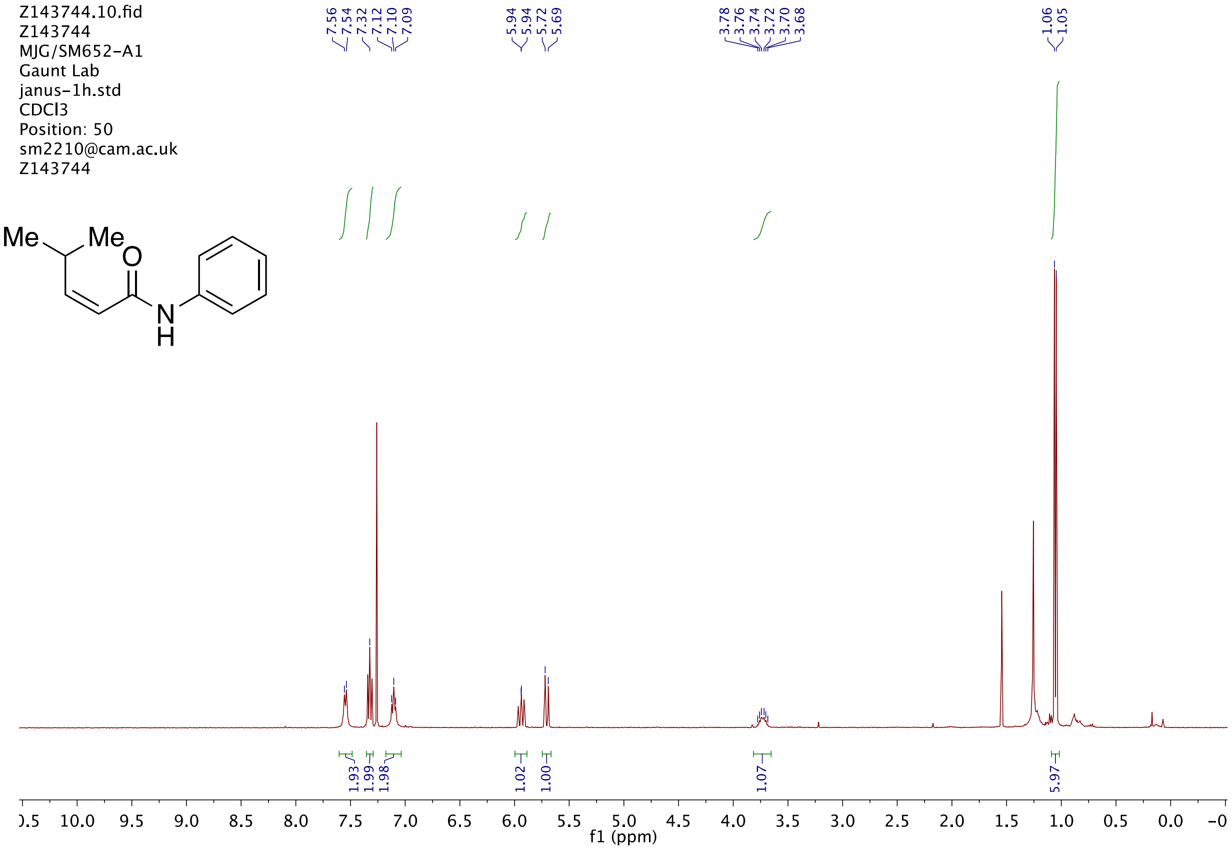
Z143744.10.fid
Z143744
MJG/SM652-A1
Gaunt Lab
janus-1h.std
CDCl3
Position: 50
sm2210@cam.ac.uk
Z143744
7.56
7.54
7.32
7.12
7.10
7.09
5.94
5.94
5.72
5.69
3.78
3.76
3.74
3.72
3.70
3.68
1.06
1.05
Me
Me
O
N
H
1.93
1.99
1.98
1.02
1.00
1.07
5.97
f1 (ppm)

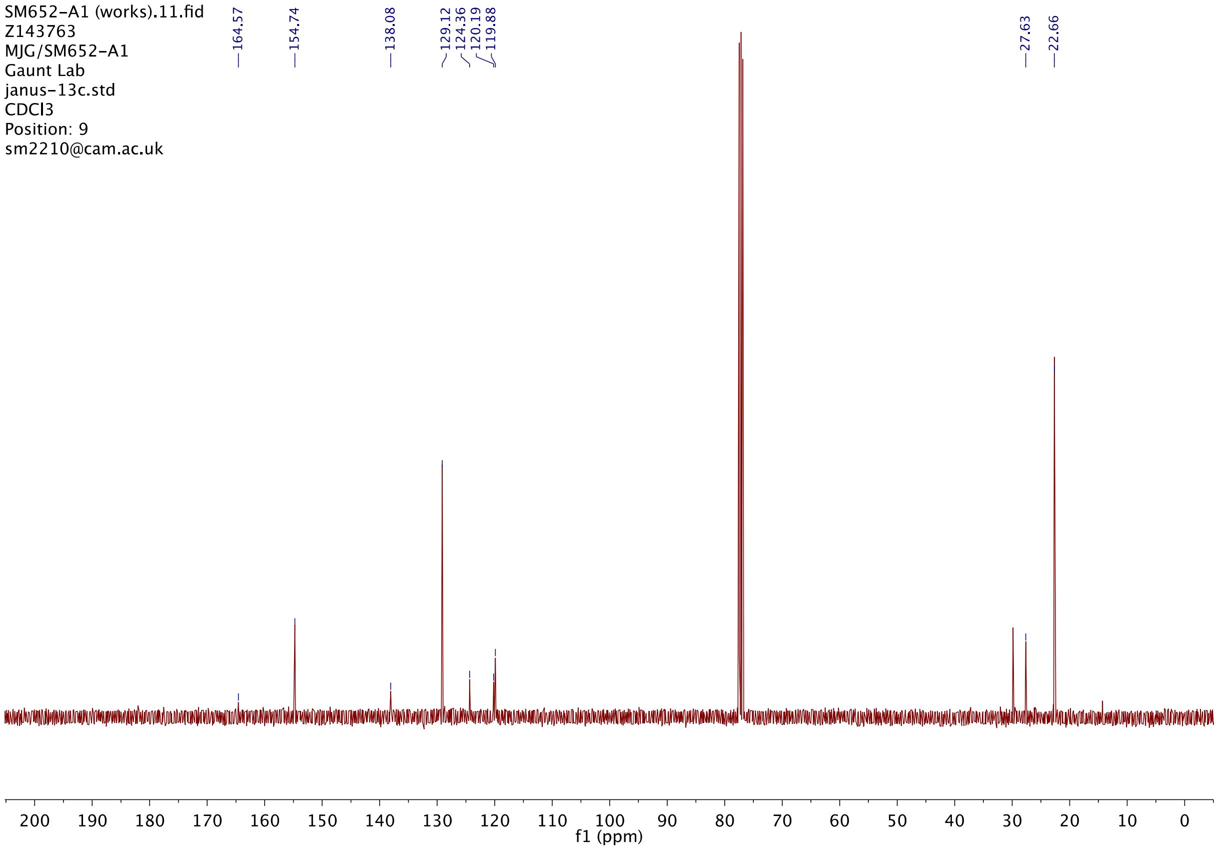
SM652-A1 (works).11.fid
Z143763
MJG/SM652-A1
Gaunt Lab
janus-13c.std
CDCl3
Position: 9
sm2210@cam.ac.uk
164.57
154.74
138.08
129.12
124.36
120.19
119.88
27.63
22.66
f1 (ppm)

V352768.10.fid
V352768
MJG/SM749-A1
`Gaunt Lab
janus-1h.qnp
CDCl3
Position: 36
sm2210@cam.ac.uk
V352768

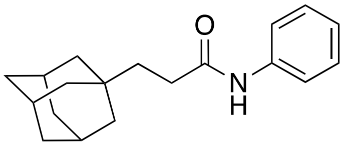

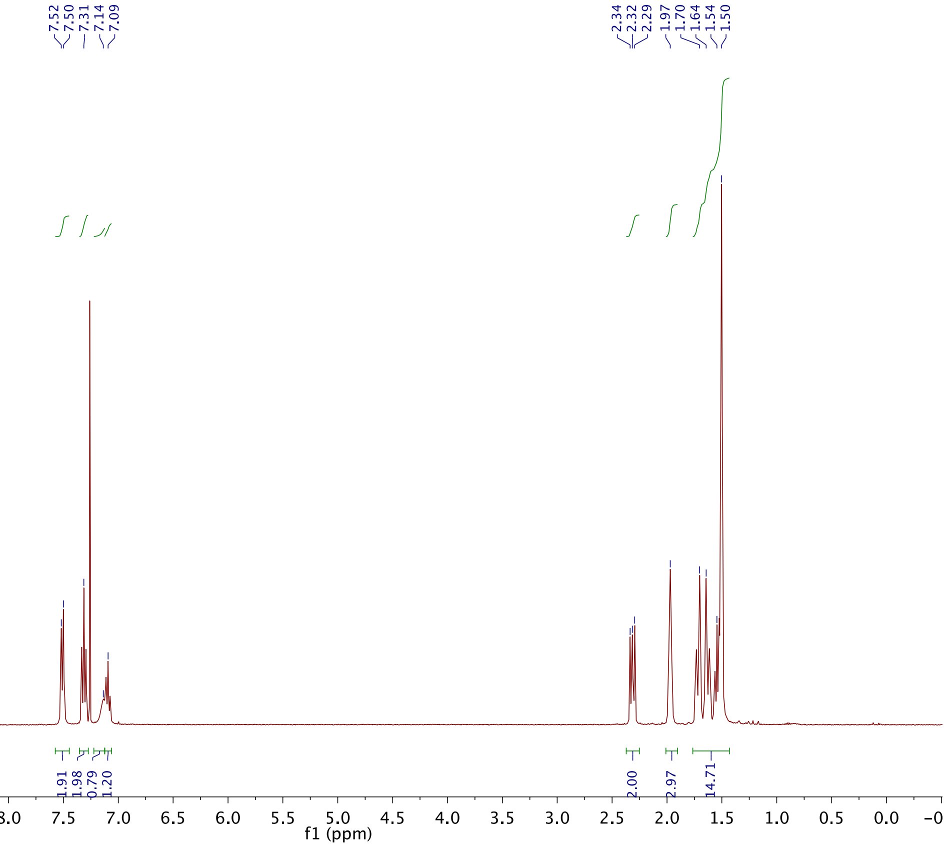

SM749-A1 (works).11.fid
X2542
MJG/SM749-A1
Gaunt Lab
janus-13c.std
CDCl3
Position: 24
sm2210@cam.ac.uk

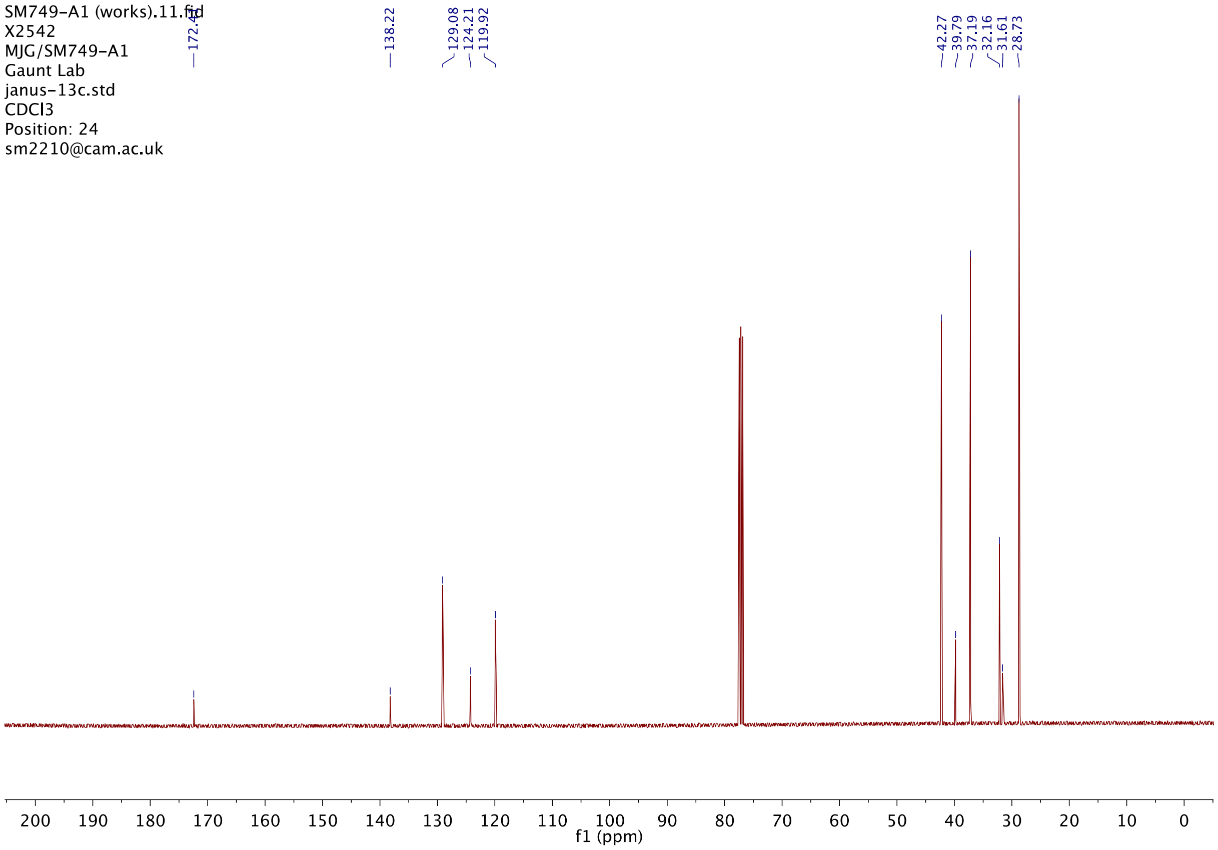

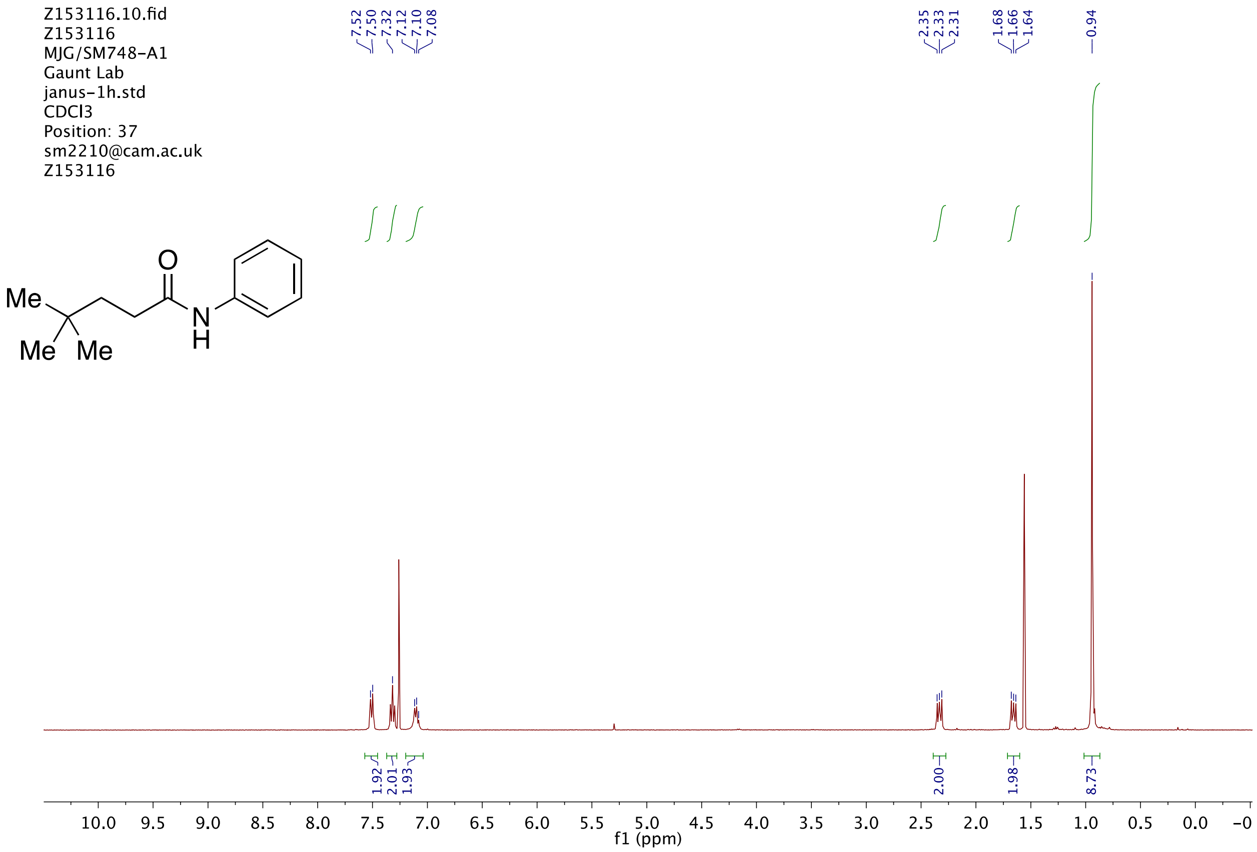
Z153116.10.fid
Z153116
MJG/SM748-A1
Gaunt Lab
janus-1h.std
CDCl3
Position: 37
sm2210@cam.ac.uk
Z153116
7.52
7.50
7.32
7.12
7.10
7.08
2.35
2.33
2.31
1.68
1.66
1.64
0.94
O
Me
N
H
Me Me
1.92
2.01
1.93
2.00
1.98
8.73
10.0 9.5 9.0 8.5 8.0 7.5 7.0 6.5 6.0 5.5 5.0 4.5 4.0 3.5 3.0 2.5 2.0 1.5 1.0 0.5 0.0 -0
f1 (ppm)

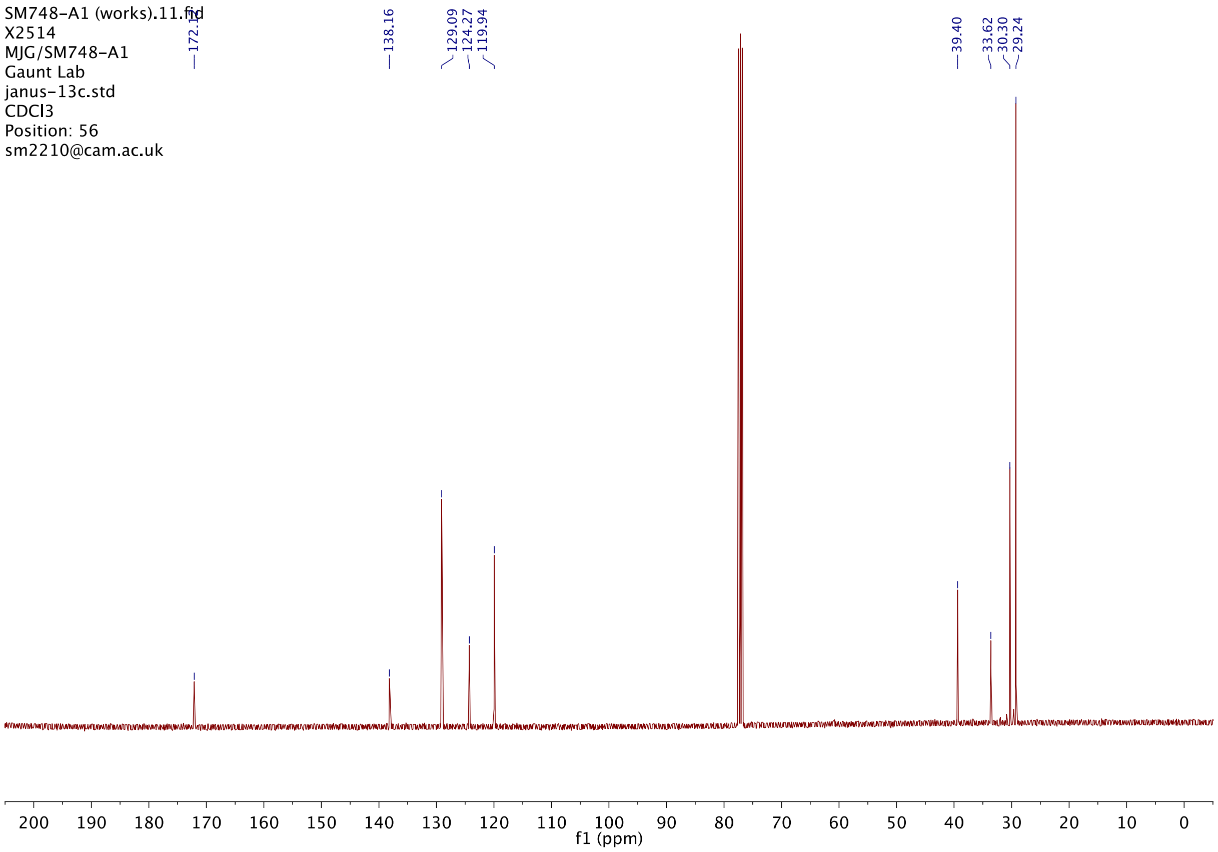
SM748-A1 (works).11.fid
X2514
MJG/SM748-A1
Gaunt Lab
janus-13c.std
CDCl3
Position: 56
sm2210@cam.ac.uk
138.16
129.09
124.27
119.94
39.40
33.62
30.30
29.24
200 190 180 170 160 150 140 130 120 110 100 90 80 70 60 50 40 30 20 10 0
f1 (ppm)

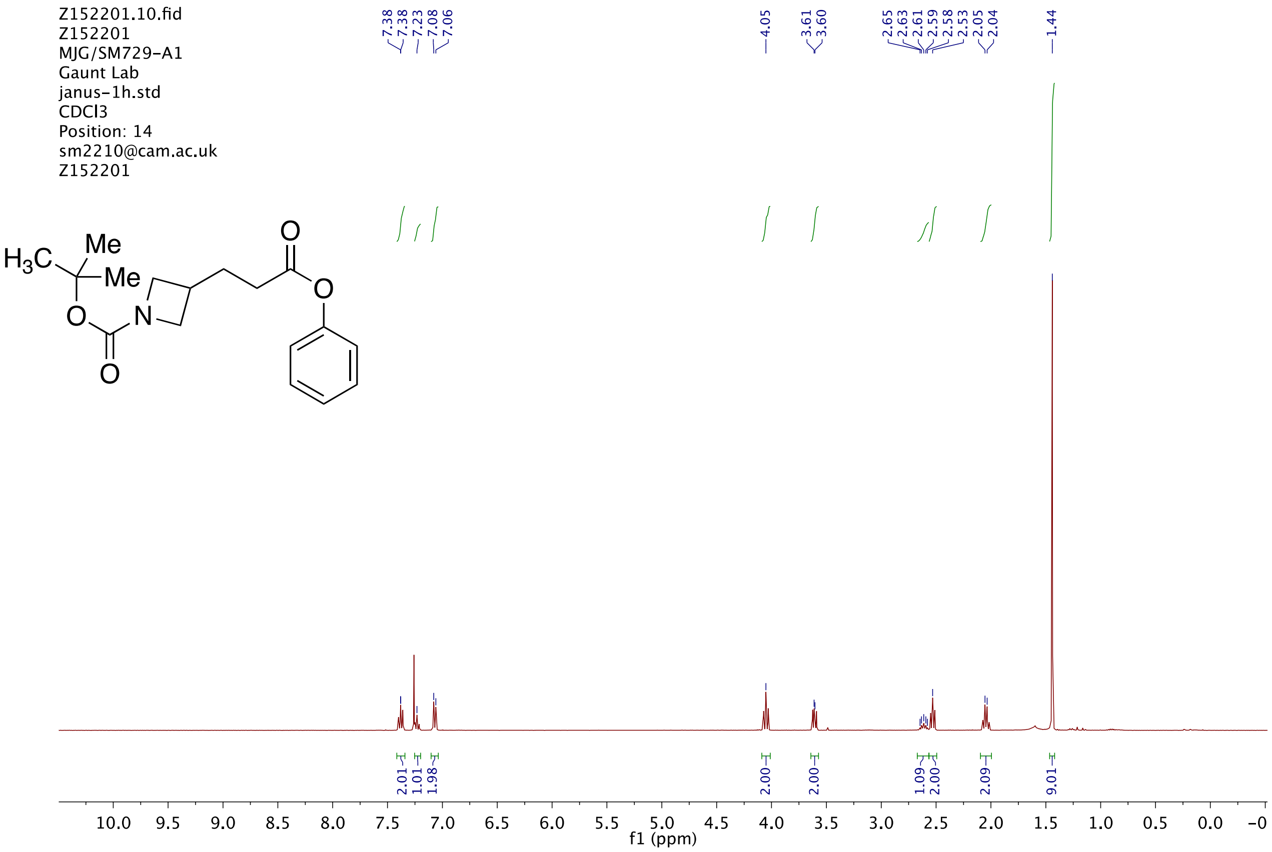
Z152201.10.fid
Z152201
MJG/SM729-A1
Gaunt Lab
janus-1h.std
CDCl3
Position: 14
sm2210@cam.ac.uk
Z152201
7.38
7.38
7.23
7.08
7.06
4.05
3.61
3.60
2.65
2.63
2.61
2.59
2.58
2.53
2.05
2.04
1.44
2.01
1.01
1.98
2.00
2.00
1.09
2.00
2.09
9.01
f1 (ppm)
H3C
Me
Me
O
N
O
O
O

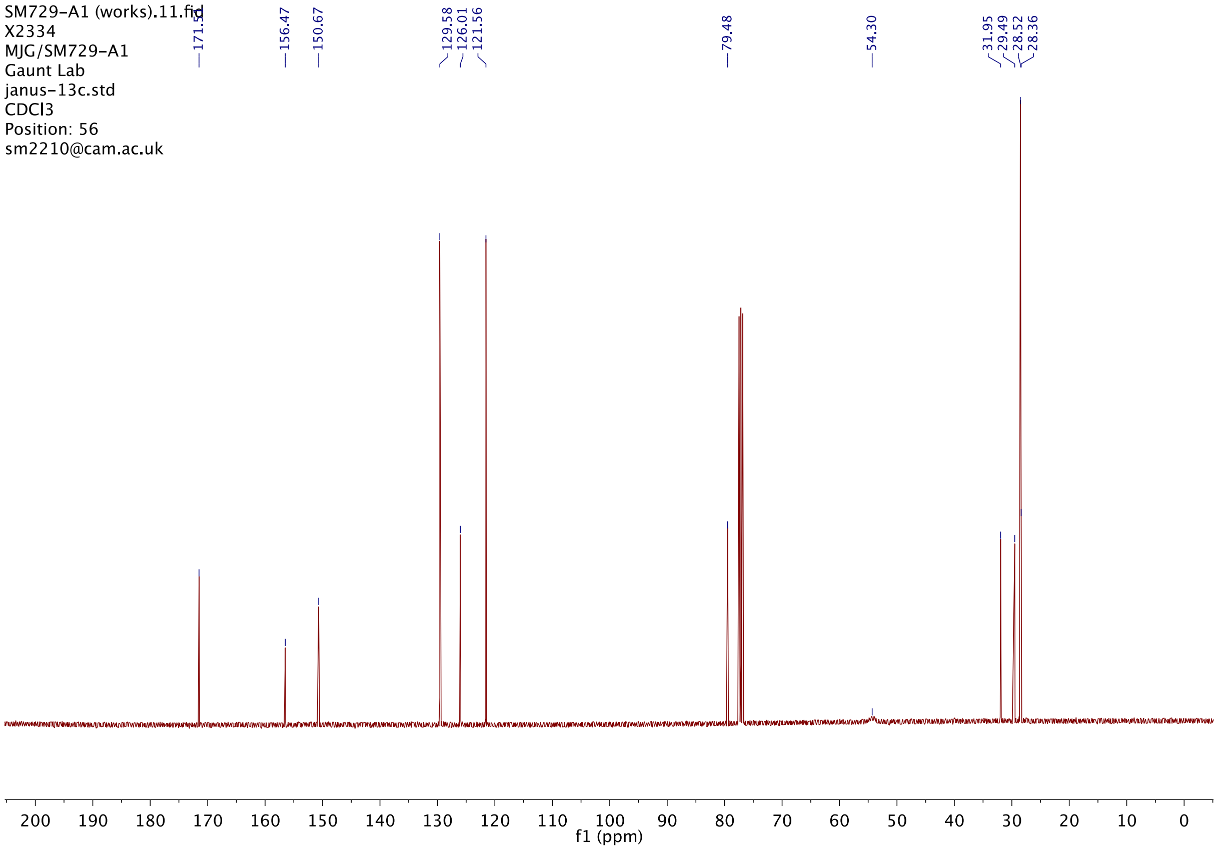
SM729-A1 (works).11.fid
X2334
MJG/SM729-A1
Gaunt Lab
janus-13c.std
CDCl3
Position: 56
sm2210@cam.ac.uk
156.47
150.67
129.58
126.01
121.56
79.48
54.30
31.95
29.49
28.52
28.36
f1 (ppm)

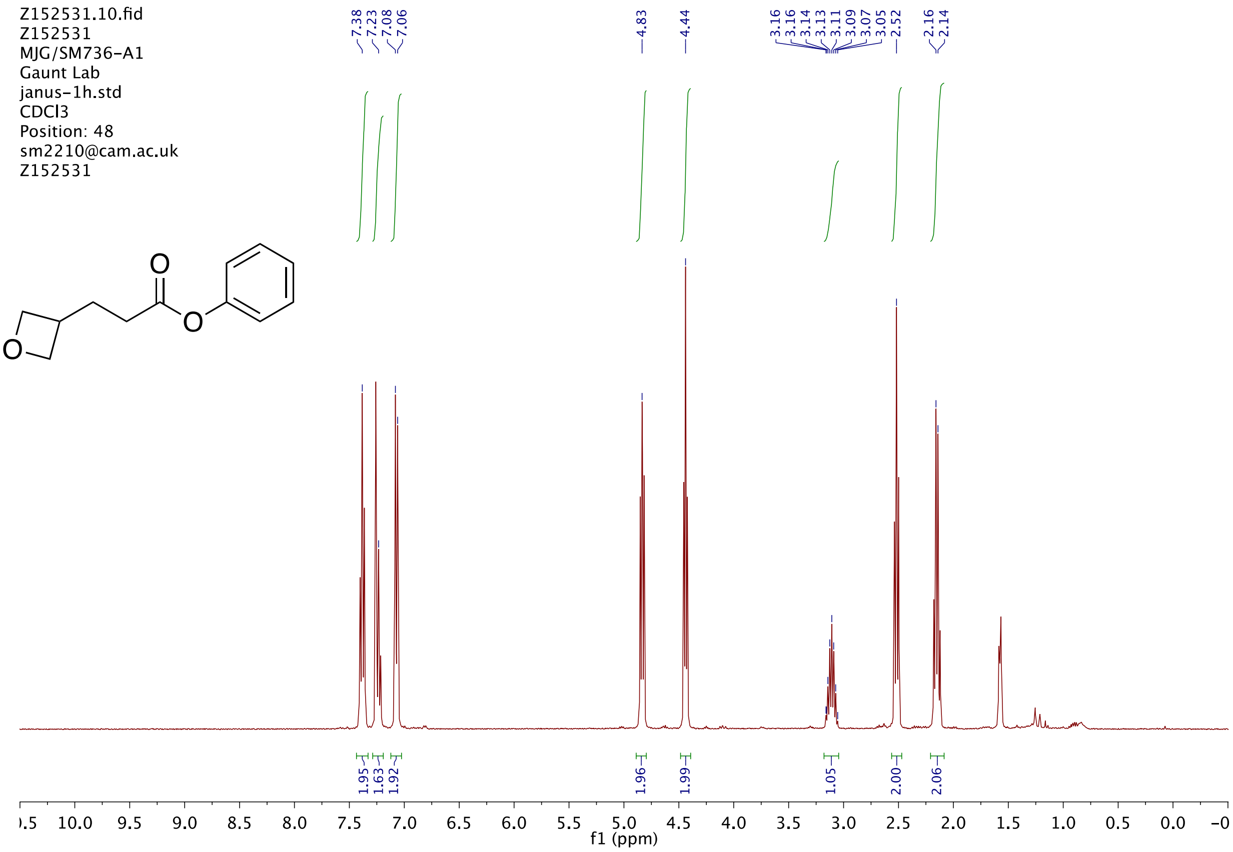
Z152531.10.fid
Z152531
MJG/SM736-A1
Gaunt Lab
janus-1h.std
CDCl3
Position: 48
sm2210@cam.ac.uk
Z152531
7.38
7.23
7.08
7.06
4.83
4.44
3.16
3.16
3.14
3.13
3.11
3.09
3.07
3.05
2.52
2.16
2.14
1.95
1.63
1.92
1.96
1.99
1.05
2.00
2.06
f1 (ppm)

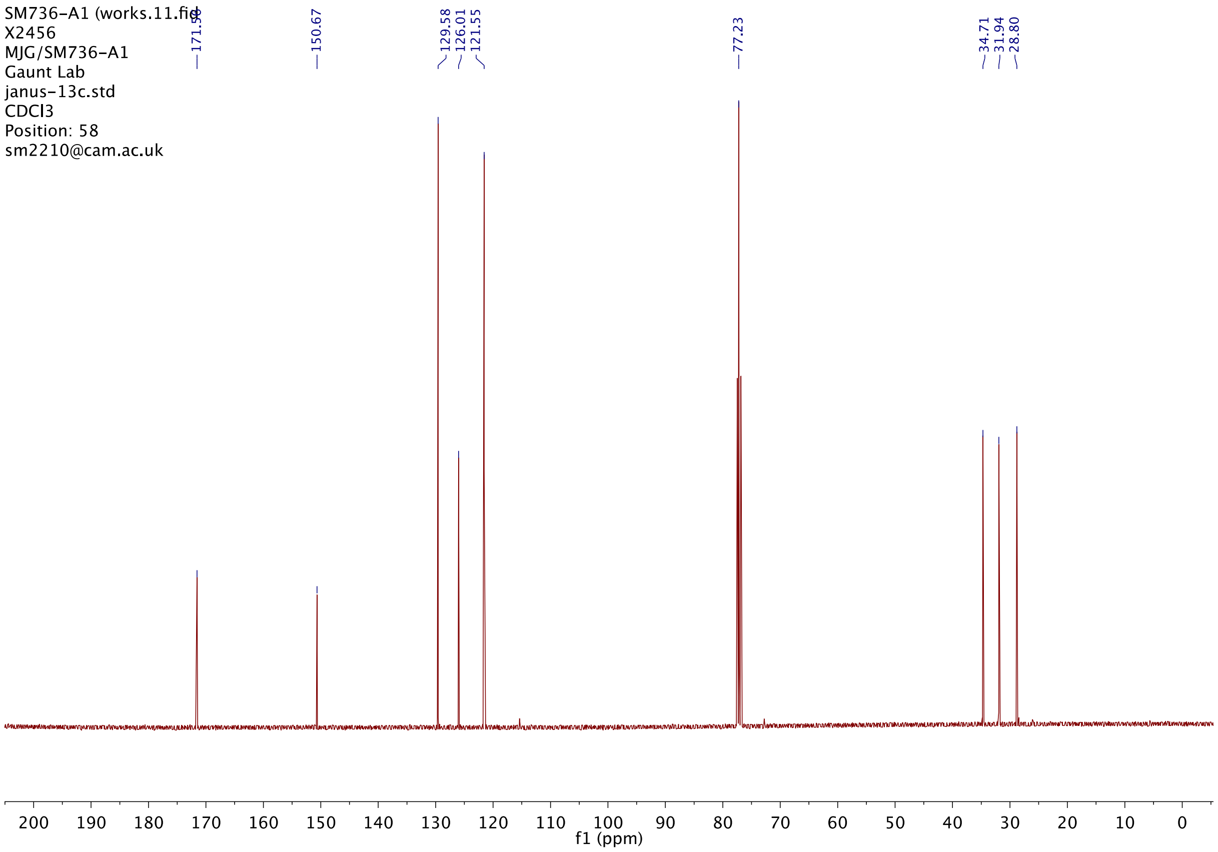
SM736-A1 (works.11.fid
X2456
MJG/SM736-A1
Gaunt Lab
janus-13c.std
CDCl3
Position: 58
sm2210@cam.ac.uk
150.67
129.58
126.01
121.55
77.23
34.71
31.94
28.80
f1 (ppm)

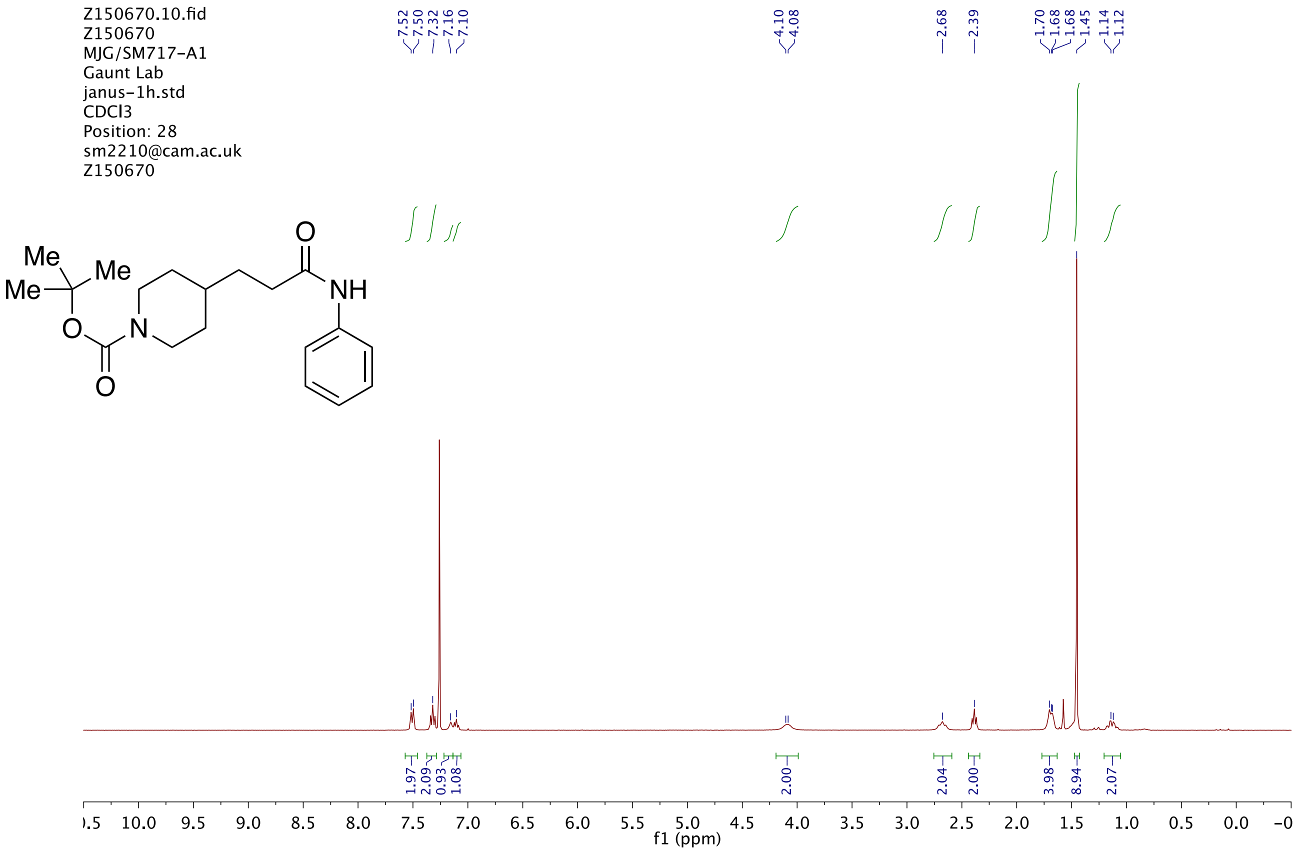
Z150670.10.fid
Z150670
MJG/SM717-A1
Gaunt Lab
janus-1h.std
CDCl3
Position: 28
sm2210@cam.ac.uk
Z150670
7.52
7.50
7.32
7.16
7.10
4.10
4.08
2.68
2.39
1.70
1.68
1.68
1.45
1.14
1.12
Me
Me
Me
O
N
O
O
NH
1.97
2.09
0.93
1.08
2.00
2.04
2.00
3.98
8.94
2.07
f1 (ppm)

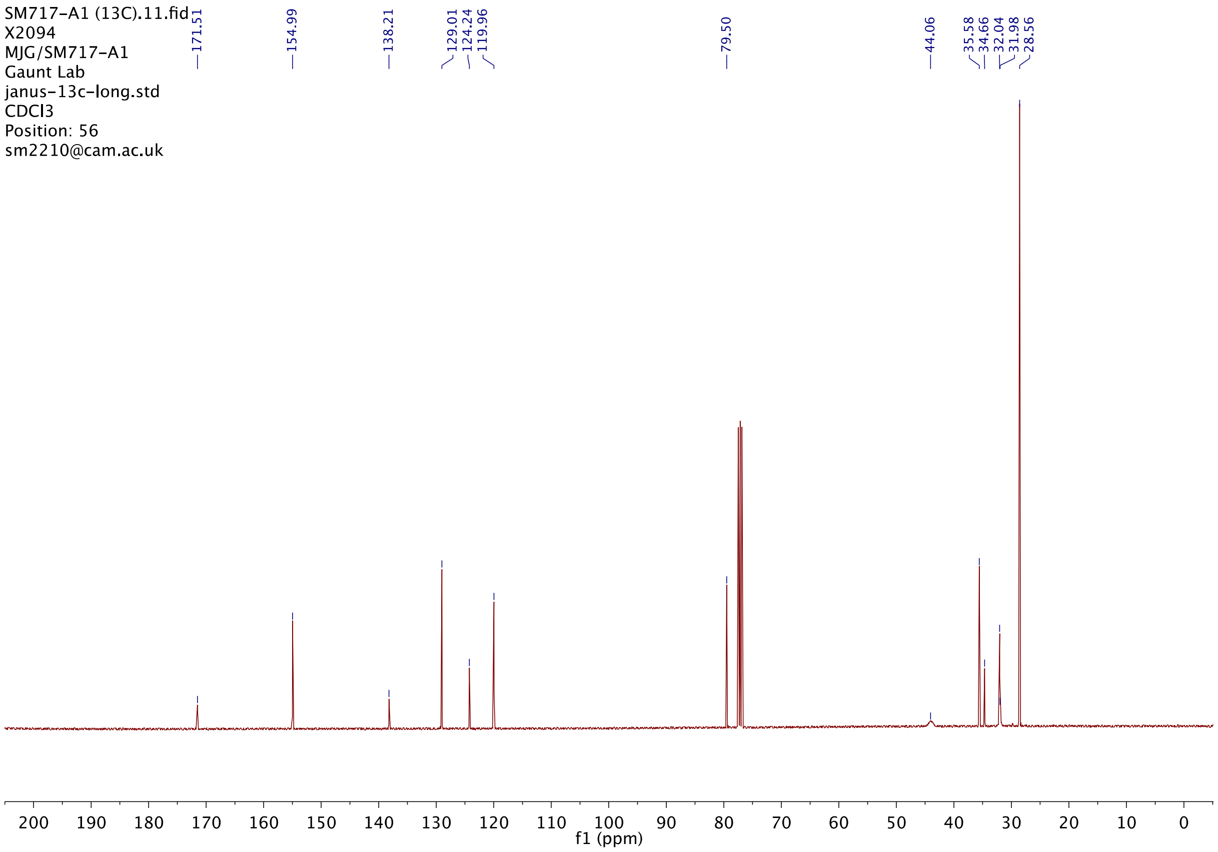
SM717-A1 (13C).11.fid
X2094
MJG/SM717-A1
Gaunt Lab
janus-13c-long.std
CDCl3
Position: 56
sm2210@cam.ac.uk
171.51
154.99
138.21
129.01
124.24
119.96
79.50
44.06
35.58
34.66
32.04
31.98
28.56
f1 (ppm)

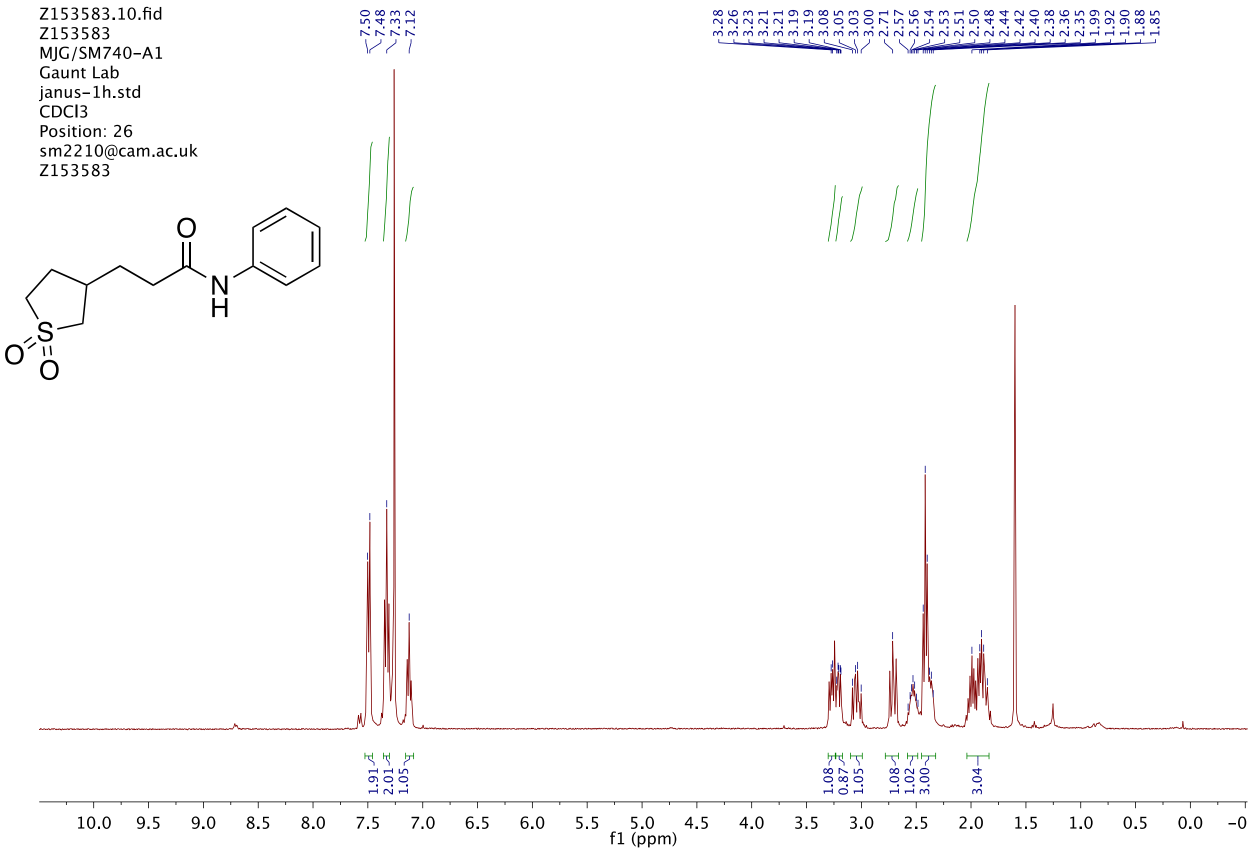
Z153583.10.fid
Z153583
MJG/SM740-A1
Gaunt Lab
janus-1h.std
CDCl3
Position: 26
sm2210@cam.ac.uk
Z153583
7.50
7.48
7.33
7.12
3.28
3.26
3.23
3.21
3.21
3.19
3.19
3.08
3.05
3.03
3.00
2.71
2.57
2.56
2.54
2.53
2.51
2.50
2.48
2.44
2.42
2.40
2.38
2.36
2.35
1.99
1.92
1.90
1.88
1.85
1.91
2.01
1.05
1.08
0.87
1.05
1.08
1.02
3.00
3.04
f1 (ppm)

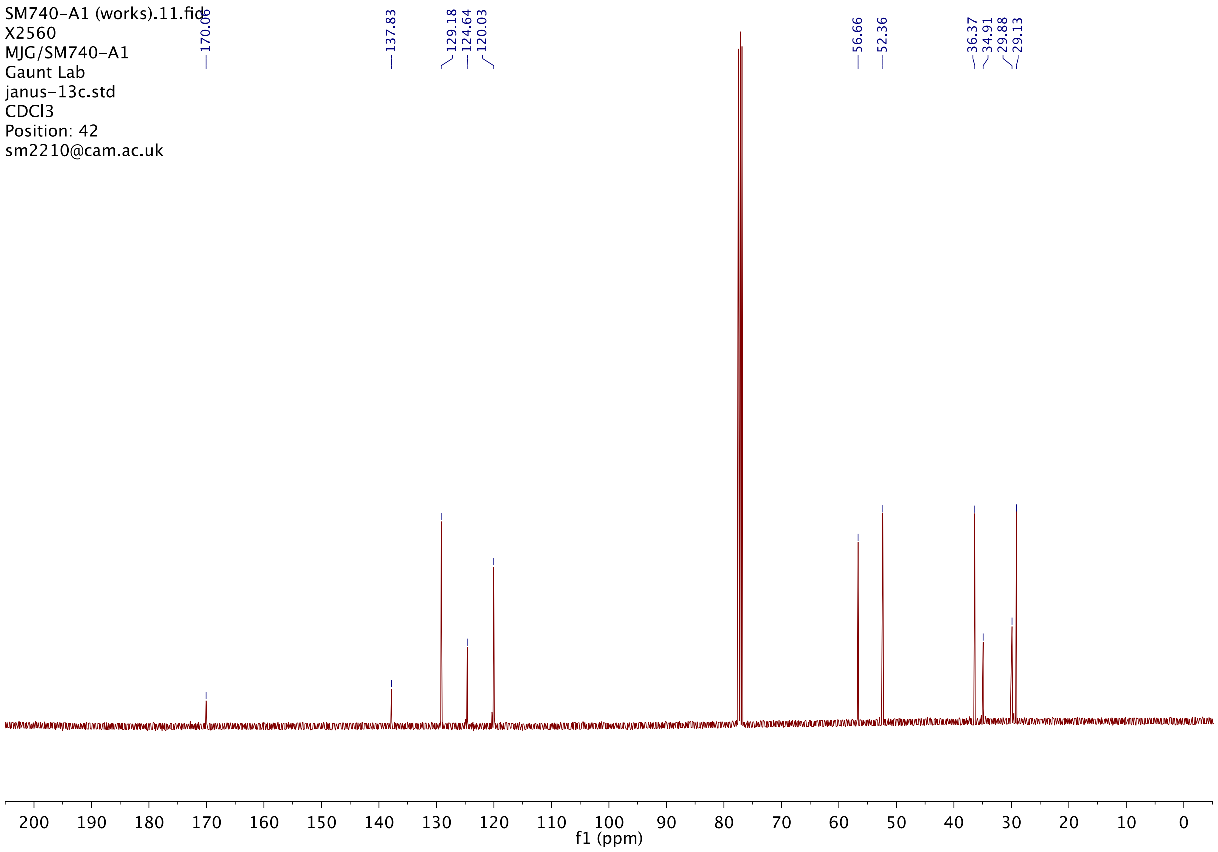
SM740-A1 (works).11.fid
X2560
MJG/SM740-A1
Gaunt Lab
janus-13c.std
CDCl3
Position: 42
sm2210@cam.ac.uk
170.06
137.83
129.18
124.64
120.03
56.66
52.36
36.37
34.91
29.88
29.13
f1 (ppm)

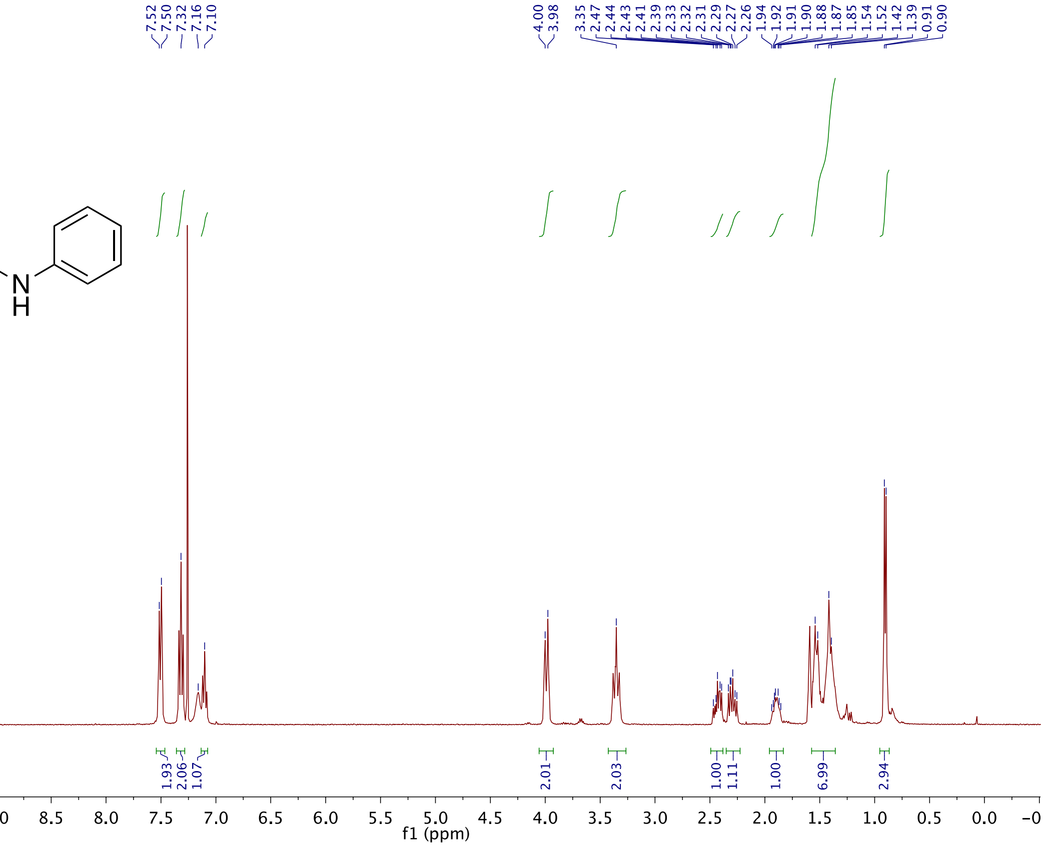
V351295.10.fid
V351295
MJG/SM721-A1
`Gaunt Lab
janus-1h.qnp
CDCl3
Position: 59
sm2210@cam.ac.uk
V351295
7.52 7.50 7.32 7.16 7.10
4.00 3.98 3.35 2.47 2.44 2.43 2.41 2.39 2.33 2.32 2.31 2.29 2.27 2.26 1.94 1.92 1.91 1.90 1.88 1.87 1.85 1.54 1.52 1.42 1.39 0.91 0.90
1.93 2.06 1.07 2.01 2.03 1.00 1.11 1.00 6.99 2.94
f1 (ppm)

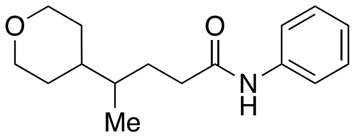
O
O
N
H
Me

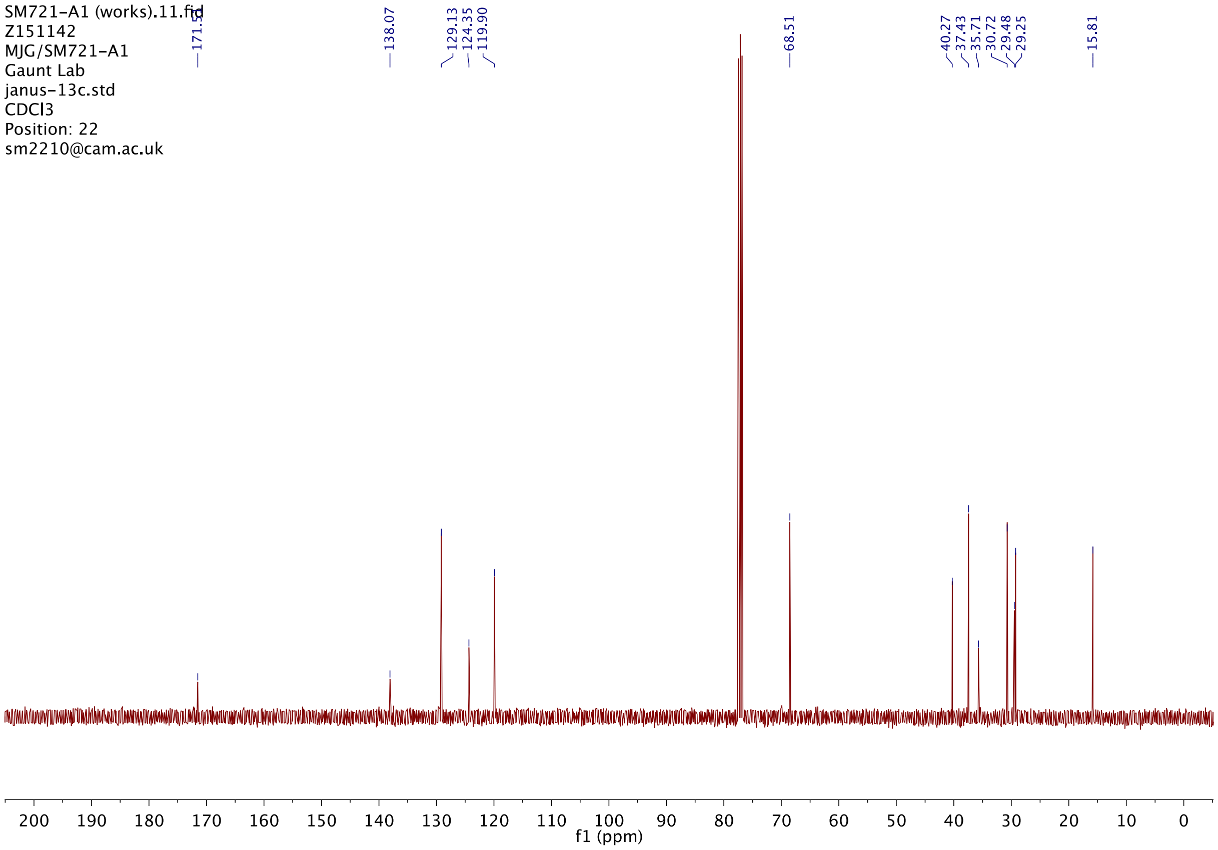
SM721-A1 (works).11.fid
Z151142
MJG/SM721-A1
Gaunt Lab
janus-13c.std
CDCl3
Position: 22
sm2210@cam.ac.uk
138.07
129.13 124.35 119.90
68.51
40.27 37.43 35.71 30.72 29.48 29.25
15.81
f1 (ppm)

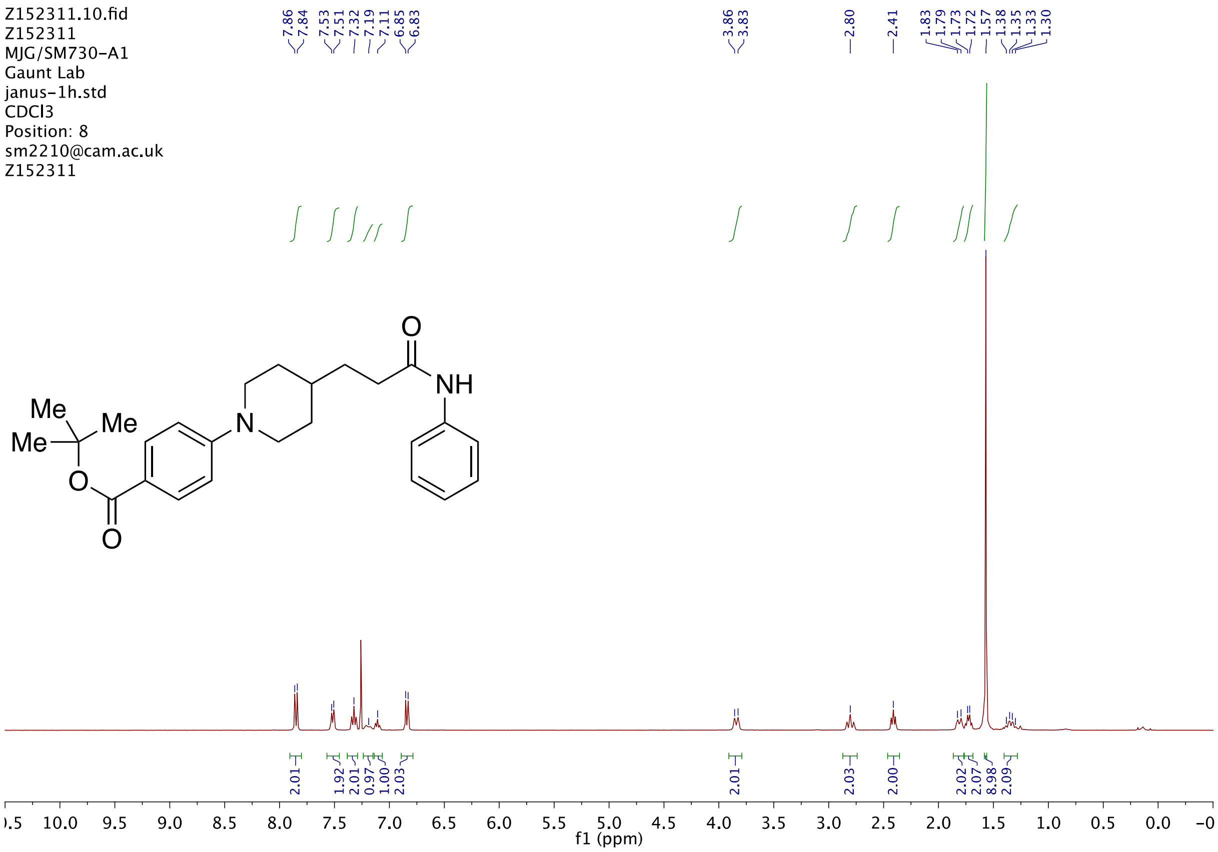
Z152311.10.fid
Z152311
MJG/SM730-A1
Gaunt Lab
janus-1h.std
CDCl3
Position: 8
sm2210@cam.ac.uk
Z152311
7.86
7.84
7.53
7.51
7.32
7.19
7.11
6.85
6.83
3.86
3.83
2.80
2.41
1.83
1.79
1.73
1.72
1.57
1.38
1.35
1.33
1.30
O
NH
N
Me
Me
Me
O
O
2.01
1.92
2.01
0.97
1.00
2.03
2.01
2.03
2.00
2.02
2.07
8.98
2.09
f1 (ppm)

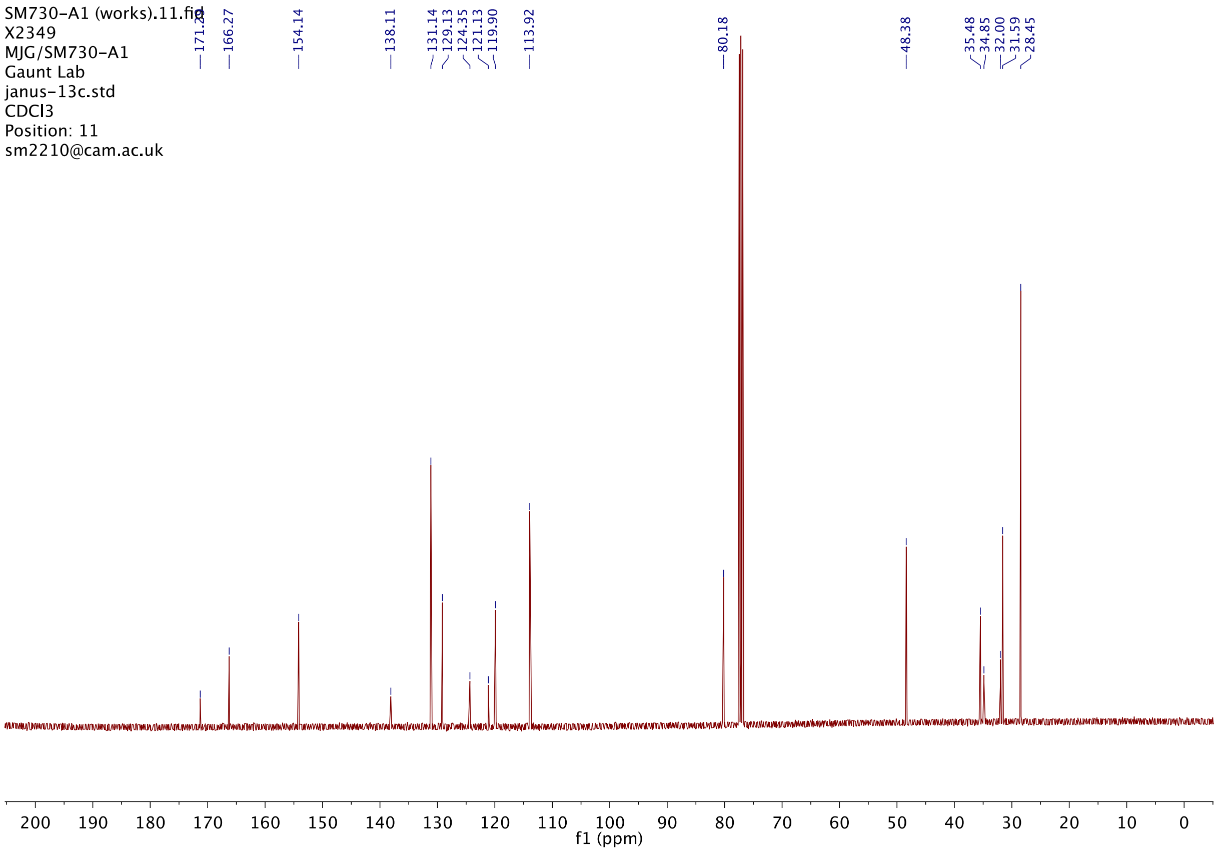
SM730-A1 (works).11.fid
X2349
MJG/SM730-A1
Gaunt Lab
janus-13c.std
CDCl3
Position: 11
sm2210@cam.ac.uk
166.27
154.14
138.11
131.14
129.13
124.35
121.13
119.90
113.92
80.18
48.38
35.48
34.85
32.00
31.59
28.45
f1 (ppm)

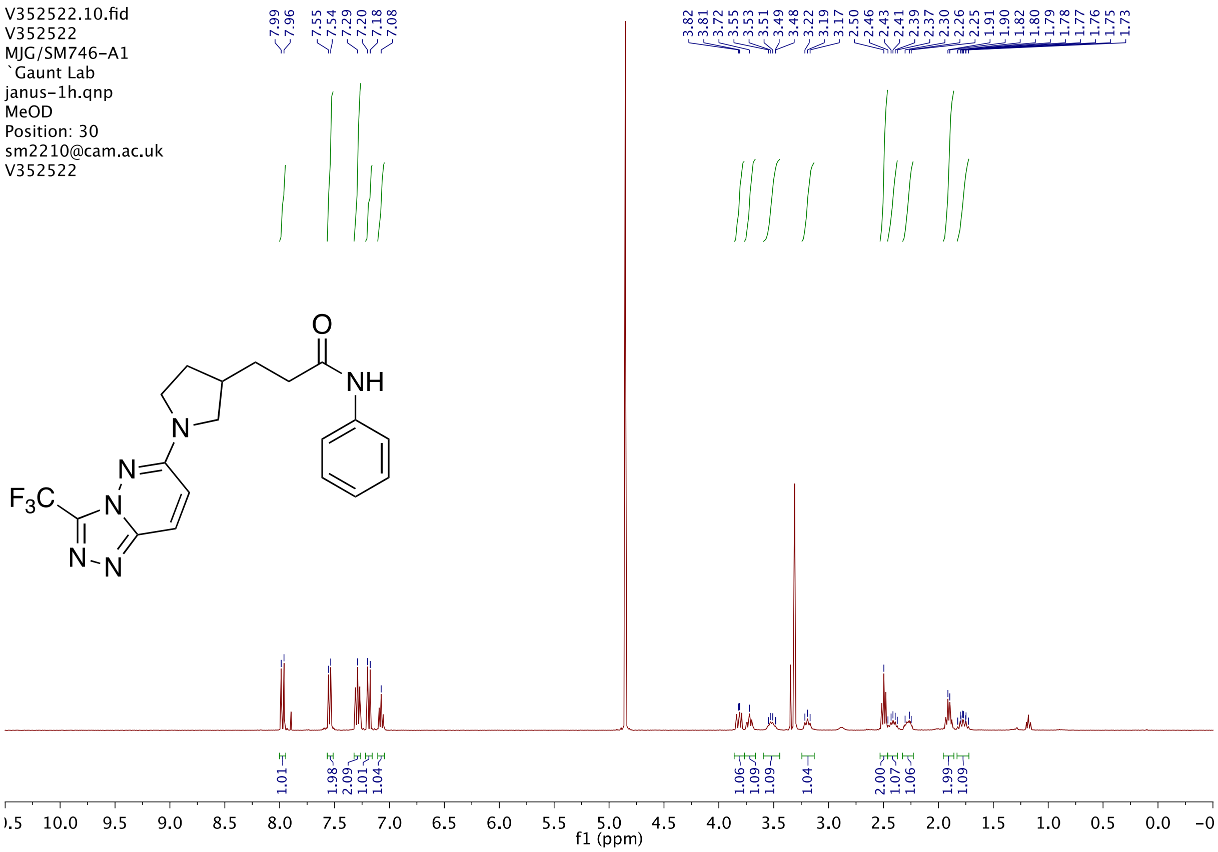
V352522.10.fid
V352522
MJG/SM746-A1
`Gaunt Lab
janus-1h.qnp
MeOD
Position: 30
sm2210@cam.ac.uk
V352522
7.99 7.96 7.55 7.54 7.29 7.20 7.18 7.08
3.82 3.81 3.72 3.55 3.53 3.51 3.49 3.48 3.22 3.19 3.17 2.50 2.46 2.43 2.41 2.39 2.37 2.30 2.26 2.25 1.91 1.90 1.82 1.80 1.79 1.78 1.77 1.76 1.75 1.73
O
NH
N
N
F3C
N
N
N
1.01 1.98 2.09 1.01 1.04 1.06 1.09 1.09 1.04 2.00 1.07 1.06 1.99 1.09
f1 (ppm)

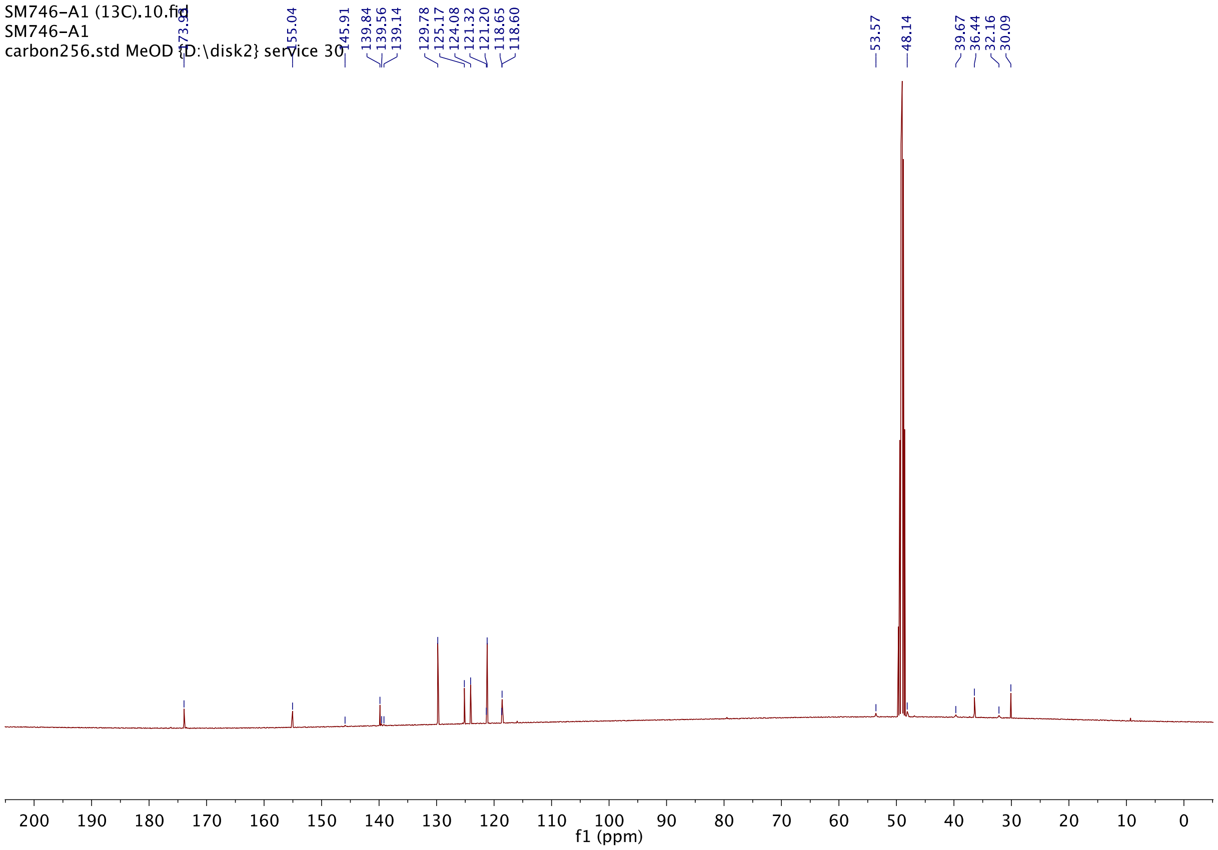
SM746-A1 (13C).10.fid
SM746-A1
carbon256.std MeOD {D:\disk2} service 30
173.93 155.04 145.91 139.84 139.56 139.14 129.78 125.17 124.08 121.32 121.20 118.65 118.60
53.57 48.14 39.67 36.44 32.16 30.09
f1 (ppm)

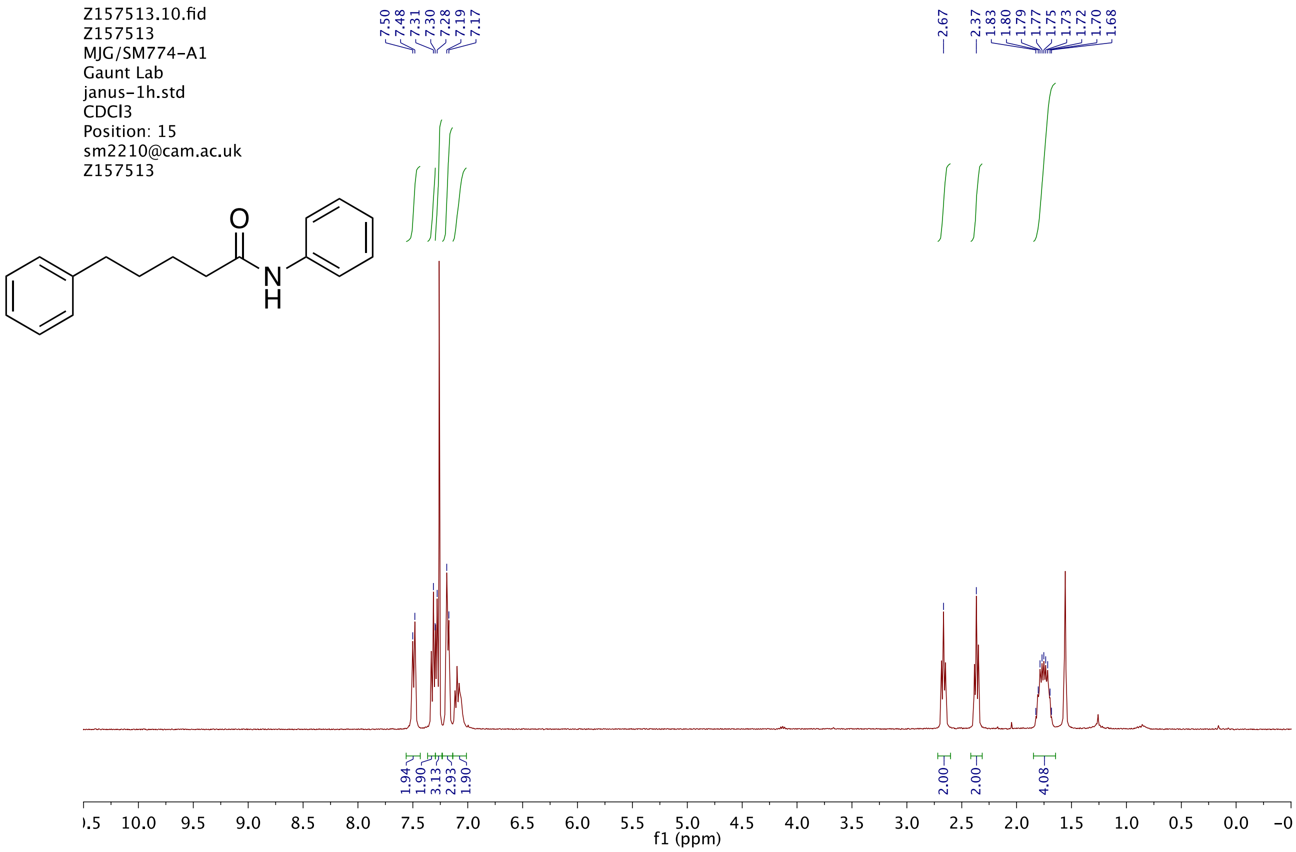
Z157513.10.fid
Z157513
MJG/SM774-A1
Gaunt Lab
janus-1h.std
CDCl3
Position: 15
sm2210@cam.ac.uk
Z157513
7.50
7.48
7.31
7.30
7.28
7.19
7.17
2.67
2.37
1.83
1.80
1.79
1.77
1.75
1.73
1.72
1.70
1.68
O
N
H
1.94
1.90
3.13
2.93
1.90
2.00
2.00
4.08
f1 (ppm)

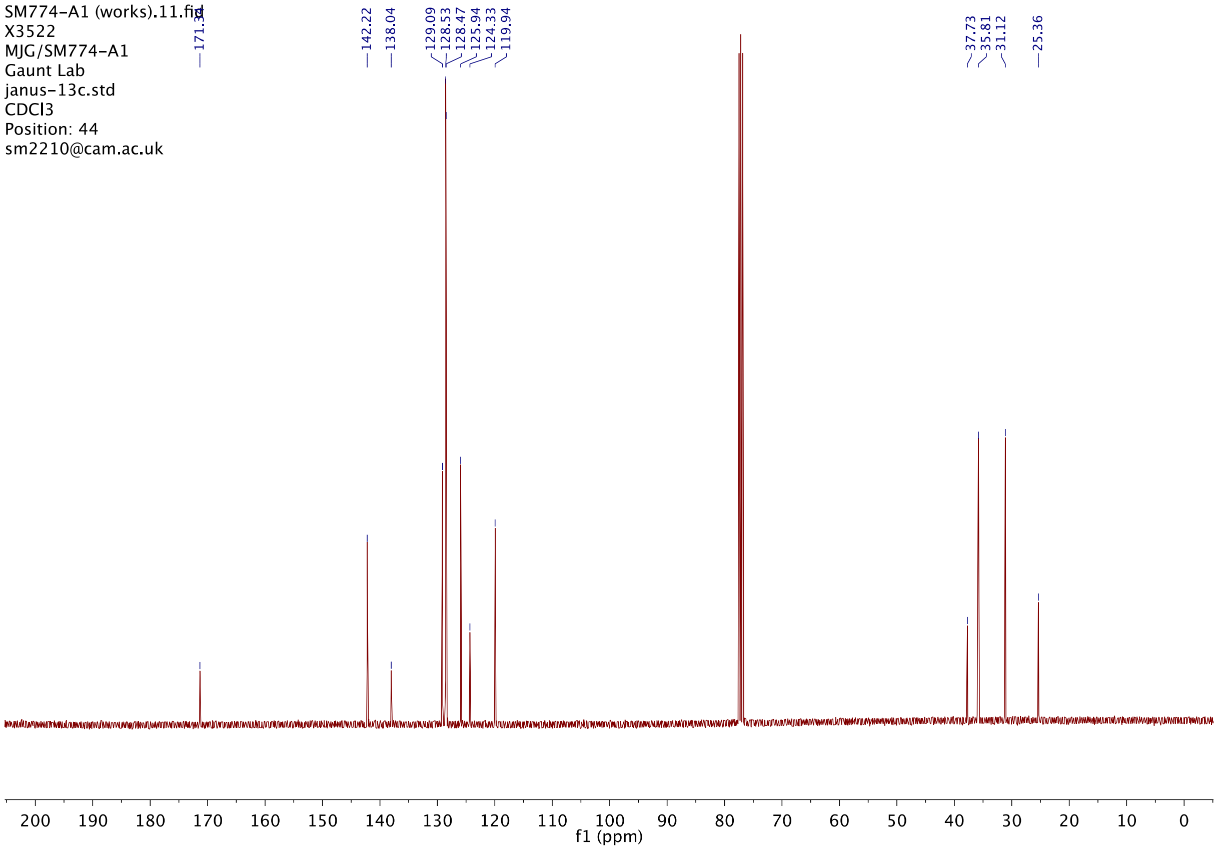
SM774-A1 (works).11.fid
X3522
MJG/SM774-A1
Gaunt Lab
janus-13c.std
CDCl3
Position: 44
sm2210@cam.ac.uk
171.3
142.22
138.04
129.09
128.53
128.47
125.94
124.33
119.94
37.73
35.81
31.12
25.36
f1 (ppm)

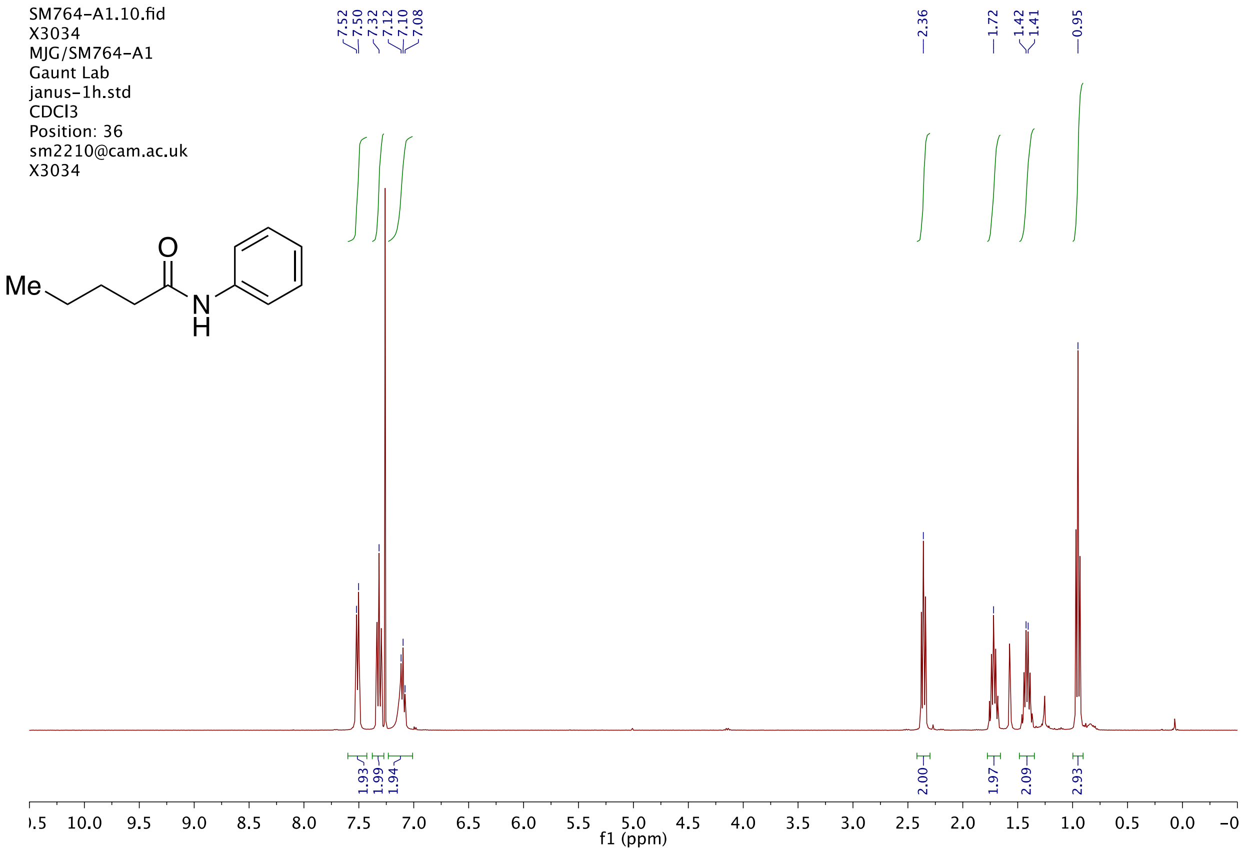
SM764-A1.10.fid
X3034
MJG/SM764-A1
Gaunt Lab
janus-1h.std
CDCl3
Position: 36
sm2210@cam.ac.uk
X3034
7.52
7.50
7.32
7.12
7.10
7.08
2.36
1.72
1.42
1.41
0.95
Me
O
N
H
1.93
1.99
1.94
2.00
1.97
2.09
2.93
f1 (ppm)

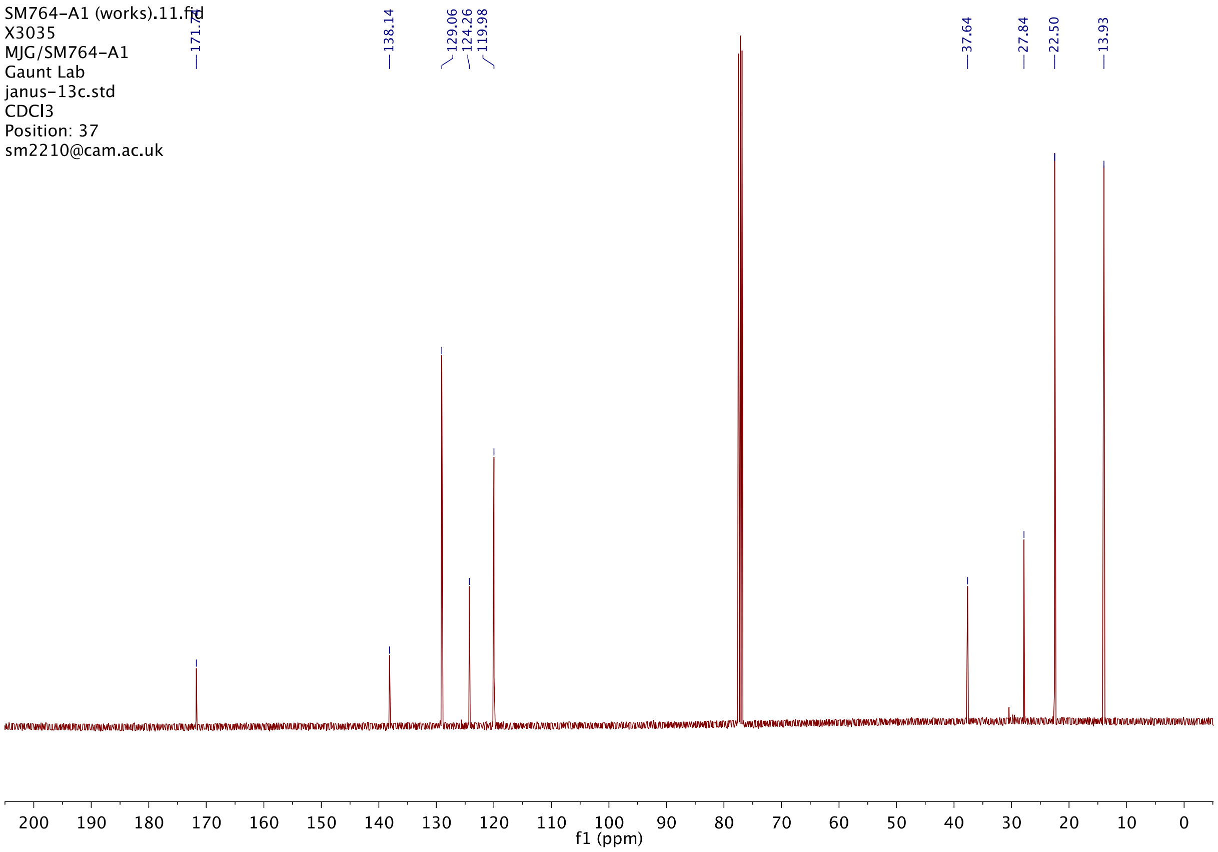
SM764-A1 (works).11.fid
X3035
MJG/SM764-A1
Gaunt Lab
janus-13c.std
CDCl3
Position: 37
sm2210@cam.ac.uk
138.14
129.06
124.26
119.98
37.64
27.84
22.50
13.93
f1 (ppm)

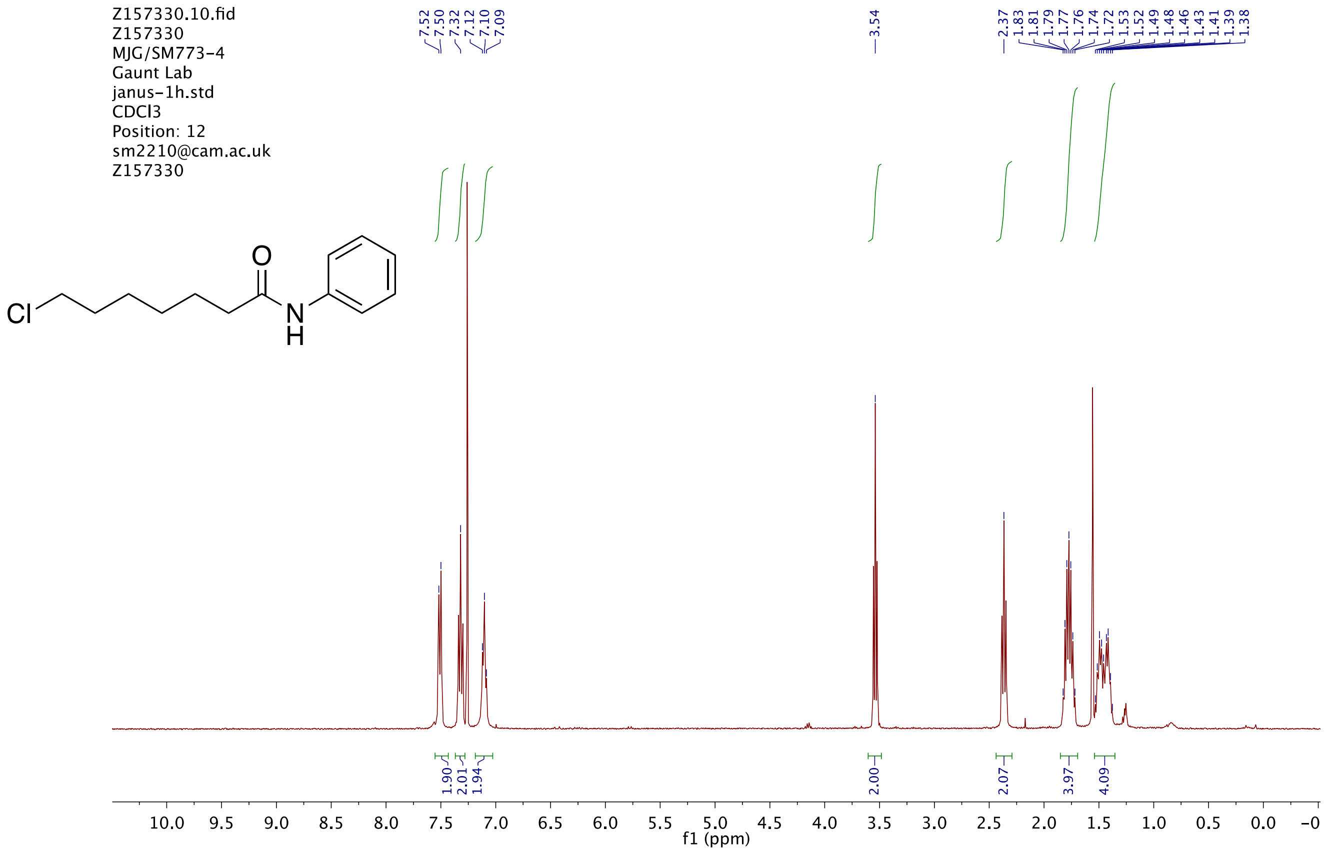
Z157330.10.fid
Z157330
MJG/SM773-4
Gaunt Lab
janus-1h.std
CDCl3
Position: 12
sm2210@cam.ac.uk
Z157330
7.52 7.50 7.32 7.12 7.10 7.09
3.54
2.37 1.83 1.81 1.79 1.77 1.76 1.74 1.72 1.53 1.52 1.49 1.48 1.46 1.43 1.41 1.39 1.38
Cl
O
N
H
1.90 2.01 1.94
2.00
2.07
3.97
4.09
10.0 9.5 9.0 8.5 8.0 7.5 7.0 6.5 6.0 5.5 5.0 4.5 4.0 3.5 3.0 2.5 2.0 1.5 1.0 0.5 0.0 -0
f1 (ppm)

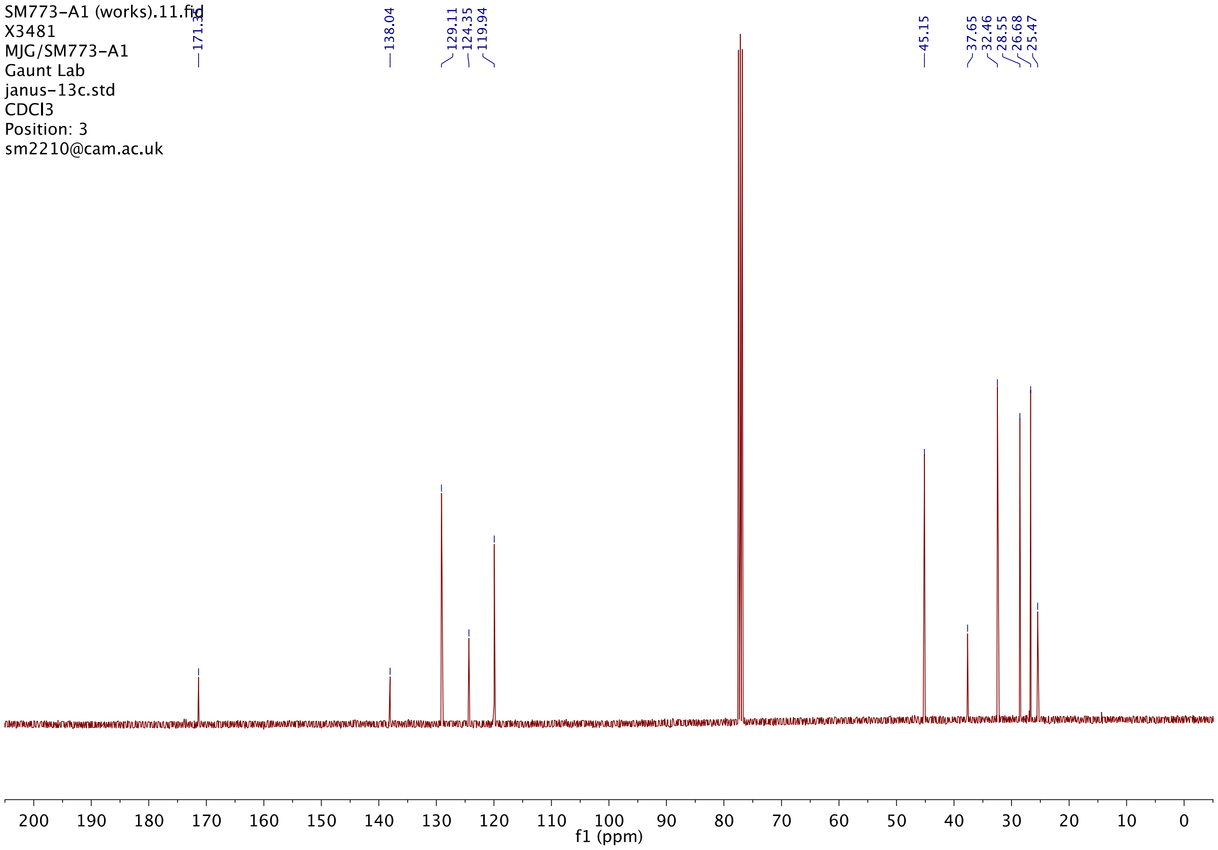
SM773-A1 (works).11.fid
X3481
MJG/SM773-A1
Gaunt Lab
janus-13c.std
CDCl3
Position: 3
sm2210@cam.ac.uk
138.04
129.11 124.35 119.94
45.15
37.65 32.46 28.55 26.68 25.47
200 190 180 170 160 150 140 130 120 110 100 90 80 70 60 50 40 30 20 10 0
f1 (ppm)

Radical Clock Experiment

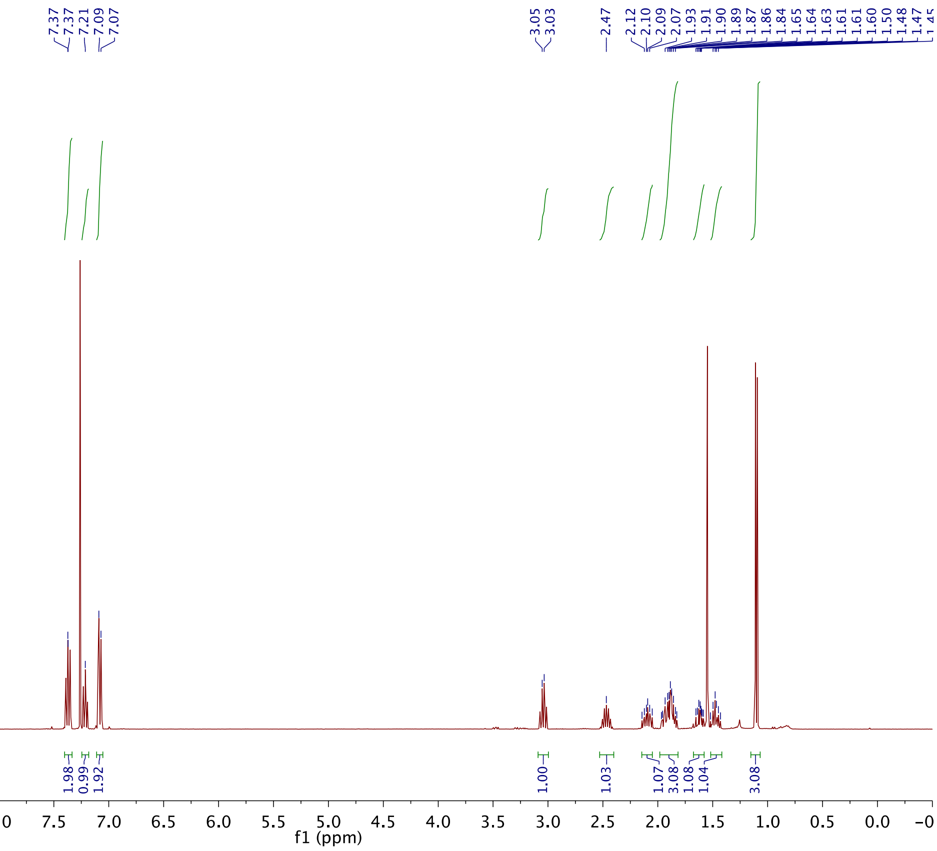

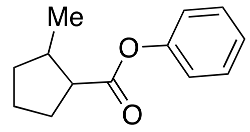

Major diasteroisomer

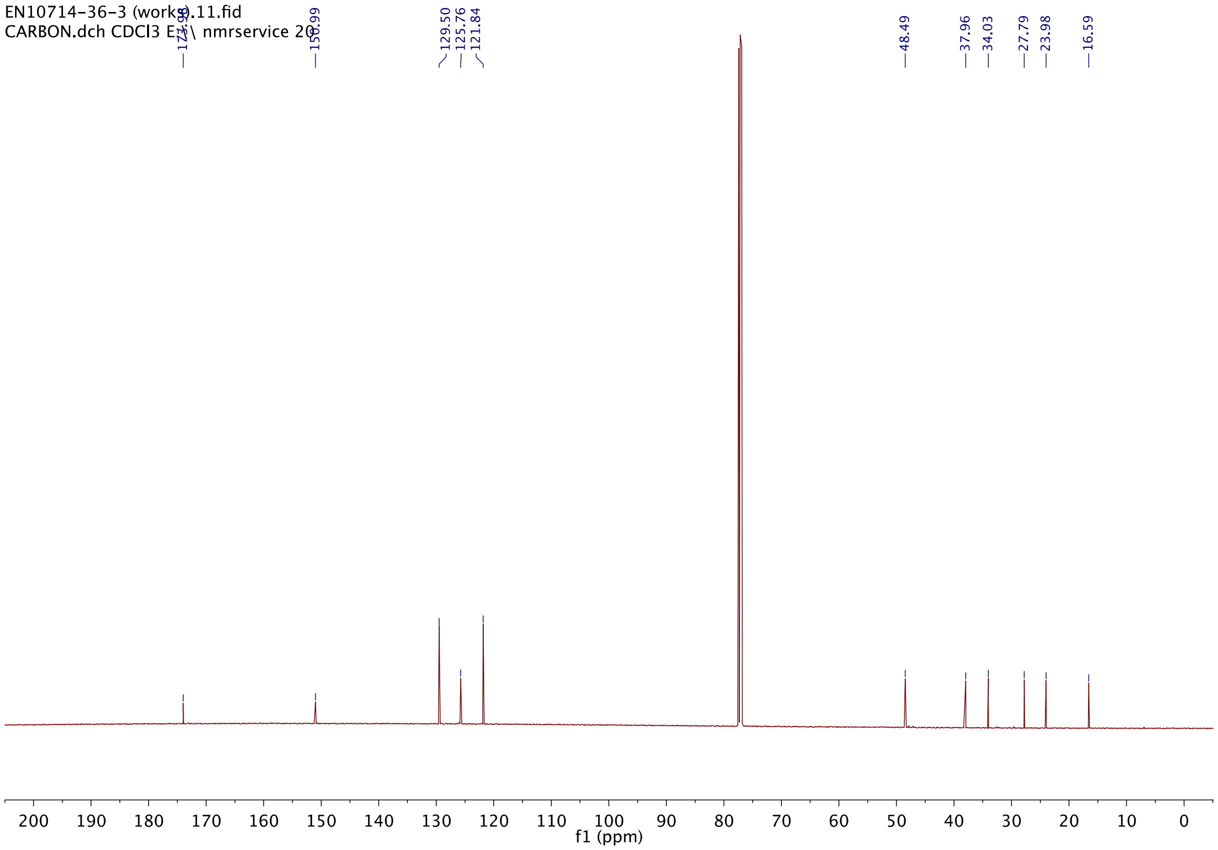

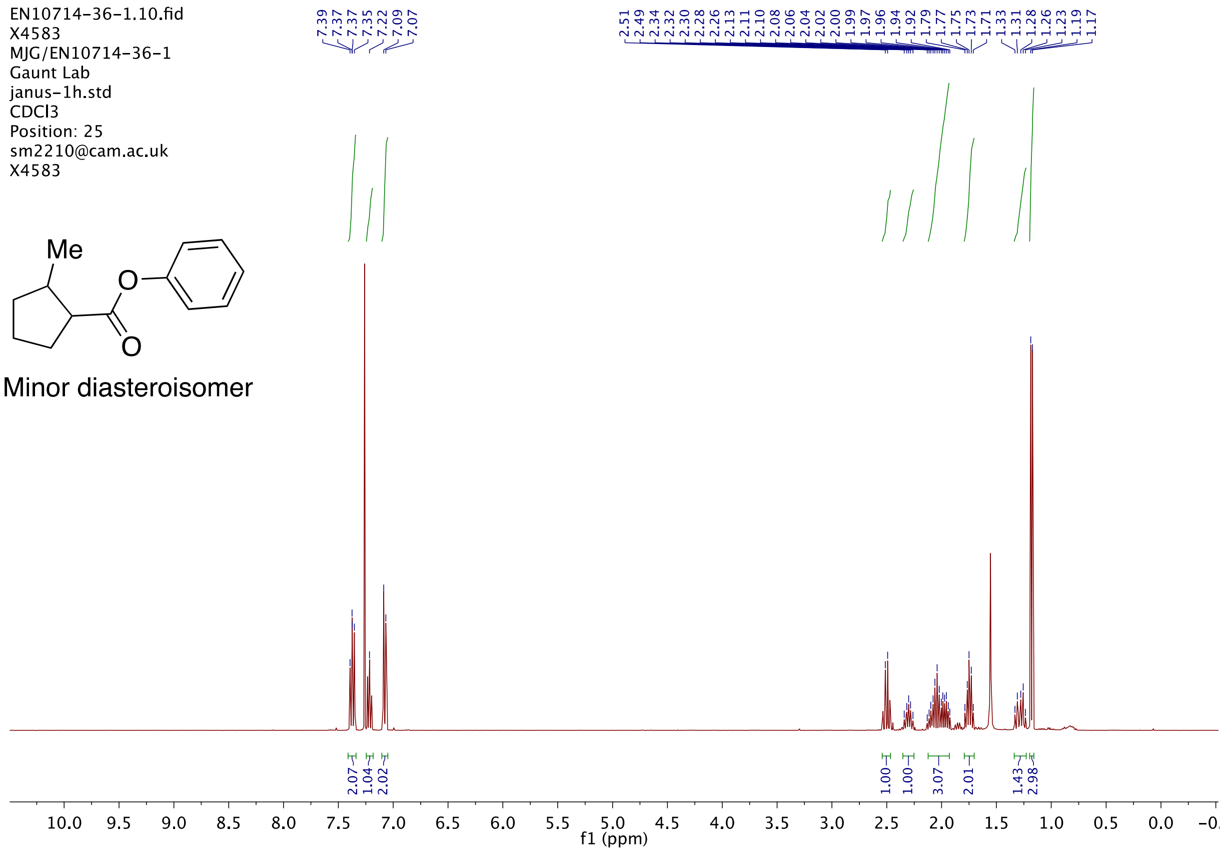
EN10714-36-1.10.fid
X4583
MJG/EN10714-36-1
Gaunt Lab
janus-1h.std
CDCl3
Position: 25
sm2210@cam.ac.uk
X4583
Me
O
O
Minor diastereoisomer
7.39 7.37 7.37 7.35 7.22 7.09 7.07
2.51 2.49 2.34 2.32 2.30 2.28 2.26 2.13 2.11 2.10 2.08 2.06 2.04 2.02 2.00 1.99 1.97 1.96 1.94 1.92 1.79 1.77 1.75 1.73 1.71 1.33 1.31 1.28 1.26 1.23 1.19 1.17
2.07 1.04 2.02 1.00 1.00 3.07 2.01 1.43 2.98
f1 (ppm)

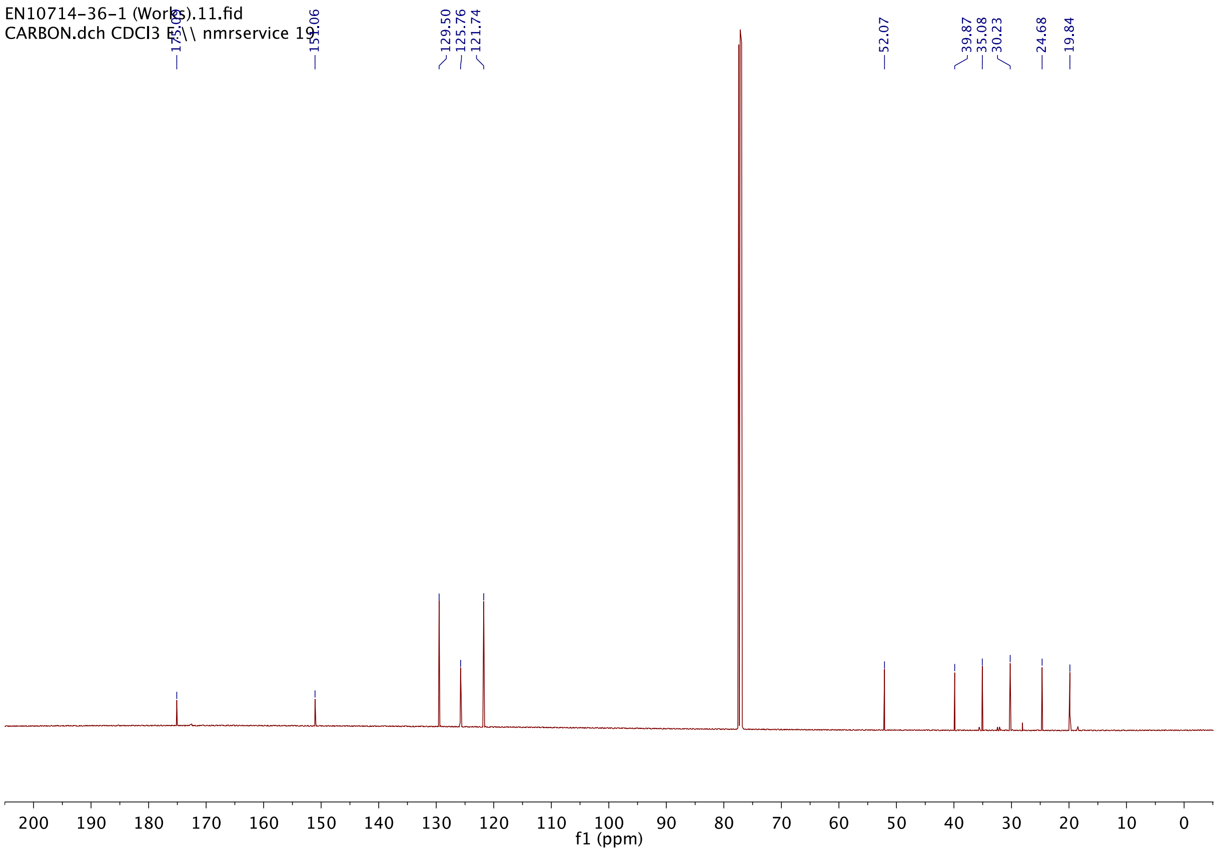
EN10714-36-1 (Works).11.fid
CARBON.dch CDCl3
nmrservice
151.06
129.50 125.76 121.74
52.07
39.87 35.08 30.23
24.68
19.84
f1 (ppm)
